# Design of non-ionic carbon superbases: second generation carbodiphosphoranes†
†Electronic supplementary information (ESI) available. CCDC 1903830, 1903833, 1903838, 1903840, 1903841 and 1903843. For ESI and crystallographic data in CIF or other electronic format see DOI: 10.1039/c9sc03565f


**DOI:** 10.1039/c9sc03565f

**Published:** 2019-08-16

**Authors:** Sebastian Ullrich, Borislav Kovačević, Björn Koch, Klaus Harms, Jörg Sundermeyer

**Affiliations:** a Fachbereich Chemie , Philipps-University Marburg , Hans-Meerwein-Straße , 35032 Marburg , Germany . Email: jsu@staff.uni-marburg.de; b The Group for Computational Life Sciences , Rudjer Bošković Institute , Bijenička c. 54 , HR-10000 Zagreb , Croatia

## Abstract

A new generation of carbodiphosphoranes (CDPs), incorporating pyrrolidine, tetramethylguanidine, or tris(dimethylamino)phosphazene as substituents is introduced as the most powerful class of non-ionic carbon superbases on the basicity scale to date. The synthetic approach as well as NMR spectroscopic and structural characteristics in the free and protonated form are described. Investigation of basicity in solution and in the gas phase by experimental and theoretical means provides the to our knowledge first reported p*K*_BH_^+^ values for CDPs in the literature and suggest them as upper tier superbases.

## Abstract 2

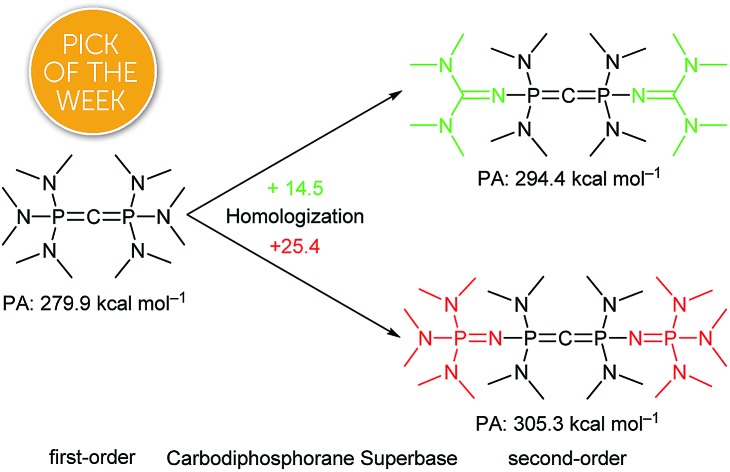
The edge-cutting design, synthesis and characterization of the so far strongest non-ionic carbon superbases is presented.

## Introduction

Much theoretical and synthetical effort has been devoted to lift non-ionic organic bases to the basicity level of common inorganic or metalorganic bases.^1^,^2^ With his famous phosphazenes Schwesinger established a widely used and commercially available class of (organo-)superbases.^3^,^4^ His homologization concept, the stepwise expansion of the molecular scaffold in order to better delocalize the positive charge formed upon protonation, was also applied to synthesize higher-order N-superbases of guanidines,^5^,^6^ imidazolidine amines^7^ and cyclopropeneimines.^8^,^9^ However, such basicity enhancement is accompanied by an unwanted growth of the bases' molecular weight. Therefore, other strategies for augmenting the intrinsic proton affinity have been investigated: in proton sponges, a second nitrogen basicity centre in close proximity to the first one increases the p*K*_BH_^+^ value up to 16 orders of magnitude by intramolecular hydrogen bonding compared to corresponding non chelating bases.^10^ Additional thermodynamic driving force comes from relief of strain of the aromatic backbone.^11^ Many derivatives of such proton sponges were designed by combining aforementioned superbasic functionalities with the 1,8-diaminonaphthalene structural motif^12^ or as proton pincers with different backbones.^13^

Atoms other than nitrogen as basicity centre were also applied, such as phosphorus.^14^,^15^ Recently, we demonstrated, that *N*-phosphazenyl substituted phosphines (PAPs) possess higher p*K*_BH_^+^ values as P^III^ bases than their corresponding phosphazene P^V^N*t*Bu counterparts as N bases.^16^ So far the limit of homologization is reached at the P_7_ level both in phosphazenyl phosphazenes and phosphazenyl phosphines as both P_7_ benchmark bases have only been isolated in their protonated form.^16^,^17^

Non-ionic carbon is another contender to extend the basicity ladder to unmatched regions.^18^ In this respect phosphorus (mono-)ylides^19^,^20^ as well as bisylidic proton sponges^21^ were investigated on theoretical and experimental level. Although identified as potential superbases, the application of *N*-heterocyclic carbenes (NHCs),^22^ cyclic alkyl amino carbenes (CAACs),^23^ carbodicarbenes (CDCs),^24^ and carbodiphosphoranes (CDPs)^25^ has been exploited predominantly as strong Lewis bases towards transition and main group elements other than the proton.^26^

The prototypic hexaphenyl carbodiphosphorane ((Ph)_6_-CDP) was first synthesized 1961 by Ramirez *et al.*^27^ Further compounds like the hexamethyl carbodiphosphorane ((Me)_6_-CDP),^28^ hexakis(dimethylamino) carbodiphosphorane ((dma)_6_-CDP),^29^ and mixed representatives followed.^30^–^32^

Herein we promote carbodiphosphoranes with their electron-rich R_3_P–C–PR_3_ functionality as exceptionally strong carbon Brønsted bases. As bisylides with a π-symmetric HOMO and σ-symmetric HOMO–1, both mainly located as lone pairs at the carbon, only slightly stabilized by backbonding *via* negative hyperconjugation,^33^ they provide outstanding p*K*_BH_^+^ values in particular for the first of two protonation steps. We present a synthesis for hexa(pyrrolidino) carbodiphosphorane ((pyrr)_6_-CDP) with its calculated first and second proton affinity (PA) of 287.6 and 188.9 kcal mol^–1^,^34^ which exceeds the PAs of (Ph)_6_-CDP (280.0 and 185.6 kcal mol^–1^)^34^ and (dma)_6_-CDP (279.9 and 174.9 kcal mol^–1^).^34^ Furthermore we apply the homoligization concept to CDPs by introducing PR_2_R′ units bearing one intrinsically superbasic substituent R′ to access CDP superbases of second-order.^8^ We thereby focused on *N*-tetramethylguanidinyl (tmg) and *N-*tris(dimethylamino)phosphazenyl (dmaP_1_) substituents targeting new carbodiphosphoranes *sym*-(tmg)_2_(dma)_4_-CDP and *sym*-(dmaP_1_)_2_(dma)_4_-CDP.

## Results and discussion

### Synthesis

**Scheme 1:**
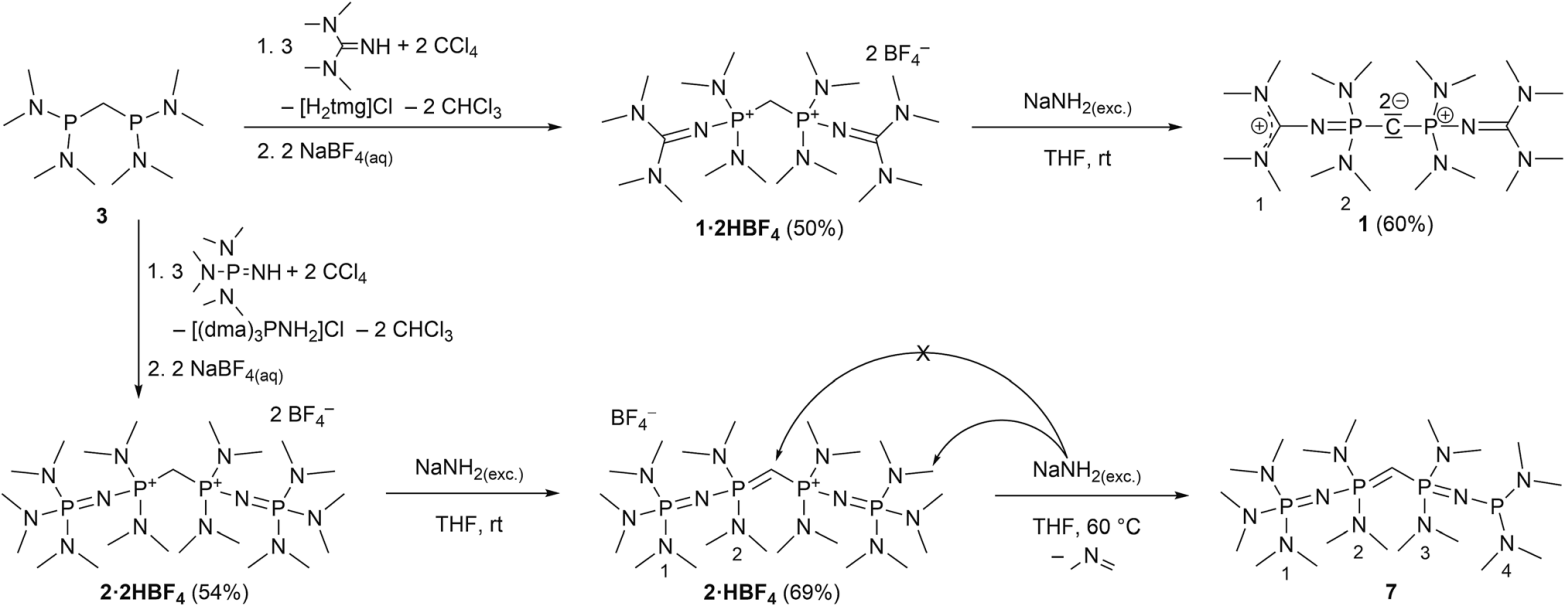
Preparation of CDP precursors **1·2HBF_4_** and **2·2HBF_4_** together with subsequent deprotonation to **1** (one exemplary mesomeric structure displayed) and **7**, respectively. Numbering schemes refer to assigned NMR signals in the experimental section.

We experienced, that the established synthesis routes to CDPs are inappropriate for phosphines more electron-rich than P(NMe_2_)_3_: reactions between such phosphines P(NR_2_)_2_R′ and CCl_4_ did not follow the pattern outlined in ref. 32 and 35 but exclusively led to chlorination of the phosphine, whilst reactions with methylene bromide did not selectively follow the path outlined in ref. 30 and 36, but led to a 1 : 1-mixture of the methylated phosphonium bromide [R′(NR_2_)_2_P-Me]Br and the brominated species [R′(NR_2_)_2_P-Br]Br. Therefore we further developed an alternative strategy laid out by Appel *et al.* for the synthesis of (dma)_6_-CDP.^29^ The doubly protonated precursors of the second-order carbodiphosphorane superbases, *sym*-(tmg)_2_(dma)_4_-CDP (**1**) and *sym*-(dmaP_1_)_2_(dma)_4_-CDP (**2**), were obtained in an oxidative imination sequence as shown in Scheme 1. Bis[bis(dimethylamino)phosphino]methane (**3**) was oxidized by CCl_4_ in presence of tetramethylguanidine (Htmg) or tris(dimethylamino)phosphazene ((dma)P_1_-H) instead of dimethylamine as nucleophile and auxiliary base. This reaction offers the advantage of preformed C–P-bonds avoiding the preparation of respective P^III^ nucleophiles.^15^,^20^,^37^ **3** is readily synthesized in two steps on a large scale^38^ and the selected superbasic building blocks oxidatively introduced as nucleophiles are either commercially available or easily accessible in few steps.^4^

**Scheme 2:**
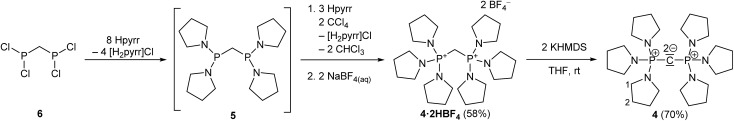
*In situ* preparation of **5** with subsequent oxidation by CCl_4_ in presence of excess of pyrrolidine (Hpyrr) to **4·2HBF_4_**. Deprotonation with KHMDS lead to the free CDP **4** (displayed in exemplarily bisylidic notation). The numbering scheme refers to assigned NMR signals in the experimental section.

The synthesis of **4·2HBF_4_**, the precursor for (pyrr)_6_-CDP **4**, was accomplished in a one-pot synthesis (Scheme 2), since the intermediate bis[di(pyrrolidino)phosphino]methane (**5**) turned out to decompose upon vacuum distillation. Starting from bis(dichlorophosphino)methane^38^ (**6**), **5** was prepared *in situ* with an excess of pyrrolidine (Fig. S1 in the ESI†) and directly oxidized with CCl_4_.

In all three reactions the respective monoprotonated hydrochloride adducts were identified as products *via*^31^P NMR spectroscopy. Therefore the second p*K*_BH_^+^ values in THF of these new CDPs are obviously lower than that of the auxiliary base pyrrolidine (13.5),^39^ tetramethylguanidine (15.5),^40^ or tris(dimethylamino)phosphazene **2a** (19.7),^40^ respectively. For purification, the crude products were precipitated with NaBF_4_ from aqueous solution. These conditions lead to second protonation at the central carbon atom and a strongly alkaline solution. Therefore, even the monoprotonated CDPs can be considered as strong cationic bases in aqueous medium. Similar behaviour was found for (Ph)_6_-CDP in water, although the latter is slowly hydrolysed under ambient conditions,^27^ which is not the case for peraminated CDPs **1**, **2** and **4** reported here.

The bis(tetrafluoridoborate) salts of **1**, **2** and **4** were obtained in 50–60% yield as water and air stable, colourless solids, indefinitely storable. They are well soluble in polar organic solvents like methanol, acetonitrile or DMSO but insoluble in less polar solvents such as ethers and hydrocarbons.

For the liberation of the free CDPs different suitable bases were identified: for **4** potassium bis(trimethylsilyl)amide (KHMDS) is of sufficient basicity, whilst for **1** the more basic sodium amide (NaNH_2_) is necessary for full deprotonation. Both new bases **1** and **4** could be isolated in 70% and 60% yield, respectively, from *n*-hexane as pure colourless crystalline solids, indefinitely storable at room temperature under inert conditions. Contrastingly we were not able to isolate **2** as free CDP base form. Sodium amide in liquid ammonia or suspended in THF at room temperature selectively abstracts the first proton under formation of **2·HBF_4_** as colourless solid in 69% yield. At elevated temperature the central carbon atom is not further deprotonated, even though it is the thermodynamically most acidic site (see Theoretical Calculations). Instead NaNH_2_ deprotonates selectively one of the dimethylamino groups at the terminal phosphazene moiety which results in the irreversible elimination of *N*-methylmethanimine and reduction of the phosphazene to a phosphine (Scheme 1). A related deprotonation and reduction of tetrakis(dimethylamino)phosphonium bromide under the action of NaNH_2_ was described by Pinchuk *et al.*^41^ In case of **2** this reaction is slow but highly selective and **7** could be obtained as sole product as pale yellow highly viscous oil. The proposed configuration was confirmed *via*^1^H, ^13^C, and ^31^P NMR spectroscopy and by HR mass spectrometry. **7** can be considered as a hybrid between mixed valence phosphazenyl phosphines^15^,^16^ and ylidic P^III^/P^V^ compounds of the type (Me_2_N)_3_P=C(H)–PR_2_ (ref. 42) or other ylide-functionalized phosphines.^43^ Further attempts to deprotonate **2·2HBF_4_** with other bases or reducing agents resulted either in only single deprotonation (benzyl potassium in THF), in an unselective disintegration (*n*BuLi) or in the same deprotonation of the P-NMe_2_ group (potassium in liquid ammonia, ethylene diamine, THF, or DME or an excess of benzyl potassium in THF). The reaction of potassium hydride in THF gave a mixture of **7** as minor component and presumably free CDP **2** as major product by means of ^31^P NMR spectroscopy (Fig. S29 in the ESI†). Clearly the acidity of P^V^-attached NMe_2_ groups limits the accessibility of **2**. Under the action of excess of strong inorganic bases at elevated temperatures the stability limit of these phosphazene moieties seems to have been reached.

For analytical reasons the monoprotonated forms of **1** and **4** were prepared on NMR scale either *via* commutation between the free CDP and its bisprotonated form or by protonating the free CDPs with one equivalent triflimidic acid (HTFSI).

### Structural features

**Fig. 1:**
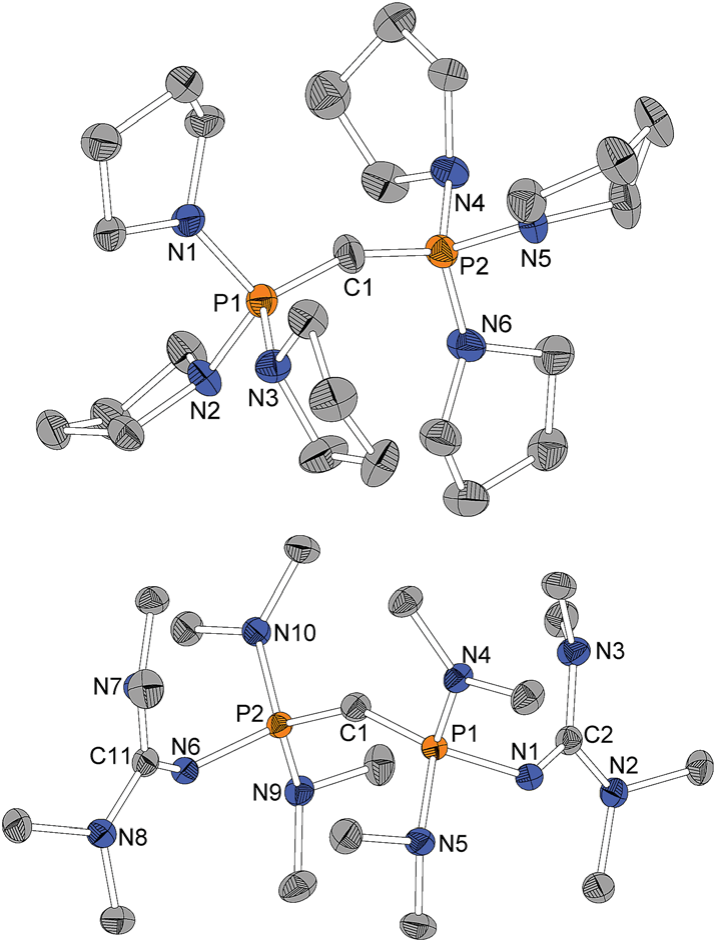
Molecular structure of **4** (top) and **1** (bottom). Hydrogen atoms omitted for clarity, ellipsoids at 50% probability. Selected bond length/Å and angles/°: **4** P1–C1 1.605(2), P1–N1 1.672(2), P1–N2 1.678(2), P1–N3 1.694(2), P2–C1 1.606(2), P2–N4 1.699(2), P2–N5 1.669(2), P2–N6 1.671(2), P1–C1–P2 155.9(2), C1–P1–N1 110.2(1), C1–P1–N2 115.1(1), C1–P1–N3 121.8(1), C1–P2–N4 118.4(1), C1–P2–N5 111.3(1), C1–P2–N6 117.1(1), N1–P1–C1–P2 168.0(4), N4–P2–C1–P1 130.6(4). **1** P1–C1 1.619(1), P1–N4 1.680(1), P1–N5 1.714(1), P1–N1 1.665(1), N1–C2 1.298(2), N2–C2 1.377(2), N3–C2 1.382(2), P2–C1 1.617(1), P2–N9 1.719(1), P2–N10 1.680(1), P2–N6 1.664(1), N6–C11 1.299(2), N7–C11 1.376(2), N8–C11 1.379(2), P2–C1–P1 147.30(9), C1–P1–N4 109.52(6), C1–P1–N5 121.56(6), C1–P1–N1 119.85(6), C2–N1–P1 128.1(1), C1–P2–N9 120.76(6), C1–P2–N10 110.08(6), C1–P2–N6 119.47(6), C11–N6–P2 127.3(1), N4–P1–C1–P2 162.2(2), N10–P2–C1–P1 155.8(2).

For X-ray structure determination suitable single crystals were obtained from *n*-hexane for both presented CDPs **4** and **1**. They crystallize solvent-free in space group *P*2_1_/*c* or *Pbca*, respectively, with one complete molecule per asymmetric unit (Fig. 1). Contrary to the parent compound (dma)_6_-CDP, one of the hitherto two reported linear CDPs,^29^,^44^ a bent structure with P–C–P angles of 155.9(2)° and 147.30(9)°, respectively is found. Since the potential for bending at the central P–C–P carbon atom in polymorphic (Ph)_6_-CDP is very flat^44^ and reveals high dependence of the crystallization method,^45^ the obtained crystals of (dma)_6_-CDP from the melt are maybe the reason for its linearity.^29^ The P–C_central_ distances are with 1.606 Å (**4**) and 1.618 Å (**1**) in the for CDPs reported range: (dma)_6_-CDP: 1.584(1) Å,^29^ (Me)_6_-CDP: 1.594(3) Å,^46^ (Ph)_6_-CDP: 1.601–1.635 Å.^44^,^47^ On average, pyrrolidine N–P distances in **4** are 1.68 Å while those of dma and tmg groups in **1** are 1.70 Å and 1.66 Å respectively.

**Fig. 2:**
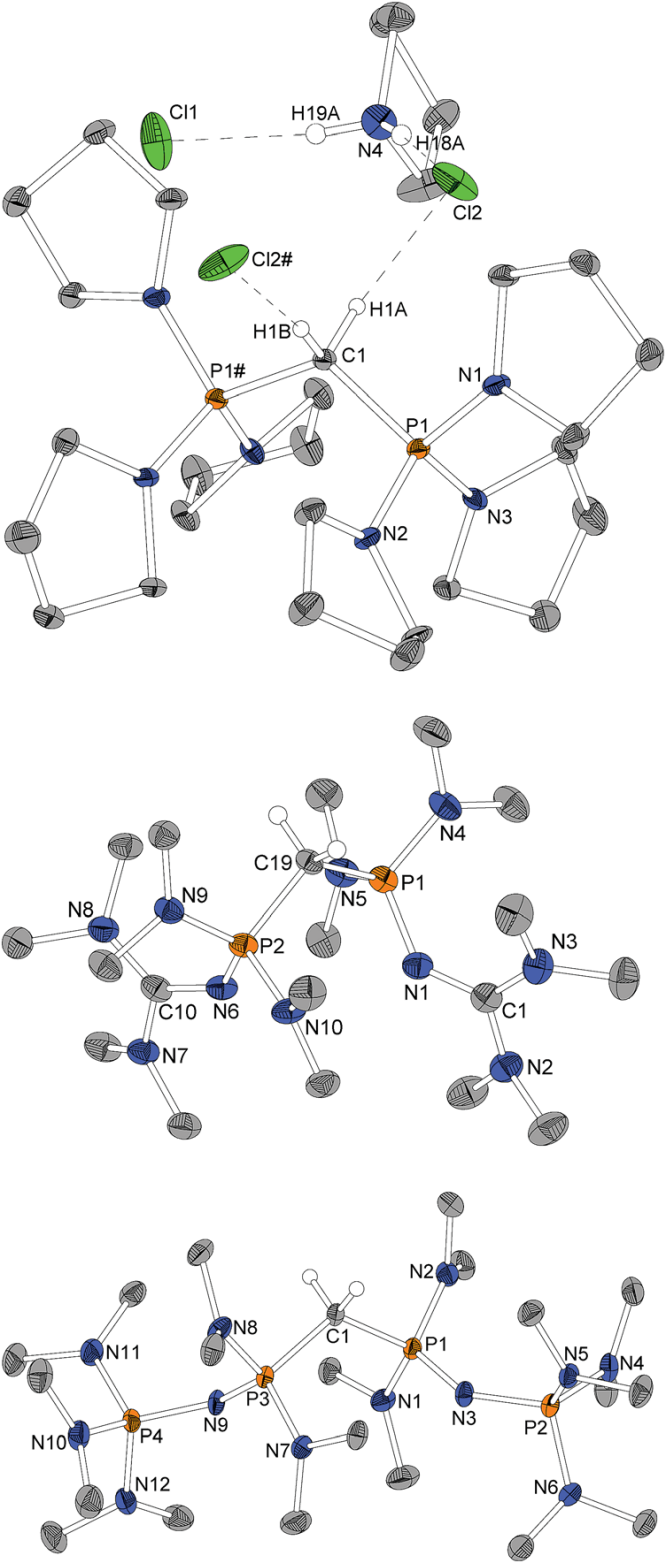
Molecular structure of **4·2HCl** with pyrrolidinium chloride as cocrystallizate as well as of **1·2HBF_4_** and **2·2HBF_4_** (only one of the two independent molecules depicted, structure factors given for both). Peripheral hydrogen atoms and BF_4_-anions omitted for clarity, ellipsoids at 50% probability. # marked atoms generated *via* a 2-fold axes through C1. Selected bond length/Å and angles/°: **4·2HCl** P1–C1 1.799(1), P1–N1 1.612(2), P1–N2 1.630(2), P1–N3 1.616(2), P1–C1–P1# 119.5(1), N1–P1–C1 103.26(9), N2–P1–C1 109.07(7), N3–P1–C1 115.21(8), N1–P1–C1–P1# 177.03(7), C1–H1A···Cl2 3.600(2), 173.8; C1–H1B···Cl2# 3.600(2), 173.8; N4–H18A···Cl2 3.048(2), 174(3); N4–H19A···Cl1 3.018(2), 172(3). **1·2HBF_4_** P1–C19 1.820(2), P1–N4 1.644(2), P1–N5 1.639(2), P1–N1 1.580(2), N1–C1 1.330(3), N2–C1 1.351(3), N3–C1 1.346(3), P2–C19 1.822(2), P2–N9 1.640(2), P2–N10 1.643(2), P2–N6 1.586(2), N6–C10 1.335(3), N7–C10 1.332(3), N8–C10 1.349(3), P1–C19–P2 113.4(1), N4–P1–C19 104.3(1), N5–P1–C19 109.7(1), N1–P1–C19 110.8(1), C1–N1–P1 136.1(2), N9–P2–C19 105.4(1), N10–P2–C19 108.8(1), N6–P2–C19 111.8(1), C10–N6–P2 132.6(2), N4–P1–C19–P2 169.1(1), N9–P2–C19–P1 165.2(1). **2·2HBF_4_** P1–C1/P5–C22 1.820(4)/1.822(4), P1–N1/P5–N19 1.626(4)/1.630(4), P1–N2/P5–N20 1.642(4)/1.650(4), P1–N3/P5–N21 1.573(4)/1.571(4), P2–N3/P6–N21 1.582(4)/1.589(4), P2–N4/P6–N22 1.648(4)/1.639(4), P2–N5/P6–N23 1.639(4)/1.639(4), P2–N6/P6–N24 1.650(4)/1.655(4), P3–C1/P7–C22 1.819(5)/1.817(5), P3–N7/P7–N13 1.636(4)/1.645(4), P3–N8/P7–N14 1.647(4)/1.635(4), P3–N9/P7–N15 1.575(4)/1.567(4), P4–N9/P8–N15 1.577(4)/1.579(4), P4–N10/P8–N16 1.644(4)/1.652(4), P4–N11/P8–N17 1.636(4)/1.648(4), P4–N12/P8–N18 1.655(4)/1.637(4), P3–C1–P1/P5–C22–P7 120.9(2)/121.7(2), N1–P1–C1/N19–P5–C22 110.8(2)/109.8(2), N2–P1–C1/N20–P5–C22 103.8(2)/104.0(2), N3–P1–C1/N21–P5–C22 107.9(2)/108.4(2), P1–N3–P2/P5–N21–P6 138.2(3)/135.7(3), N7–P3–C1/N14–P7–C22 111.2(2)/112.4(2), N8–P3–C1/N13–P7–C22 105.0(2)/103.2(2), N9–P3–C1/N15–P7–C22 107.9(2)/107.4(2), P3–N9–P4/P7–N15–P8 133.6(3)/141.6(3), N2–P1–C1–P3/N20–P5–C22–P7 164.8(3)/166.8(3), N8–P3–C1–P1/N13–P7–C22–P5 165.6(3)/164.4(3).

Single crystals obtained from reaction control samples during the synthesis of **4·2HBF_4_** turned out to be a cocrystallizate of **4·2HCl** and pyrrolidinium chloride (Fig. 2). Cations and anions form a C–H···Cl···H–N hydrogen bond network with C···Cl distances of 3.600(2) Å and N···Cl distances of 3.018(2) Å and 3.048(2) Å, the slightly longer distance involving the bridging chlorine atom. Similar weak hydrogen bonds were described for (Ph)_6_-CDP·2H^+^ with [InCl_4_]^–^ (3.60 Å and 4.03 Å),^48^ [BeCl_4_]^2–^ (3.55 Å and 3.58 Å),^49^ I^–^ (3.80 Å and 3.81 Å)^50^ and Cl^–^ (3.38 Å)^49^ anions. The difference between the latter and **4·2HCl** probably arise from a less polarized C–H-bond due to the stronger electron pair donor **4**. Single crystals of the isolated **4·2HBF_4_** were additionally obtained from chloroform and exhibits no significant differences in the structural properties (displayed in the ESI†). Fig. 2 shows the molecular structures of **1·2HBF_4_** and **2·2HBF_4_** as well. All three bisprotonated CDPs exhibit a strong influence of charge delocalization as the reason for their extraordinary basicity: upon protonation the P–C bonds elongate from 1.606 Å (**4**) and 1.618 Å (**1**) to 1.799 Å in **4·2HCl** and 1.821 Å in **1·2HBF_4_** and **2·2HBF_4_**, whilst the P–N bonds become shorter to average 1.62 Å for pyrrolidine and 1.64 Å for dimethylamine substituents. This complies with distances found in protonated phosphazenes^51^ and phosphorus ylids^52^ and proves the electron donating effect of the amino substituents. The P–N bonds to the tmg groups in **1·2HBF_4_** exhibits with 1.58 Å (1.66 Å in **1**) clearly double-bond character. The P–N=C angles are expanded from 127° and 128° to 132° and 136°. A diminishing difference of formal N–C single and double bonds in the tmg group indicates the conjugation within the CN_3_ moiety. The formal P–N single and double bonds of the phosphazene substituents in **2·2HBF_4_** equalize at 1.57–1.59 Å with P–N–P angles between 134° and 142°. Similar influence of negative hyperconjugation for charge delocalization was found in superbasic PAPs^16^ and protonated diphosphazenes.^53^ The P–C–P angles in the bisprotonated forms (**4**: 120°, **1**: 113°, **2**: 121°) are more acute than in the free CDPs (**4**: 156°, **1**: 147°). The difference to ideal tetrahedral geometry presumably arise from the bulkiness of the PR_3_ moieties.

### NMR spectroscopic features

All six presented compounds were characterized by ^1^H, ^13^C, and ^31^P NMR spectroscopy. Selected chemical shifts and couplings are collected in Table 1. Proton shifts of bis- and monoprotonated CDPs lie around 3 ppm for CH_2_ and below 1 ppm for CH groups, both decreasing with increasing basicity of the parent CDP indicating less polarized C–H bonds. This shielding trend is not observed in the ^13^C NMR shifts of the carbon nuclei: the most basic CDP **1** exhibits a triplet at 9.5 ppm compared to –1.6 ppm (**4**) and –6.8 ppm ((dma)_6_-CDP).^29^ Surprisingly the ^13^C chemical shift for **1** is even higher than for its monoprotonated form (**1·HTFSI**: 9.3 ppm) contrasting the typical trend observed for other CDPs.^31^,^54^ The ^1^*J*_PC_ couplings drastically increase with step by step deprotonation indicating larger s-character of the ylidic P–C bonds. In the ^31^P NMR spectra signals for the monoprotonated forms lie between the bisprotonated at higher and the free CDPs at lower values and correlate with the group electronegativity of the phosphines ((dma)_6_-CDP: 27.72 ppm; (dma)_6_-CDP·HCl: 54.16 ppm).^29^ This is not exactly the case for the bisprotonated and free CDPs. The ^31^P NMR signals of all three forms of **2** are multiplets corresponding to an AA′XX′ spin system with ^2^*J*_PP_ and ^4^*J*_PP_ coupling (Fig. S22, S25, and S29 in the ESI†). **7** exhibits four individual signals in shape of two doublets of doublets for bridging phosphorus atoms and two doublets for terminal phosphorus atoms with the P^III^ atom being characteristically deshielded.^15^,^16^ ^1^H and ^13^C NMR signals are slightly shifted to higher frequencies in comparison with **2·HBF_4_**, indicating that the mixed valent P^III^/P^V^ phosphanylphosphazene substituent is a poorer donor than corresponding P_2_ bisphosphazene.

**Table 1:** NMR shifts *δ*/ppm and couplings *J*/Hz of the presented compounds

	*δ*_H_ (^2^*J*_PH_/^4^*J*_PH_)	*δ*_C_ (^1^*J*_PC_/^3^*J*_PC_)	*δ*_P_
**4·2HBF_4_** ^*a*^	3.43 (19)	26.4 (110)	32.7
**4·HTFSI** ^*b*^	0.93 (7)	10.3 (192)	40.1
**4** ^*c*^	—	–1.6 (280)	11.5
**1·2HBF_4_** ^*a*^	3.16 (17)	25.2 (112)	20.8
**1·HTFSI** ^*b*^	0.55 (4)	9.3 (185)	37.1
**1** ^*c*^	—	9.5 (209)	18.2
**2·2HBF_4_** ^*a*^	2.87 (19)	25.6 (122/7)	23.2–22.7, 20.6–20.3
**2·HBF_4_** ^*a*^	0.25 (6/3)	12.6 (194/4)	34.3–33.6, 16.5–15.8
**2** ^*d*^	—		7.7–7.0, 6.2–5.6
**7** ^*c*^	0.42 (3/2)	13.0 (187/186/2)	109.9, 39.9, 37.0, 15.1

^*a*^In CD_3_CN.

^*b*^In THF-*d*_8_.

^*c*^In C_6_D_6_.

^*d*^In C_6_D_6_, assigned from the isolated mixture of the reaction between **2·2HBF_4_** and KH in THF (Fig. S29 in the ESI).

NMR titration experiments were conducted for **4** against (tmg)P_1_-*t*Bu (p*K*_BH_^+^ in THF: 29.1)^6^ and (dma)P_4_-*t*Bu (p*K*_BH_^+^ in THF: 33.9).^20^ The p*K*_BH_^+^ value for **4** therefore has to be in between 30.1 and 32.9, since only free (tmg)P_1_-*t*Bu and protonated **4** or protonated (dma)P_4_-*t*Bu and free **4** were detected, respectively. Basicity of **1** was determined *via* titration against (pyrr)P_4_-*t*Bu (p*K*_BH_^+^ in THF: 35.3)^20^ as reference. Protonated and base forms of both species were quantified by ^31^P NMR integration and a p*K*_BH_^+^ value of 35.8 in THF was determined for **1**. To our knowledge this is the first report of an experimental p*K*_BH_^+^ value for a carbodiphosphorane. It approves **1** to be an exceptional strong non-ionic carbon base, 0.5 orders of magnitude more basic than the strongest uncharged Schwesinger-type nitrogen superbase measured in THF^20^ and 2.3 orders of magnitude more basic than the so far strongest uncharged carbon superbase H_2_C=P(2,4,6-(MeO)_3_–C_6_H_2_)_2_Ph (p*K*_BH_^+^ in THF: 33.5).^20^ Singlet carbenes such as NHCs and CAACs are weak carbon bases in comparison, according to p*K*_BH_^+^ values around 23 in THF and DMSO^55^ or calculated PAs.^34^,^56^ The exceptional C-basicity of the title compounds is only surpassed by our PAP phosphorus superbases (pyrr)P_3_P (36.7) and (dma)P_4_P (37.2).^16^

### Quantumchemical calculations

First and second proton affinity (PA) and gas-phase basicity (GB) of carbodiphosphoranes **1**, **2**, **4** and phosphine **7** are calculated utilizing M06-2X/6-11+G(2df,p)//M06-2X/6-31+G(d) theoretical model. p*K*_BH_^+^ values in THF are obtained using the same functional and basis set whereas solvent is treated as dielectric continuum utilizing the SMD solvation model. p*K*_BH_^+^ values are calculated as relative values using an isodesmic reaction approach^57^ where Schwesingers (dma)P_4_-*t*Bu phosphazene with p*K*_BH_^+^ of 33.9 (ref. 20) has served as a reference base. Calculated values for protonation at central carbon atom, and in case of **7** protonation at the P^III^ atom as well, are presented in Table 2. It appears that the first proton affinity as well as p*K*_BH_^+^ values of **1** and **2** are higher than in Schwesingers (dma)P_4_-*t*Bu phosphazene which has PA of 293.3 kcal mol^–1^ calculated at the same level of theory. Interestingly first GB of **1** is slightly lower than the GB of (dma)P_4_-*t*Bu (GB = 288.2 kcal mol^–1^) implying that the higher p*K*_BH_^+^ value of **1** relative to (dma)P_4_-*t*Bu is a result of a more pronounced solvation effect in the carbodiphosphorane. This is unexpected considering that the N–H bond in a protonated phosphazene has a higher polarity than the C–H bond in protonated CDP as a result of lower electronegativity of carbon relative to nitrogen. The calculated p*K*_BH_^+^ (THF) 39.1 of **2** would be far higher than the p*K*_BH_^+^ (THF) 33.9 of (dma)P_4_-*t*Bu, the strongest commercially available superbase. As described isolation of neutral base **2** is not achieved experimentally as other C–H bonds in the precursor **2·H^+^** seemed to have a higher kinetic and thermodynamic acidity. In order to understand the deprotonation path of **2·H^+^** under the action of NaNH_2_, the reaction profile is calculated and presented in Fig. S36 in the ESI.† It appears, that the deprotonation of peripheral NMe_2_ group in combination with the irreversible elimination of *N*-methylmethanimine is thermodynamically feasible (exergonic), however, kinetically hindered by a high barrier (Δ*G*^‡^ = 32.8 kcal mol^–1^). This explains, that deprotonation induced degradation is competitive to deprotonation of central carbon atom at elevated temperatures, though the central carbon atom in **2·H^+^** is the thermodynamically most acidic site. It appears that decomposition product – phosphine **7** – has a gas-phase basicity (30.9 kcal mol^–1^) much lower than CDP **2**. Interestingly, GB value for protonation at central carbon and P^III^ phosphorus of **7** is almost the same, whereas p*K*_BH_^+^ in THF for protonation at P^III^ is by 3.3 orders of magnitude lower than p*K*_BH_^+^ for protonation at carbon, which again indicates a more pronounced solvation effect in C-protonated CDP.

**Table 2:** Calculated first and second proton affinity (PA) and gas phase basicity (GB) together with p*K*_BH_^+^ values in THF

		PA/kcal mol^–1^	GB/kcal mol^–1^	p*K*_BH_^+^ in THF^*a*^
**4**	1^st^	291.1	282.2	32.8 (30.1–32.9)
	2^nd^	191.6	184.0	—
**1**	1^st^	294.4	287.2	34.9 (35.8 ± 1)
	2^nd^	202.0	194.1	—
**2**	1^st^	305.3	299.7	39.1
	2^nd^	212.1	202.2	—
**7**	At carbon	275.9	268.7	24.4
	At phosphorus	276.2	268.8	21.1

^*a*^Experimental values in parentheses.

## Conclusions

In this work we presented the most basic uncharged carbon bases known so far. A convenient synthesis for first- and novel second-order carbodiphosphorane superbases was presented. The CDPs (pyrr)_6_-CDP **4** and *sym*-(tmg)_2_(dma)_4_-CDP **1** were synthesized as free base as well as in their mono- and bisprotonated forms. In our attempt to synthesize the even more outstanding base *sym*-(dmaP_1_)_2_(dma)_4_-CDP **2** an unexpected, but highly selective deprotonation at peripheral PNCH_3_ bonds induced an irreversible elimination path towards phosphine **7**. This reaction is indicating a potential basicity limit for phosphazene containing superbases. Structural as well as spectroscopic features were investigated and the basicity was quantified by theoretical and experimental means. Remarkable p*K*_BH_^+^ values for **4** and **1** confirm them as benchmark breakers for non-ionic carbon bases on the THF basicity scale. Compared to the top Schwesinger bases, this basicity is even more outstanding, if their molecular weight below 500 g mol^–1^ is considered. We expect, that such simply synthesized carbodiphosphoranes with water stable protonated forms will enter the field of organic superbase catalysis.^1^

## Experimental section

### General

All Reactions with air or moisture sensitive substances were carried out under inert atmosphere using standard Schlenk techniques. Air or moisture sensitive substances were stored in a nitrogen-flushed glovebox. Solvents were purified according to common literature procedures and stored under an inert atmosphere over molsieve (3 Å or 4 Å).^58^ Pyrrolidine and tetramethylguanidine were distilled from CaH_2_, triflimidic acid was purified by sublimation under argon. Bis(dichlorophosphino)methane^38^ (**6**), bis[bis(dimethylamino)phosphino]methane^38^ (**3**), tris(dimethylamino)phosphazene^4^ and (pyrr)P_4_-*t*Bu^4^ were prepared according to literature-known procedures. (dma)P_4_-*t*Bu was purchased as 1 M solution in *n*-hexane and dried in high vacuum. All other reagents were used as provided.

^1^H, ^13^C, and ^31^P NMR spectra were recorded on a Bruker Avance III HD 250, Avance II 300, Avance III HD 300 or Avance III HD 500 spectrometer. Chemical shift *δ* is denoted relatively to SiMe_4_ (^1^H, ^13^C) or 85% H_3_PO_4_ (^31^P). ^1^H and ^13^C NMR spectra were referenced to the solvent signals.^59^ Multiplicity is abbreviated as follows: s (singlet), d (doublet), t (triplet), q (quartet), m (multiplet), br. (broad signal). High resolution mass spectrometry were performed on a Thermo Fisher Scientific LTQ-FT Ultra (ESI(+)) or a Jeol AccuTOF GCv (LIFDI(+) = liquid injection field desorption ionization), elemental analysis on an Elementar Vario Micro Cube. IR spectra were recorded in a glovebox on a Bruker Alpha ATR-FT-IR. CCDC ; 1903830 (**4·2HCl + HpyrrCl**), ; 1903833 (**1·2HBF_4_**), ; 1903838 (**2·2HBF_4_**), ; 1903840 (**1**), ; 1903841 (**4·2HBF_4_**), and ; 1903843 (**4**) contain the supplementary crystallographic data for this paper.†

### General procedure for the precipitation of BF_4_-salts

The crude product was dissolved in a minimum amount of water and a concentrated aqueous sodium tetrafluoridoborate solution (2.0 eq.) was added. The resulting precipitate was filtered off, rinsed three times with small portions of cold water, washed with THF and dried in high vacuum.

### (pyrr)_6_-CDP·2HBF_4_ (**4·2HBF_4_**)

**6** (3.60 g, 16.5 mmol, 1.00 eq.) was dissolved in THF (60 mL), cooled to –78 °C and pyrrolidine (17.7 mL, 216 mmol, 13.1 eq.) was added dropwise. Afterwards the cooling bath was removed and the mixture stirred for additional 6 h. Carbon tetrachloride (3.12 mL, 32.3 mmol, 1.96 eq.) was added at –78 °C and the mixture allowed to warm to room temperature overnight. The suspension was filtered under air and the filter cake extracted with THF (3 × 60 mL). The solvent was removed under reduced pressure and the residue dried in high vacuum. The crude product was converted to its tetrafluoridoborate salt as described in the general procedure and recrystallized from methanol/ethanol. **4·2HBF_4_** (6.38 g, 9.52 mmol, 58%) was obtained as colourless solid.

[C_25_H_50_B_2_F_8_N_6_P_2_] (670.27 g mol^–1^) ^1^H NMR (500.2 MHz, CD_3_CN): *δ* (ppm) = 3.43 (t, ^2^*J*_PH_ = 19 Hz, 2H, C*H*_2_), 3.25–3.22 (m, 24H, *H*1), 1.97–1.95 (m, 24H, *H*2, (overlapped with the solvent signal)). ^13^C{^1^H} NMR (125.8 MHz, CD_3_CN): *δ* (ppm) = 48.7 (s, *C*1), 26.9–26.8 (m, *C*2), 26.4 (t, ^1^*J*_PC_ = 110 Hz, *C*H_2_). ^31^P{^1^H} NMR (121.5 MHz, CD_3_CN): *δ* (ppm) = 32.7. ESI(+) MS (MeOH): *m*/*z* (%) = 495.6 (100) [M – H – 2BF_4_]^+^, 583.2 (5) [M – BF_4_]^+^. ESI(+) HRMS: *m*/*z* [M – H – 2BF_4_]^+^ calcd 495.3488, found 495.3505; [M – BF_4_]^+^ calcd 583.3600, found 583.3611. Elemental analysis: calcd C 44.80%, H 7.52%, N 12.54%; found C 44.49%, H 7.50%, N 12.46%. IR (neat): *ν̃* (cm^–1^) = 2970 (w), 2879 (w), 1458 (w), 1251 (w), 1210 (m), 1134 (m), 1047 (*vs.*), 1021 (*vs.*), 918 (m), 870 (m), 824 (m), 779 (m), 699 (m), 581 (w), 549 (w), 517 (m) 484 (s). XRD: for single crystal X-ray structure determination suitable single crystals were obtained by slow evaporation of a concentrated solution in chloroform.

### *sym*-(tmg)_2_(dma)_4_-CDP·2HBF_4_ (**1·2HBF_4_**)

**3** (831 mg, 3.29 mmol, 1.00 eq.) and tetramethylguanidine (1.14 g, 9.88 mmol, 3.00 eq.) were dissolved in THF (60 mL). Carbon tetrachloride (640 μL, 6.62 mmol, 2.01 eq.) was added at –78 °C and the mixture allowed to warm to room temperature overnight. The suspension was filtered under air and the filter cake extracted with THF (3 × 20 mL). The solvent was removed under reduced pressure and the residue dried in high vacuum. The crude product was converted to its tetrafluoridoborate salt as described in the general procedure and recrystallized from ethanol. **1·2HBF_4_** (1.08 g, 1.66 mmol, 50%) was isolated as colourless solid.

[C_10_H_50_B_2_F_8_N_10_P_2_] (654.24 g mol^–1^) ^1^H NMR (500.2 MHz, CD_3_CN): *δ* (ppm) = 3.16 (t, ^2^*J*_PH_ = 17 Hz, 2H, C*H*_2_), 2.91 (s, 24H, *H*1), 25.3 (d, ^3^*J*_PH_ = 10 Hz, 24H, *H*2). ^13^C{^1^H} NMR (125.8 MHz, CD_3_CN): *δ* (ppm) = 161.6 (dd, 2× ^2,4^*J*_PC_ = 2 Hz, *C*N_3_), 40.9 (s, *C*1), 37.1 (dd, 2× ^2,4^*J*_PC_ = 2 Hz, *C*2), 25.2 (t, ^1^*J*_PC_ = 112 Hz, *C*H_2_). ^31^P{^1^H} NMR (202.5 MHz, CD_3_CN): *δ* (ppm) = 20.8 (s, ^1^*J*_PC_ = 113 Hz (satellites)). ESI(+) MS (MeOH): *m*/*z* (%) = 479.5 (100) [M – H – 2BF_4_]^+^. ESI(+) HRMS: *m*/*z* [M – H – 2BF_4_]^+^ calcd. 479.3622, found 479.3625. Elemental analysis: calcd C 34.88%, H 7.70%, N 21.41%; found C 34.98%, H 7.84%, N 21.39%. IR (neat): *ν̃* (cm^–1^) = 2911 (br. w.), 1539 (s), 1486 (m), 1429 (m), 1401 (m), 1356 (m), 1289 (m), 1235 (w), 1186 (m), 1161 (m), 1046 (*vs.*), 1034 (*vs.*), 979 (*vs.*), 933 (*vs.*), 784 (s), 771 (s), 739 (m), 716 (m), 690 (w), 672 (w), 618 (w), 572 (m), 519 (m), 459 (m), 437 (m). XRD: for single crystal X-ray structure determination suitable single crystals were obtained from ethanol at –25 °C.

### *sym*-(dmaP_1_)_2_(dma)_4_-CDP·2HBF_4_ (**2·2HBF_4_**)

**3** (1.55 g, 6.14 mmol, 1.00 eq.) and tris(dimethylamino)phosphazene (3.28 g, 18.4 mmol, 3.00 eq.) were dissolved in THF (60 mL). Carbon tetrachloride (1.19 mL, 12.3 mmol, 2.00 eq.) was added at –78 °C and the mixture allowed to warm to room temperature overnight. The suspension was filtered under air and the filter cake extracted with THF (3 × 20 mL). The solvent was removed under reduced pressure and the residue dried in high vacuum. The crude product was converted to its tetrafluoridoborate salt as described in the general procedure and recrystallized from ethanol/*n*-hexane. **2·2HBF_4_** (2.58 g, 3.31 mmol, 54%) was isolated as colourless solid.

[C_21_H_62_B_2_F_8_N_12_P_4_] (780.31 g mol^–1^) ^1^H NMR (500.2 MHz, CD_3_CN): *δ* (ppm) = 2.87 (t, ^2^*J*_PH_ = 19 Hz, 2H, C*H*_2_), 2.68 (d, ^3^*J*_PH_ = 11 Hz, 24H, *H*2), 2.65 (d, ^3^*J*_PH_ = 10 Hz, 36H, *H*1). ^13^C{^1^H} NMR (125.8 MHz, CD_3_CN): *δ* (ppm) = 37.3 (m, *C*1, *C*2), 25.6 (tt, ^1^*J*_PC_ = 122 Hz, ^3^*J*_PC_ = 7 Hz, *C*H_2_). ^31^P{^1^H} NMR (202.5 MHz, CD_3_CN): *δ* (ppm) = 23.2–22.7 (m, *P*1), 20.6–20.3 (m, *P*2). ESI(+) MS (MeOH): *m*/*z* (%) = 303.5 (25) [M – 2BF_4_]^2+^, 605.6 (60) [M – H – 2BF_4_]^+^, 693.5 (100) [M – BF_4_]^+^. ESI(+) HRMS: *m*/*z* [M – 2BF_4_]^2+^ calcd 303.2080, found 303.2088; [M – H – 2BF_4_]^+^ calcd 605.4087, found 605.4104; [M – BF_4_]^+^ calcd 693.4195, found 693.4215. Elemental analysis: calcd C 32.32%, H 8.01%, N 21.54%; found C 31.94%, H 7.70%, N 21.18%. IR (neat): *ν̃* (cm^–1^) = 2886 (w), 1539 (s), 1486 (m), 1429 (m), 1401 (m), 1356 (m), 1298 (m), 1234 (m), 1186 (w), 1161 (m), 1047 (*vs.*), 1035 (*vs.*), 979 (*vs.*), 933 (s), 784 (s), 771 (s), 739 (m), 715 (m), 690 (m), 672 (m), 572 (m), 519 (m), 459 (m), 439 (m). XRD: for single crystal X-ray structure determination suitable single crystals were obtained from ethanol/*n*-hexane at –25 °C.

### (pyrr)_6_-CDP (**4**)

A solution of potassium bis(trimethylsilyl)amide (558 mg, 2.80 mmol, 2.09 eq.) in THF (15 mL) was added to a suspension of **4·2HBF_4_** (938 mg, 1.34 mmol, 1.00 eq.) in THF (40 mL) and stirred for 16 h at room temperature. All volatiles were removed *in vacuo*, the residue dissolved in *n*-hexane (20 mL) and filtered over Celite. The filter cake was extracted with *n*-hexane (2 × 15 mL) and the filtrate evaporated to dryness. **4** (481 mg, 973 μmol, 70%) was isolated as colourless solid. [C_25_H_48_N_6_P_2_] (494.65 g mol^–1^) ^1^H NMR (500.2 MHz, C_6_D_6_): *δ* (ppm) = 3.33–3.23 (m, 24H, *H*1), 1.75–1.64 (m, 24H, *H*2). ^13^C{^1^H} NMR (125.8 MHz, C_6_D_6_): *δ* (ppm) = 47.4 (s, *C*1), 28.9 (s, *C*2), –1.6 (t, ^1^*J*_PC_ = 280 Hz, P*C*P). ^31^P{^1^H}-NMR (202.5 MHz, C_6_D_6_): *δ* (ppm) = 11.5. LIFDI(+) MS (*n*-hexane): *m*/*z* (%) = 495.4 (100) [M + H]^+^. LIFDI(+) HRMS: *m*/*z* [M + H]^+^ calcd 495.34939, found 495.35037. Elemental analysis: calcd C 60.70%, H 9.78%, N 16.99%; found C 60.39%, H 9.62%, N 17.42%. IR (neat): *ν̃* (cm^–1^) = 2952 (m), 2836 (m), 1492 (w), 1435 (s), 1338 (m), 1319 (m), 1289 (w), 1191 (m), 1134 (m), 1046 (*vs.*), 1000 (*vs.*), 980 (*vs.*), 909 (s), 870 (m), 742 (m), 546 (*vs.*), 497 (*vs.*). XRD: for single crystal X-ray structure determination suitable single crystals were obtained from *n*-hexane at –25 °C.

### *sym*-(tmg)_2_(dma)_4_-CDP (**1**)

A mixture of **1·2HBF_4_** (190 mg, 290 μmol, 1.00 eq.) and sodium amide (113 mg, 2.90 mmol, 10.0 eq.) was stirred in THF (15 mL) for 16 h at room temperature. The suspension was filtered over Celite and the filter cake extracted with THF (3 × 5 mL). All volatiles were removed *in vacuo*, *n*-hexane (10 mL) added to the residue, filtered again over Celite and extracted with *n*-hexane (3 × 4 mL). Evaporation of the solvent and drying in high vacuum yielded **1** (86 mg, 0.17 mmol, 60%) as colourless solid. [C_19_H_48_N_10_P_2_] (478.61 g mol^–1^) ^1^H NMR (500.2 MHz, C_6_D_6_): *δ* (ppm) = 2.88 (dd, 2× ^3,5^*J*_PH_ = 5 Hz, 24H, *H*2), 2.73 (s, 24H, *H*1). ^13^C{^1^H} NMR (125.8 MHz, C_6_D_6_): *δ* (ppm) = 156.0 (s, *C*N_3_), 40.1 (s, *C*1), 38.3 (s, *C*2), 9.5 (t, ^1^*J*_PC_ = 209 Hz, P*C*P). ^31^P{^1^H} NMR (121.5 MHz, C_6_D_6_): *δ* (ppm) = 18.2. LIFDI(+) MS (*n*-hexane): *m*/*z* (%) = 479.4 (100) [M + H]^+^. LIFDI(+) HRMS: *m*/*z* [M + H]^+^ calcd 479.36169, found 479.36229. Elemental analysis: calcd C 47.68%, H 10.11%, N 29.27%; found C 47.54%, H 9.96%, N 29.47%. IR (neat): *ν̃* (cm^–1^) = 3006 (w), 2847 (m), 2810 (m), 2778 (m), 1566 (*vs.*), 1496 (s), 1472 (m), 1453 (m), 1440 (m), 1421 (m), 1358 (*vs.*), 1281 (m), 1251 (m), 1235 (m), 1211 (m), 1173 (m), 1128 (s), 1052 (m), 971 (s), 949 (*vs.*), 917 (m), 860 (*vs.*), 796 (m), 748 (m), 685 (s), 652 (s), 629 (*vs.*), 568 (m), 527 (s), 452 (s). XRD: for single crystal X-ray structure determination suitable single crystals were obtained from *n*-hexane at –25 °C.

### Attempted synthesis of *sym*-(dmaP_1_)_2_(dma)_4_-CDP (**2**)

A mixture of **2·2HBF_4_** (136 mg, 174 μmol, 1.0 eq.) and freshly ground sodium amide (75 mg, 1.9 mmol, 11 eq.) was suspended in THF (15 mL) and stirred for 72 h at 60 °C. The solid was removed by filtration over Celite and the filtrate evaporated to dryness. The residue was dissolved in *n*-pentane (20 mL), cleared *via* syringe filtration, the solvent removed and the residue dried in high vacuum to give **7** as pale yellow high viscous oil.

[C_19_H_55_N_10_P_4_] (561.62 g mol^–1^) ^1^H NMR (300.3 MHz, C_6_D_6_): *δ* (ppm) = 2.99 (d, ^3^*J*_PH_ = 9 Hz, 12H, *H*4), 2.88 (d, ^3^*J*_PH_ = 10 Hz, 12H, *H*3), 2.83 (d, ^3^*J*_PH_ = 11 Hz, 12H, *H*2), 2.32 (d, ^3^*J*_PH_ = 10 Hz, 18H, *H*1), 0.42 (dddd, 2× ^2^*J*_PH_ = 3 Hz, 2× ^4^*J*_PH_ = 2 Hz, 1H, C*H*). ^13^C{^1^H} NMR (75.5 MHz, C_6_D_6_): *δ* (ppm) = 38.5 (dd, ^2^*J*_PC_ = 4 Hz, ^4^*J*_PC_ = 3 Hz, *C*3), 38.4 (d, ^2^*J*_PC_ = 16 Hz, *C*4), 38.1 (dd, ^2^*J*_PC_ = 4 Hz, ^4^*J*_PC_ = 1 Hz, *C*2) 37.1 (d, ^2^*J*_PC_ = 4 Hz, *C*1), 13.0 (ddd, ^1^*J*_PC_ = 187 Hz, ^1^*J*_PC_ = 186 Hz, ^3^*J*_PC_ = 2 Hz, *C*H). ^31^P{^1^H} NMR (121.5 MHz, C_6_D_6_): *δ* (ppm) = 109.9 (d, ^2^*J*_PP_ = 100 Hz, *P*4), 39.9 (dd, ^2^*J*_PP_ = 50 Hz, ^2^*J*_PP_ = 41 Hz, *P*2), 37.0 (dd, ^2^*J*_PP_ = 100 Hz, ^2^*J*_PP_ = 41 Hz, *P*3), 15.1 (d, ^2^*J*_PP_ = 50 Hz, *P*1). LIFDI(+) MS (*n*-hexane): *m*/*z* (%) = 561.4 (100) [M]^+^. LIFDI(+) HRMS: *m*/*z* [M]^+^ calcd 561.35924, found 561.35562.

### (pyrr)_6_-CDP·HTFSI (**4·HTFSI**)

**4** (8.954 mg, 18.10 μmol, 1.04 eq.) and triflimidic acid (4.911 mg, 17.46 μmol, 1.00 eq.) were mixed in THF-*d*_8_ (0.5 mL) and used for analytics.

[C_27_H_49_F_6_N_7_O_4_P_2_S_2_] (775.79 g mol^–1^) ^1^H NMR (500.2 MHz, THF-*d*_8_): *δ* (ppm) = 3.20–3.17 (m, 24H, *H*1), 1.88–1.85 (m, 24H, *H*2), 0.93 (t, ^2^*J*_PH_ = 7 Hz, 1H, C*H*). ^13^C{^1^H} NMR (125.8 MHz, THF-*d*_8_): *δ* (ppm) = 121.1 (q, ^1^*J*_FC_ = 323 Hz, *C*F_3_), 47.8 (s, *C*1), 26.9 (dd, 2× *J*_PC_ = 4 Hz, *C*2), 10.3 (t, ^1^*J*_PC_ = 192 Hz, *C*H). ^31^P{^1^H} NMR (121.5 MHz, THF-*d*_8_): *δ* (ppm) = 40.1. LIFDI(+) MS (THF): *m*/*z* (%) = 495.4 (100) [M – TFSI]^+^. LIFDI(+) HRMS: *m*/*z* [M – TFSI]^+^ calcd 495.34939, found 495.35146.

### *sym*-(tmg)_2_(dma)_4_-CDP·HTFSI (**1·HTFSI**)

**1** (9.273 mg, 19.38 μmol, 1.00 eq.) and triflimidic acid (5.517 mg, 19.62 μmol, 1.01 eq.) were mixed in THF-*d*_8_ (0.5 mL) and used for analytics.

[C_21_H_49_F_6_N_11_O_4_P_2_S_2_] (759.75 g mol^–1^) ^1^H NMR (300.3 MHz, THF-*d*_8_): *δ* (ppm) = 2.90 (s, 24H, *H*1), 2.67–2.64 (m, 24H, *H*2), 0.55 (t, ^2^*J*_PH_ = 4 Hz, 1H, C*H*). ^13^C{^1^H} NMR (75.5 MHz, THF-*d*_8_): *δ* (ppm) = 161.1 (s, *C*N_3_), 121.1 (q, ^1^*J*_FC_ = 322 Hz, *C*F_3_), 40.3 (s, *C*1), 37.7 (dd, 2× ^2,4^*J*_PC_ = 2 Hz, *C*2), 9.3 (t, ^1^*J*_PC_ = 185 Hz, *C*H). ^31^P{^1^H} NMR (121.5 MHz, C_6_D_6_): *δ* (ppm) = 37.1. LIFDI(+) MS (THF): *m*/*z* (%) = 479.4 (100) [M – TFSI]^+^. LIFDI(+) HRMS: *m*/*z* [M – TFSI]^+^ calcd 479.36169, found 479.36232.

### *sym*-(dmaP_1_)_2_(dma)_4_-CDP·HBF_4_ (**2·HBF_4_**)

A mixture of **2·2HBF_4_** (600 mg, 769 μmol, 1.00 eq.) and finely ground sodium amide (321 mg, 8.23 mmol, 10.7 eq.) was suspended in THF (20 mL), cooled to –78 °C and ammonia (*ca.* 40 mL) was condensed in. The mixture was allowed to warm to room temperature overnight, the solid removed by centrifugation and the supernatant evaporated to dryness. The residue was dissolved in dichloromethane (40 mL) and filtered over Celite. All volatiles were removed *in vacuo*, the residue washed with diethyl ether (2 × 40 mL) and dried in high vacuum. **2·HBF_4_** (365 mg, 527 μmol, 69%) was isolated as colorless solid. [C_21_H_61_BF_4_N_10_P_4_] (692.50 g mol^–1^) ^1^H NMR (500.2 MHz, CD_3_CN): *δ* (ppm) = 2.64 (d, ^3^*J*_PH_ = 10 Hz, 36H, *H*1), 2.60–2.57 (m, 24H, *H*2), 0.25 (tt, ^2^*J*_PH_ = 6 Hz, ^4^*J*_PH_ = 3 Hz, 1H, C*H*). ^13^C{^1^H} NMR (125.8 MHz, CD_3_CN): *δ* (ppm) = 37.9 (d, ^2^*J*_PC_ = 2 Hz, *C*2), 37.4 (d, ^2^*J*_PC_ = 5 Hz, *C*1), 12.6 (tt, ^1^*J*_PC_ = 194 Hz, ^3^*J*_PC_ = 4 Hz, *C*H). ^31^P{^1^H} NMR (121.5 MHz, CD_3_CN): *δ* (ppm) = 34.3–33.6 (m, *P*2), 16.5–15.8 (m, *P*1). LIFDI(+) MS (THF): *m*/*z* (%) = 605.4 (100) [M – BF_4_]^+^. LIFDI(+) HRMS: *m*/*z* [M – BF_4_]^+^ calcd 605.40926, found 605.41147. Elemental analysis: calcd C 36.42%, H 8.88%, N 24.27%; found C 36.25%, H 8.59%, N 24.21%. IR (neat): *ν̃* (cm^–1^) = 3000 (w), 2883 (m), 2846 (m), 2804 (m), 1458 (m), 1288 (s), 1243 (m), 1183 (m), 1167 (m), 1092 (m), 1048 (s), 976 (*vs.*), 955 (*vs.*), 845 (m), 823 (m), 770 (m), 740 (s), 715 (s), 660 (s), 598 (m), 551 (w), 527 (m), 498 (s), 454 (m), 420 (w).

## Conflicts of interest

The authors have no conflicts to declare.

## Supplementary Material

Supplementary information
