# Supplementary material for: Design of non-ionic carbon superbases: second generation carbodiphosphoranes
Source: Chem Sci. 2019 Aug 16;10(41):9483–92. doi: 10.1039/c9sc03565f (PMC6993619; doi:10.1039/c9sc03565f)
Supplement: Supplementary file 1 [file SC-010-C9SC03565F-s001.pdf]

Electronic Supplementary Material (ESI) for Chemical Science.
This journal is © The Royal Society of Chemistry 2019

Design of Non-Ionic Carbon Superbases: Second Generation of Carbodiphosphoranes

Sebastian Ullrich,[a] Borislav Kovačević,[b] Björn Koch,[a] Klaus Harms[a] and Jörg Sundermeyer*[a]

[a]Fachbereich Chemie, Philipps-Universität Marburg, Hans-Meerwein-Straße, 35032 Marburg, Germany.

[b]The Group for Computational Life Sciences, Rudjer Bošković Institute, Bijenička c. 54, HR-10000 Zagreb, Croatia.

*E-mail: jsu@staff.uni-marburg.de

Supporting Information

Table of Content

NMR Titration Experiments S1

NMR Spectra S3

Crystallographic Section S20

Computational Section S24

References S48

NMR titration experiments

The experimenal p$K_{BH}$$^+$ values in THF of **1** and **4** were determined via NMR titration. The general procedure for NMR titration experiments for the determination of p$K_{BH}$$^+$ values was described elsewhere.[1] The carbodiphosphorane (CDP) in its free form was mixed with a similar amount of a reference superbase in its protonated form ((tmg)P_1-*t*Bu·HBF_4, pK_a in THF: 29.1)[2] or with similar amounts of a reference base ((dma)P_4-*t*Bu, p$K_{BH}$$^+$ in THF: 33.9; (pyrr)P_4-*t*Bu, p$K_{BH}$$^+$ in THF: 35.3)[2] and triflimidic acid (HTFSI) in THF-d_8. An equilibrium in competition of protons in solution was quickly reached. Quantitative ^{31}P{^{1}H} NMR spectra were recorded by inverse gated decoupling method with a relaxation delay of 25 s. Since proton exchange between the free CDP and its conjugate acid is slow on NMR timescale, neat signals were observed and used to determine the molar ratio of the different species at equilibrium. On the bases of these signal intensities equilibrium constants were thus calculated and the unknown p$K_{BH}$$^+$ values determined.

Sample A: **4** (5.281 mg, 10.68 µmol, 1.00eq), (dma)P_4-*t*Bu (7.101 mg, 11.20 µmol, 1.05 eq) and HTFSI (3.415 mg, 12.15 µmol, 1.14 eq) were mixed in THF-d_8 (0.6 mL).

Sample B: **4** (5.986 mg, 12.10 µmol, 1.00eq) and (tmg)P_1-*t*Bu·HBF_4 (6.659 mg, 12.05 µmol, 1.00 eq) were mixed in THF-d_8 (0.6 mL).

The $^{31}P\{^1H\}$ NMR spectra of the titration experiment in THF-d_8 are given in Figures S30-S32. In case of (tmg)P_1-*t*Bu as reference base, **4** deprotonated the used (tmg)P_1-*t*Bu·HBF_4 quantitatively, indicating a pK_{BH^+} value at least one order of magnitude higher than 29.1. In case of (dma)P_4-*t*Bu only the reference base was protonated by HTFSI with **4** remaining quantitativly in its free base form, indicating a pK_{BH^+} value one order of magnitude lower than 33.9. The pK_{BH^+} value of **4** can therefore be assigned between 30.1 and 32.9.

Sample C: **1** (3.423 mg, 7.152 µmol, 1.01 eq), (dma)P_4-*t*Bu (4.640 mg, 7.320 µmol, 1.03 eq) and HTFSI (1.997 mg, 7.103 µmol, 1.00 eq) were mixed in THF-d_8 (0.6 mL).

Sample D: **1** (7.772 mg, 16.24 µmol, 1.02 eq), (pyrr)P_4-*t*Bu (14.166 mg, 16.32 µmol, 1.03 eq) and HTFSI (4.470 mg, 15.90 µmol, 1.00 eq) were mixed in THF-d_8 (0.6 mL).

The $^{31}P\{^1H\}$ NMR spectra of the titration experiment in THF-d_8 are given in Figures S33-S35. In case of (dma)P_4-*t*Bu as reference base, only **1** was protonated by HTFSI with (dma)P_4-*t*Bu remaining quantitativly in its free base form, indicating a pK_{BH^+} value of **1** at least one order of magnitude higher than 33.9. In case of (pyrr)P_4-*t*Bu as reference base, signals for **1**, **1·HTFSI**, (pyrr)P_4-*t*Bu and (pyrr)P_4-*t*Bu·HTFSI were detected in the $^{31}P\{^1H\}$ NMR spectrum. Results of thermal dynamic basicity determination are shown in Table S1. Thus, the pK_{BH^+} of **1** was determined to be 35.8±1 in THF.

Table S1: $^{31}P\{^1H\}$ NMR titration experiments between **1** and (pyrr)P_4-*t*Bu with HTFSI in THF-d_8.

	**1**	(pyrr)P_4-*t*Bu	**1·H⁺**	(pyrr)P_4-*t*Bu·H^+
Initial weight/mg	7.772	14.166	0.00	0.00
Initial amount/µmol	16.24	16.32	0.00	0.00
Final amount/µmol	5.65	10.64	10.59	5.68
pK_{BH^+} (**1**) = pK_{BH^+} ((pyrr)P_4-*t*Bu) – log K = 35.3 – log [(5.65·5.68)÷(10.64·10.59)] = 35.8				

NMR Spectra

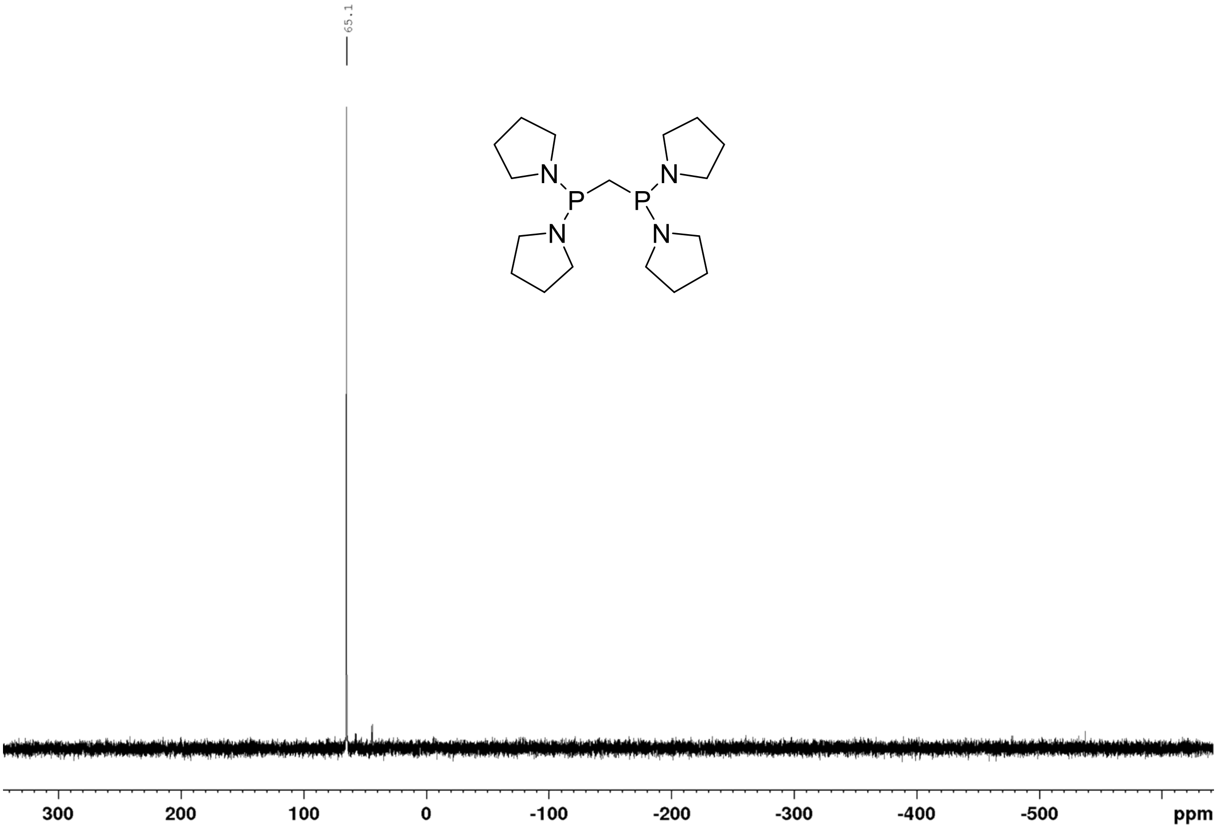

Figure S1: $^{31}P\{^{1}H\}$ NMR reaction control of in situ generated **5** (THF, 300 K, 101.3 MHz).

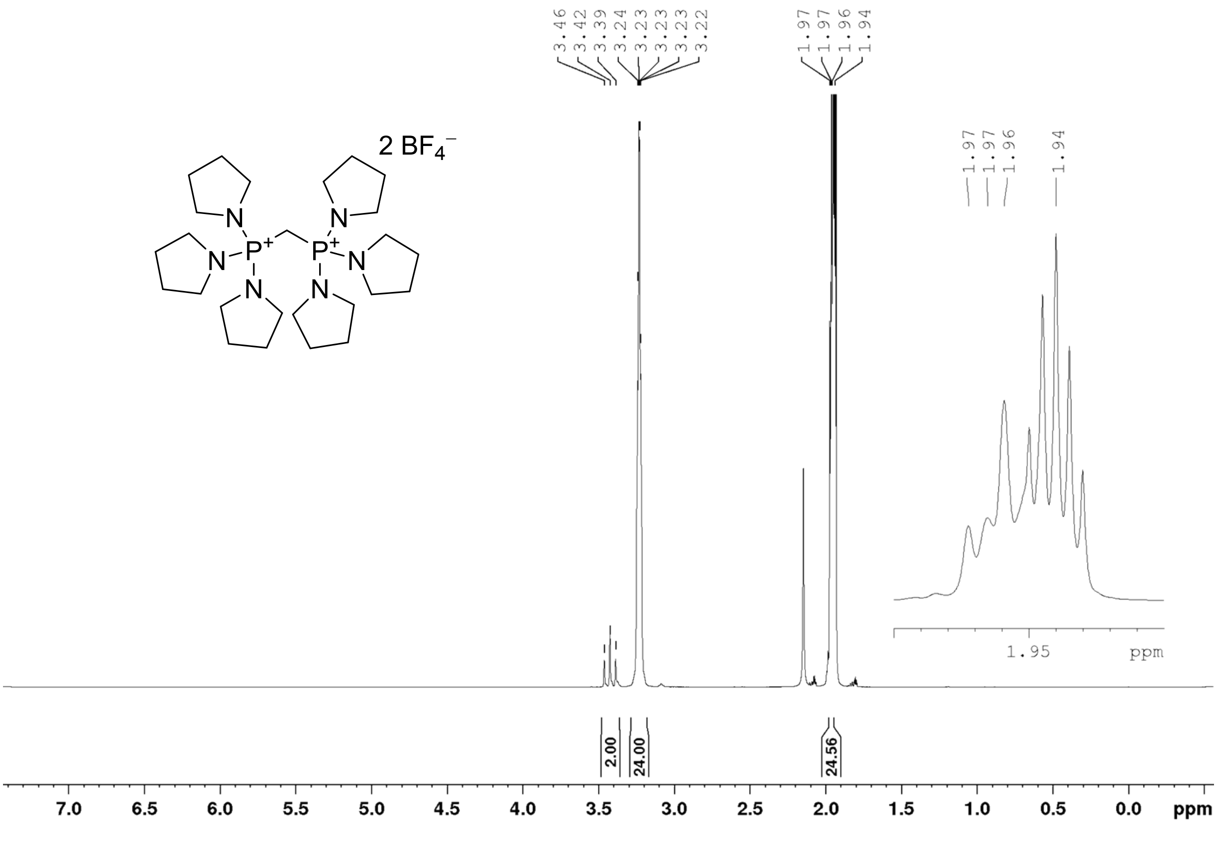

Figure S2: ^{1}H NMR spectrum of **4·2HBF$_4$** (CD_3CN, 300 K, 500.2 MHz).

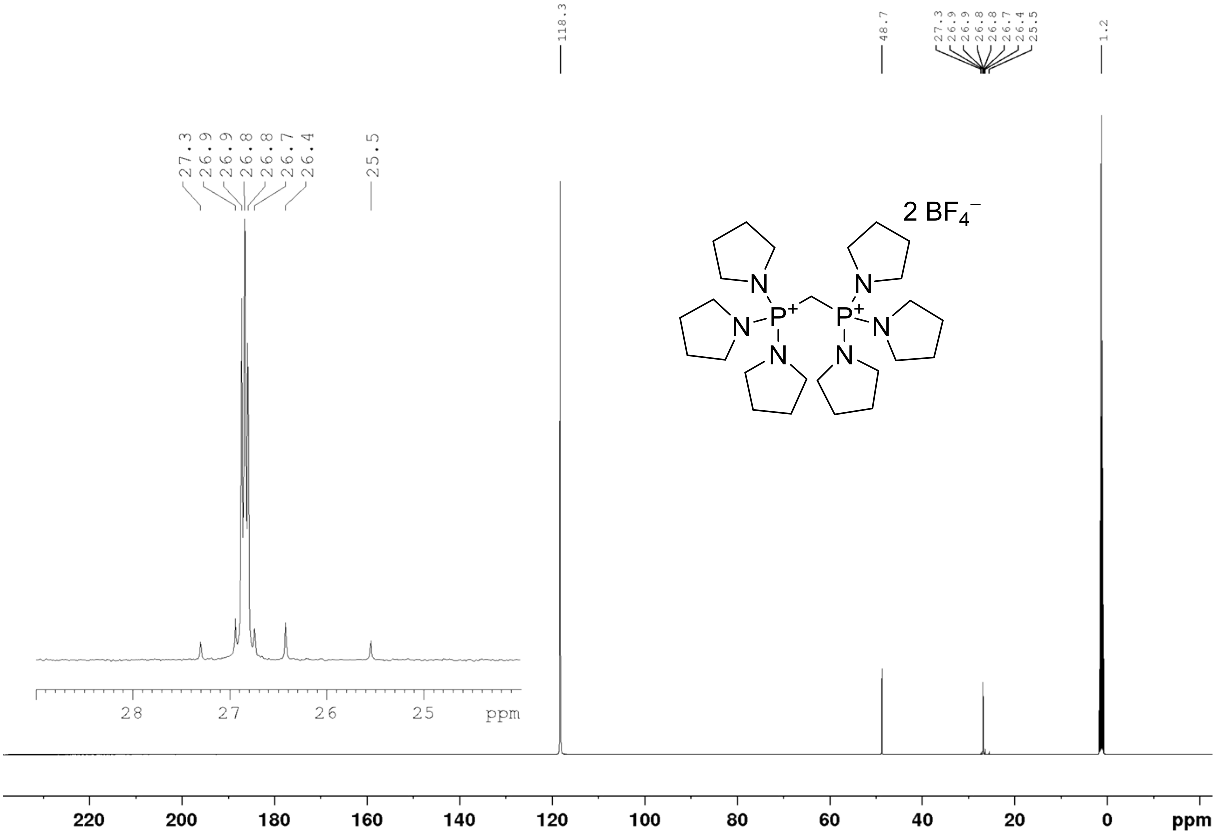

Figure S3: $^{13}C\{^1H\}$ NMR spectrum of **4·2HBF$_4$** (CD_3CN, 300 K, 125.8 MHz).

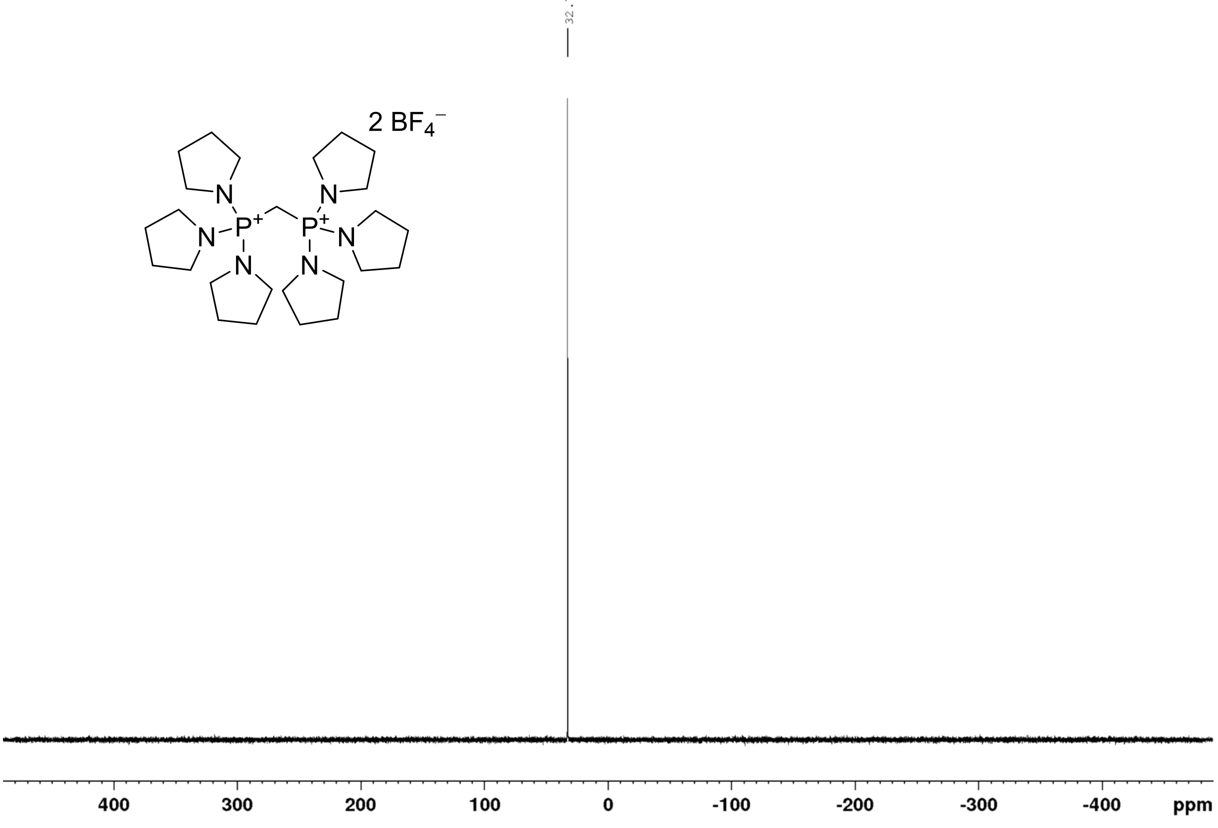

Figure S4: $^{31}P\{^1H\}$ NMR spectrum of **4·2HBF$_4$** (CD_3CN, 300 K, 121.5 MHz).

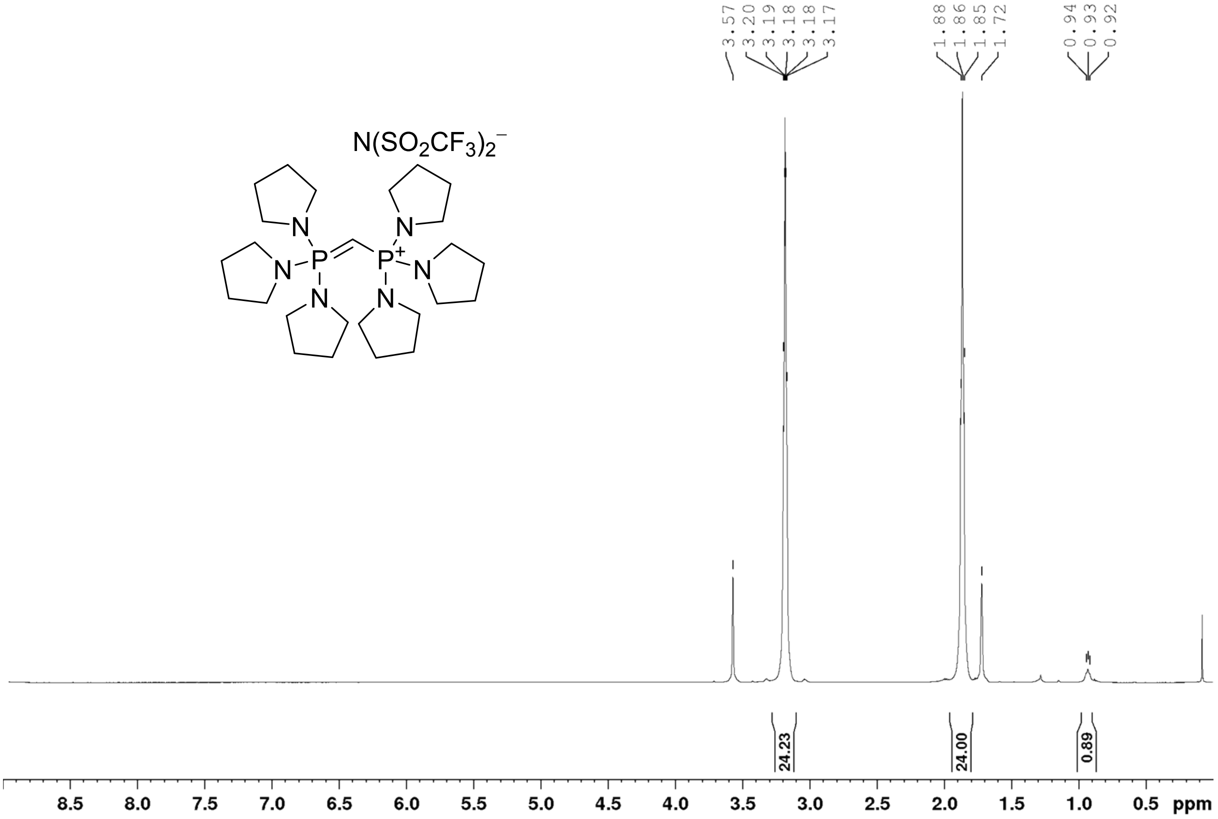

Figure S5: ^{1}H NMR spectrum of **4·HTFSI** (THF-d_8, 300 K, 500.2 MHz).

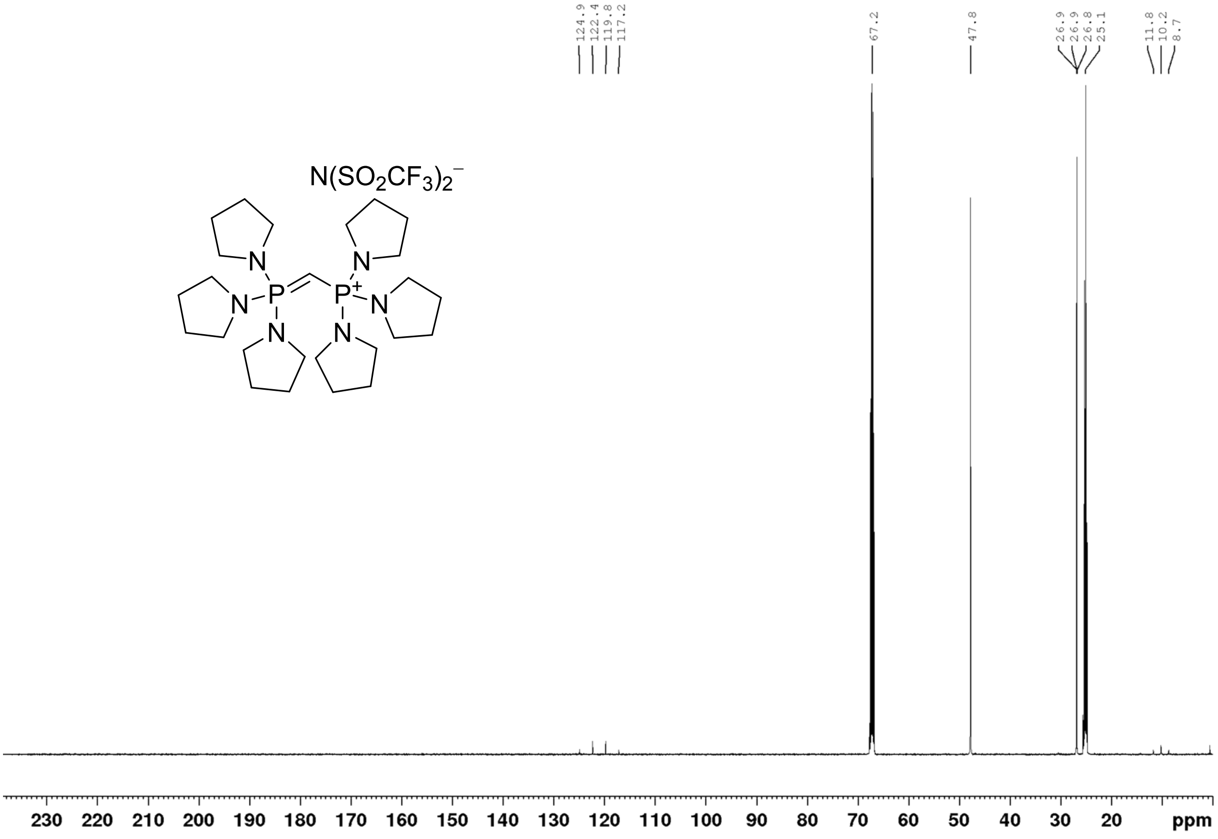

Figure S6: ^{13}C{^{1}H} NMR spectrum of **4·HTFSI** (THF-d_8, 300 K, 125.8 MHz).

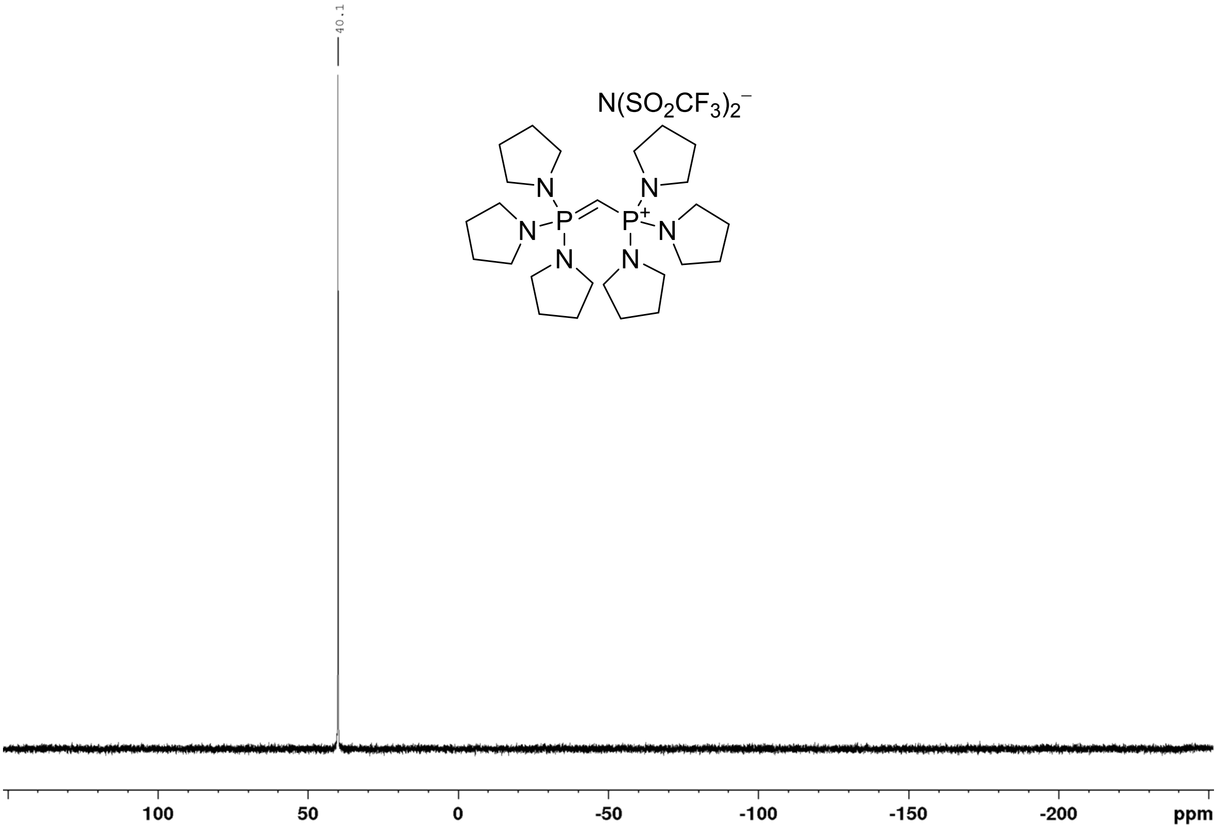

Figure S7: $^{31}P\{^{1}H\}$ NMR spectrum of **4·HTFSI** (THF-d_8, 300 K, 121.5 MHz).

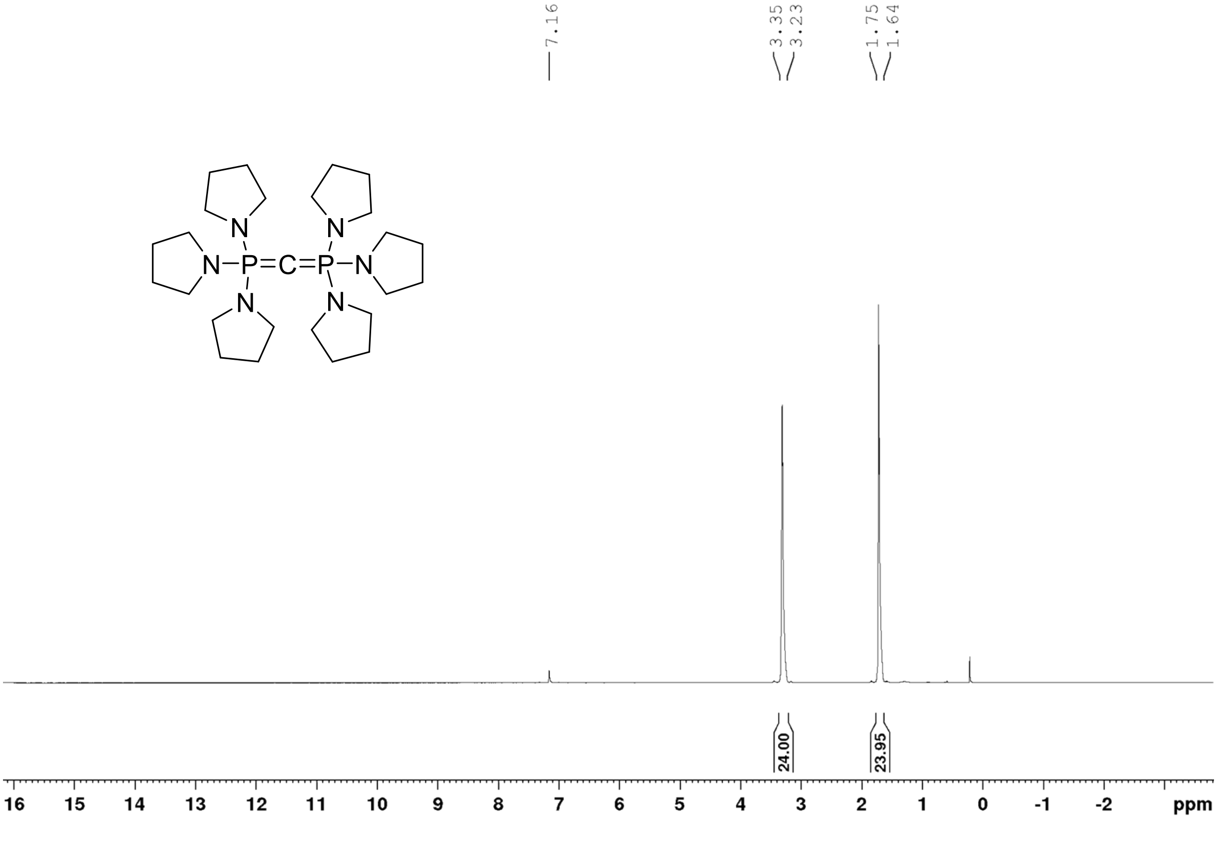

Figure S8: ^{1}H NMR spectrum of **4** (C_6D_6, 300 K, 500.2 MHz).

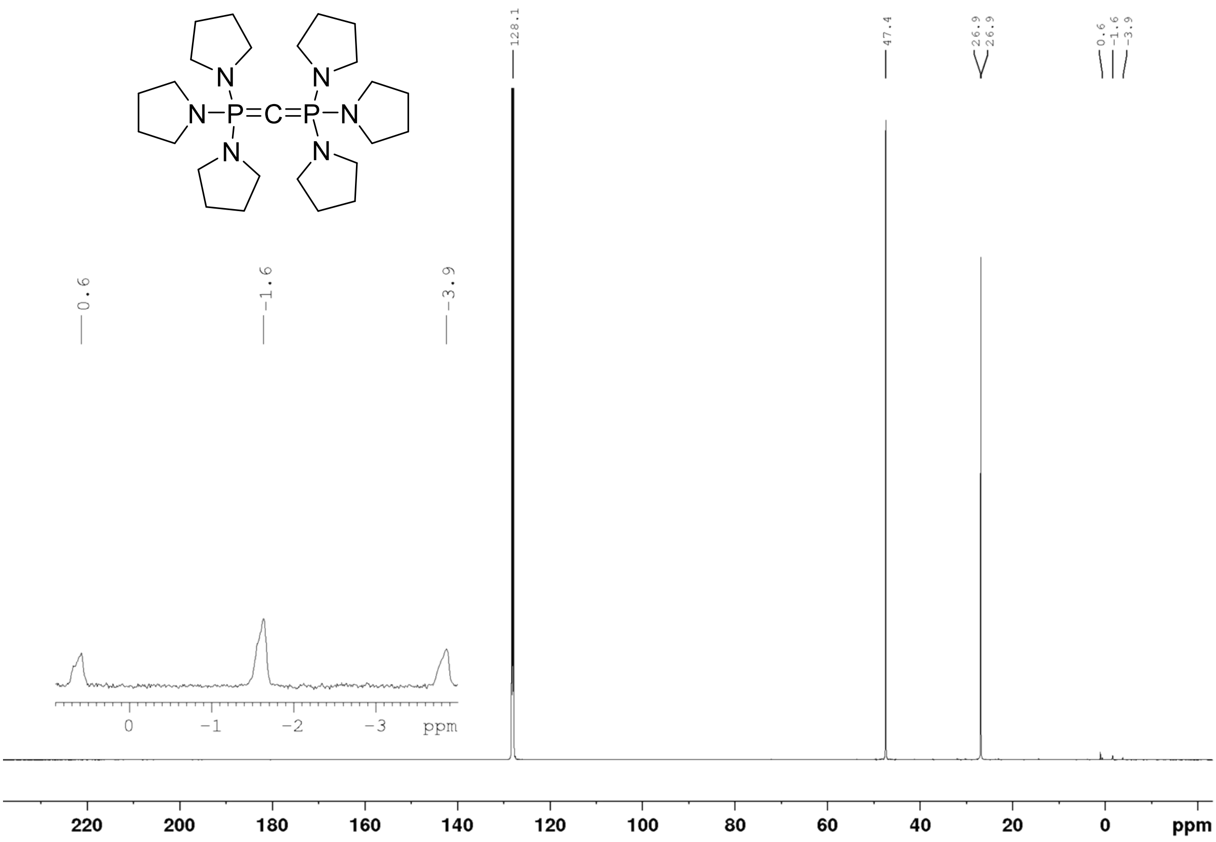

Figure S9: $^{13}C\{^1H\}$ NMR spectrum of **4** (C_6D_6, 300 K, 125.8 MHz).

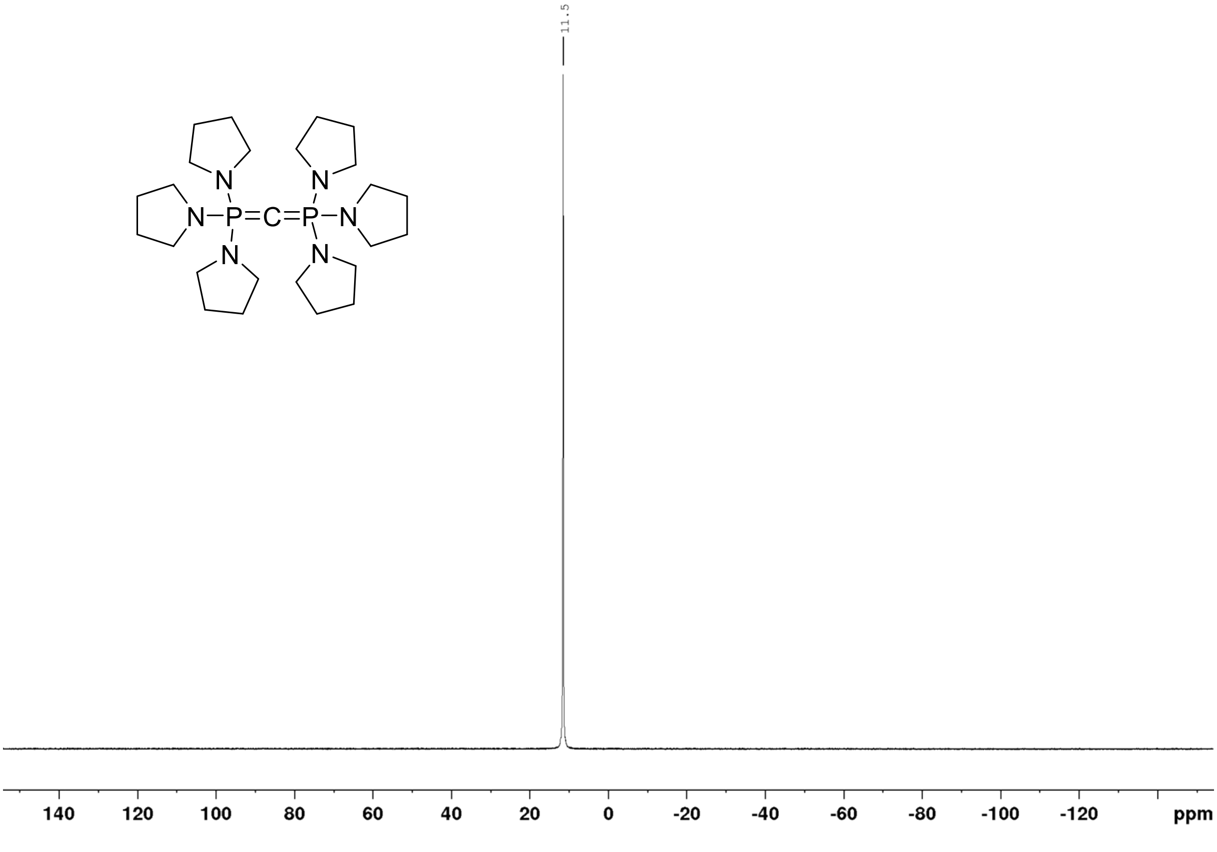

Figure S10: $^{31}P\{^1H\}$ NMR spectrum of **4** (C_6D_6, 300 K, 202.5 MHz).

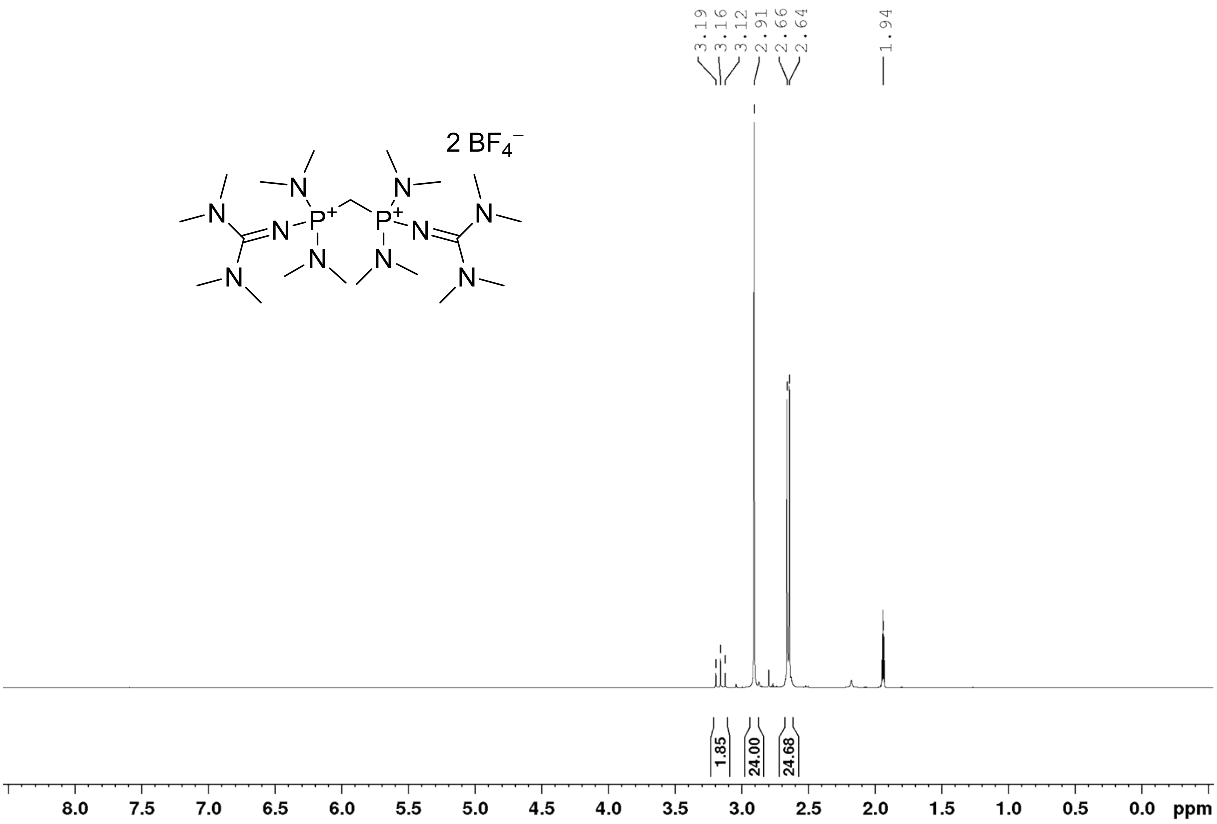

Figure S11: ^{1}H NMR spectrum of **1·2HBF$_4$** (CD_3CN, 300 K, 500.2 MHz).

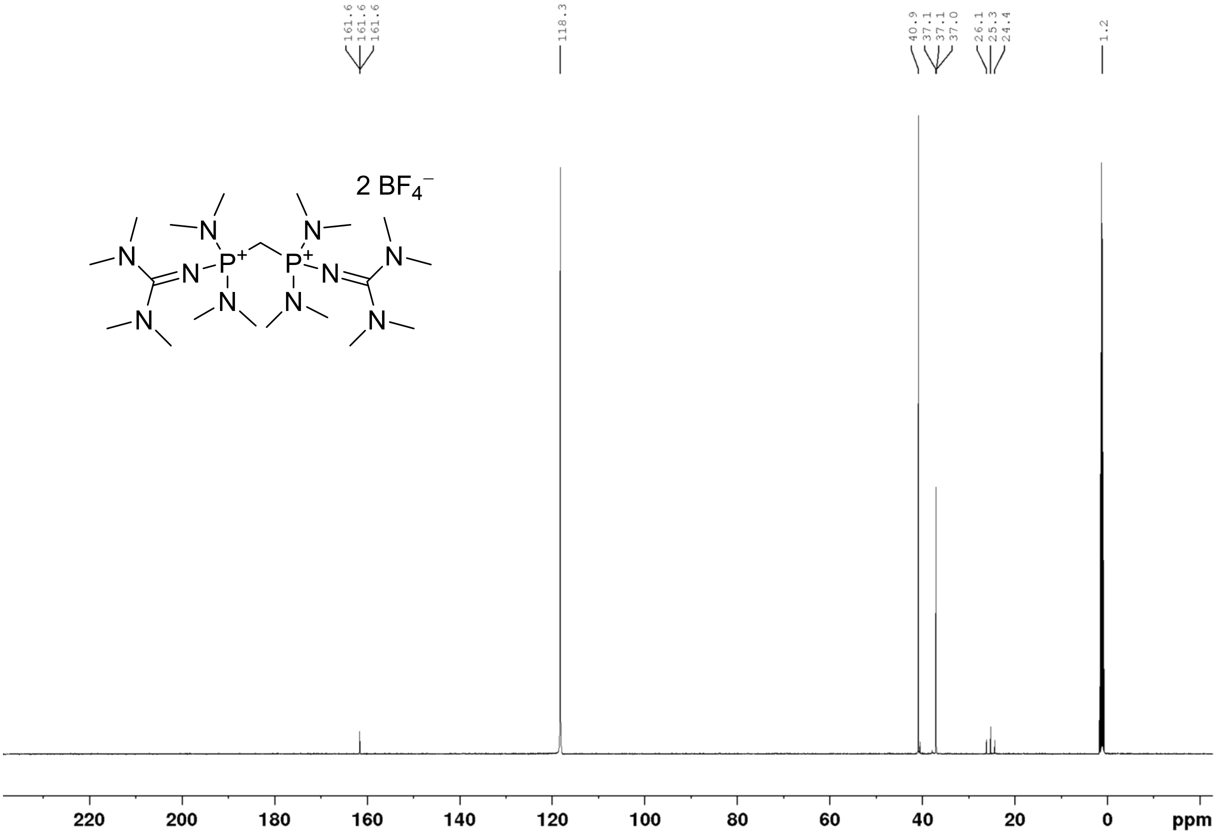

Figure S12: $^{13}C\{^{1}H\}$ NMR spectrum of **1·2HBF$_4$** (CD_3CN, 300 K, 125.8 MHz).

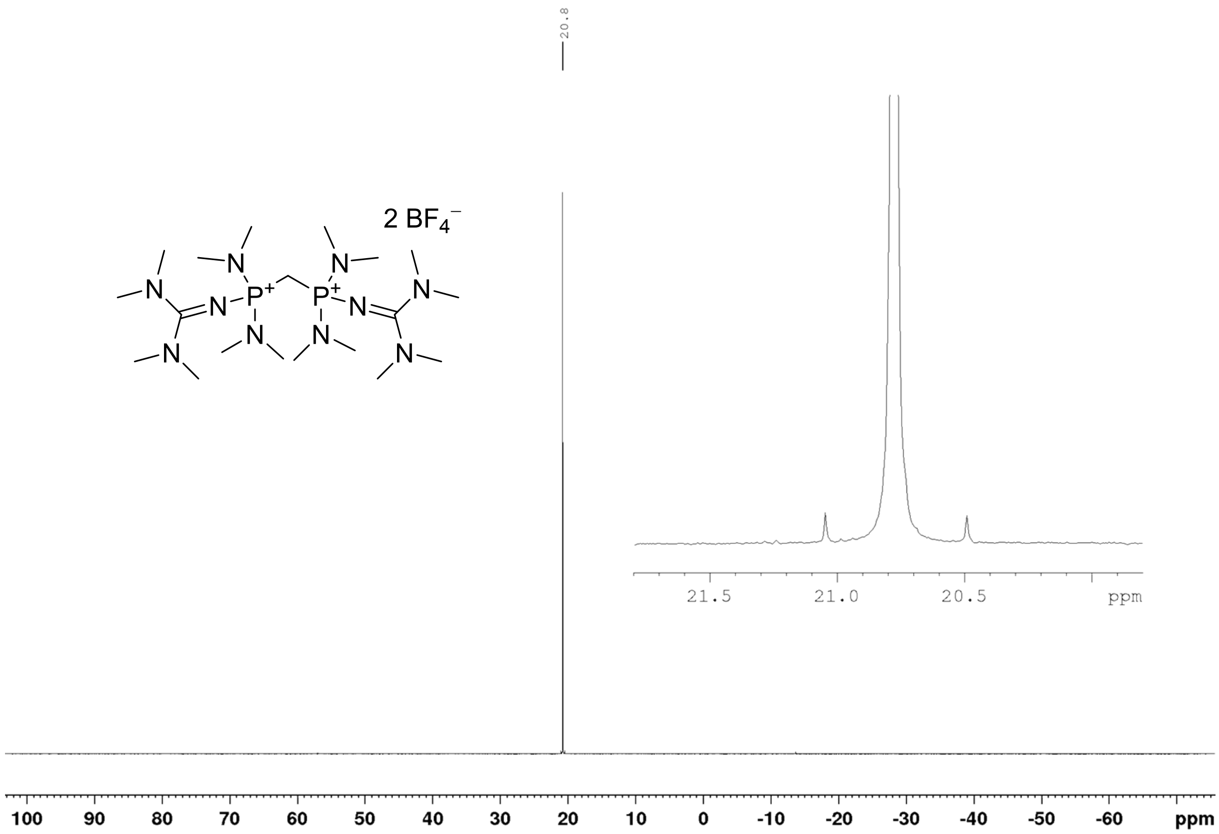

Figure S13: $^{31}P\{^{1}H\}$ NMR spectrum of **1·2HBF$_4$** (CD_3CN, 300 K, 202.5 MHz).

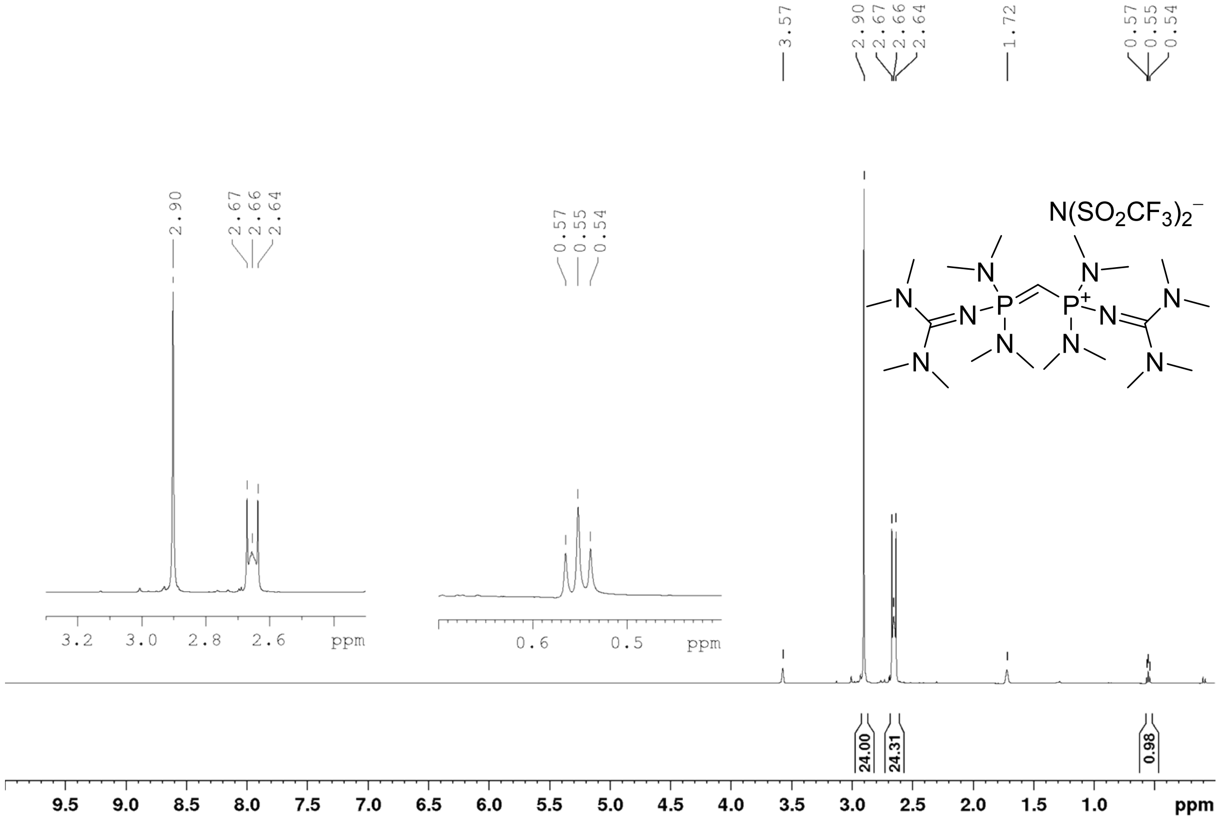

Figure S14: ^{1}H NMR spectrum of **1·HTFSI** (THF-d_8, 300 K, 300.3 MHz).

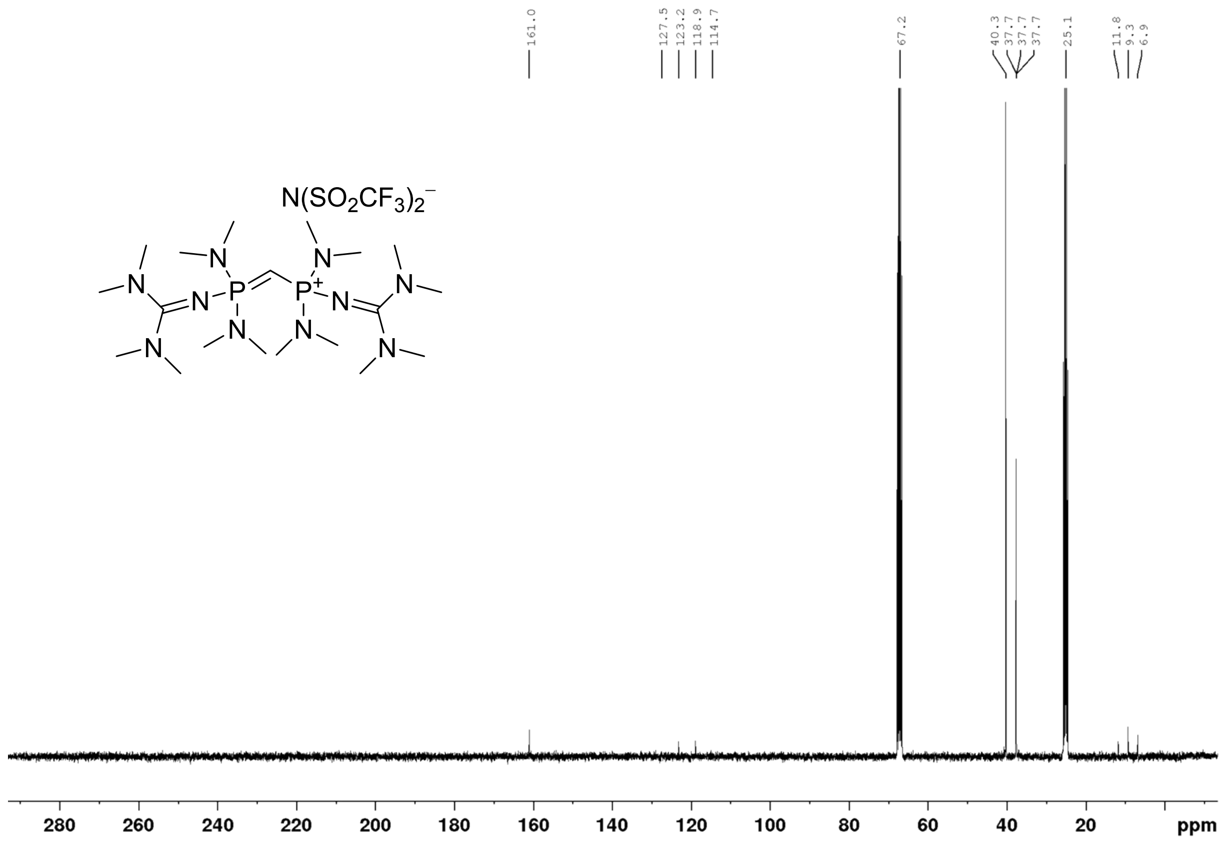

Figure S15: $^{13}C\{^{1}H\}$ NMR spectrum of **1·HTFSI** (THF-d_8, 300 K, 75.5 MHz).

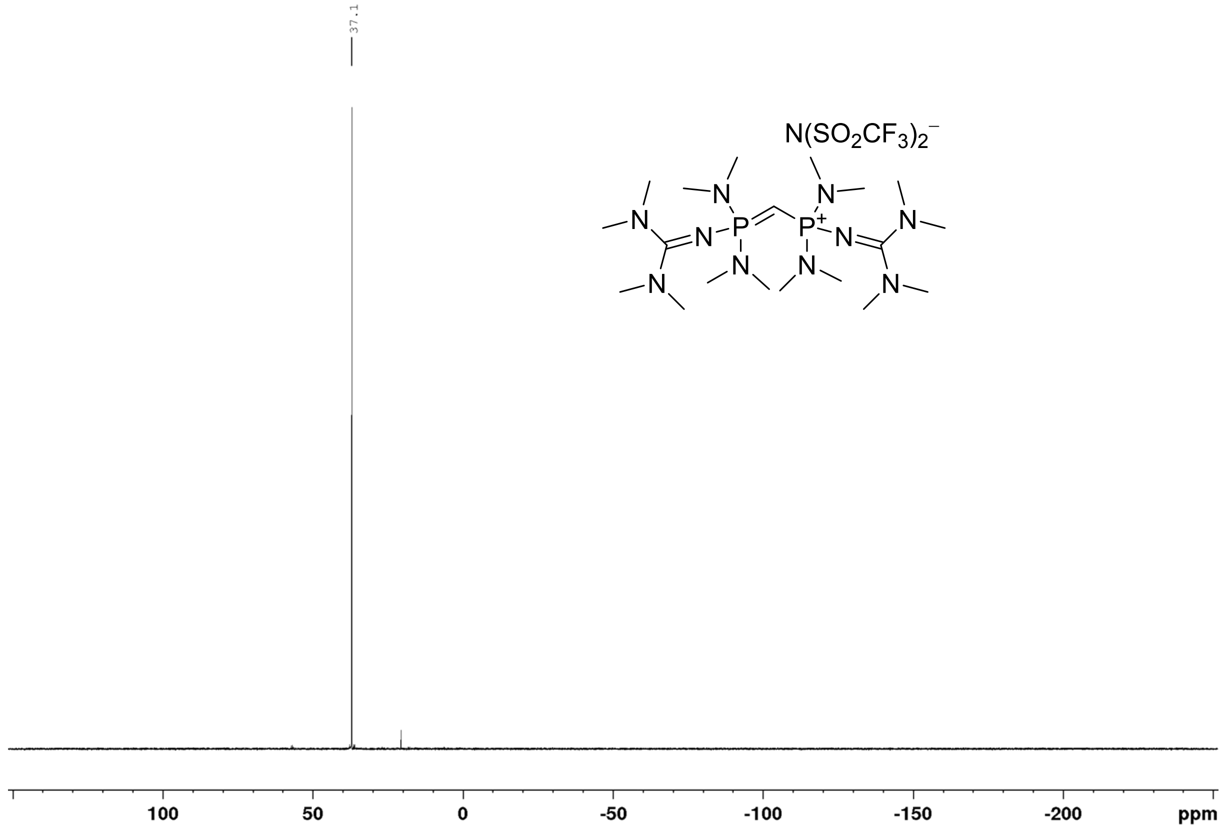

Figure S16: $^{31}P\{^{1}H\}$ NMR spectrum of **1·HTFSI** (THF-d_8, 300 K, 121.5 MHz).

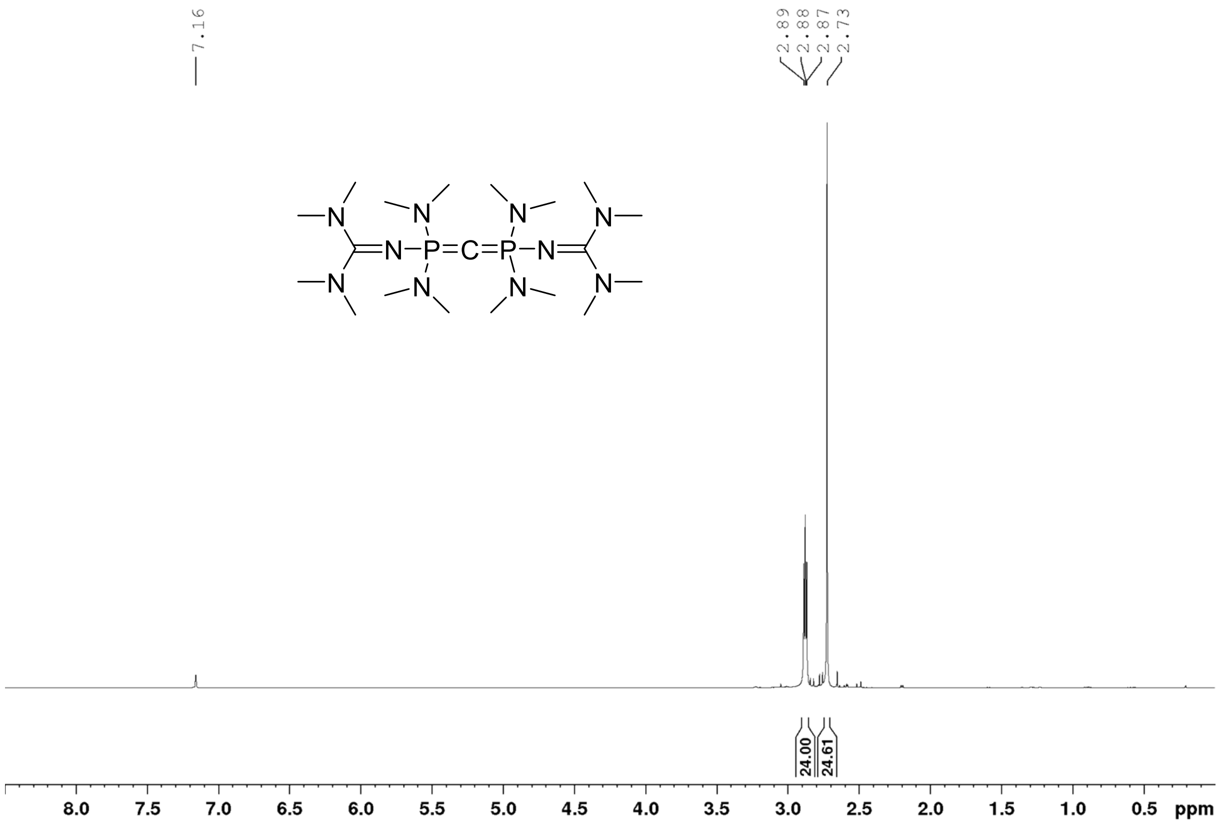

Figure S17: ^{1}H NMR spectrum of **1** (C_6D_6, 300 K, 500.2 MHz).

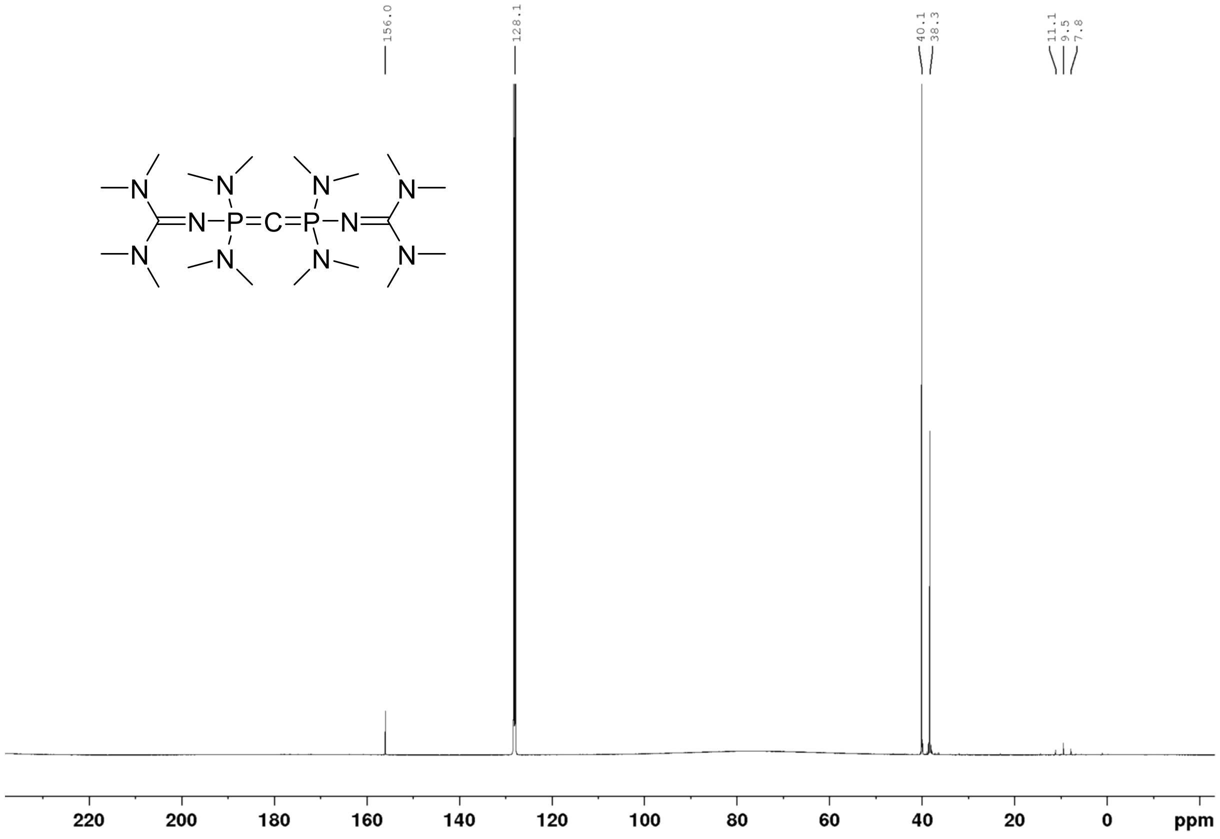

Figure S18: $^{13}C\{^{1}H\}$ NMR spectrum of **1** (C_6D_6, 300 K, 125.8 MHz).

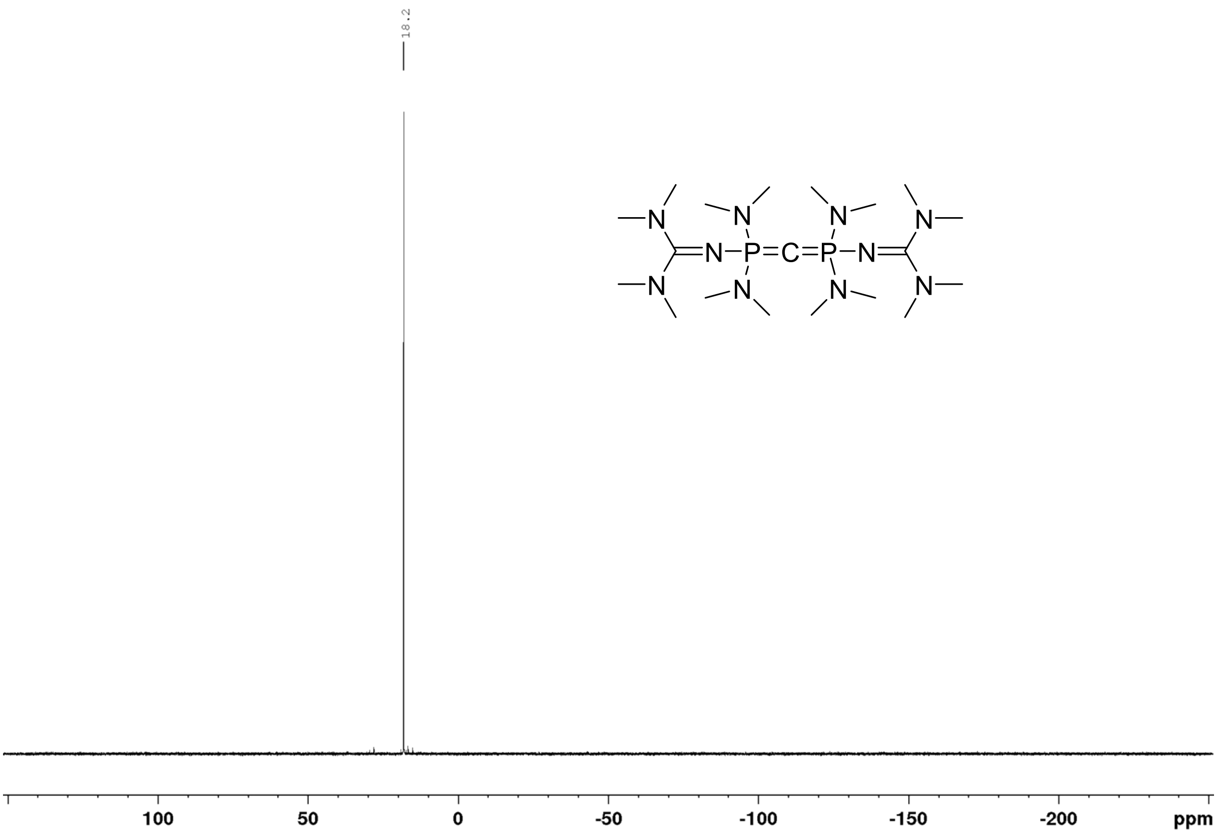

Figure S19: $^{31}P\{^{1}H\}$ NMR spectrum of **1** (C_6D_6, 300 K, 121.5 MHz).

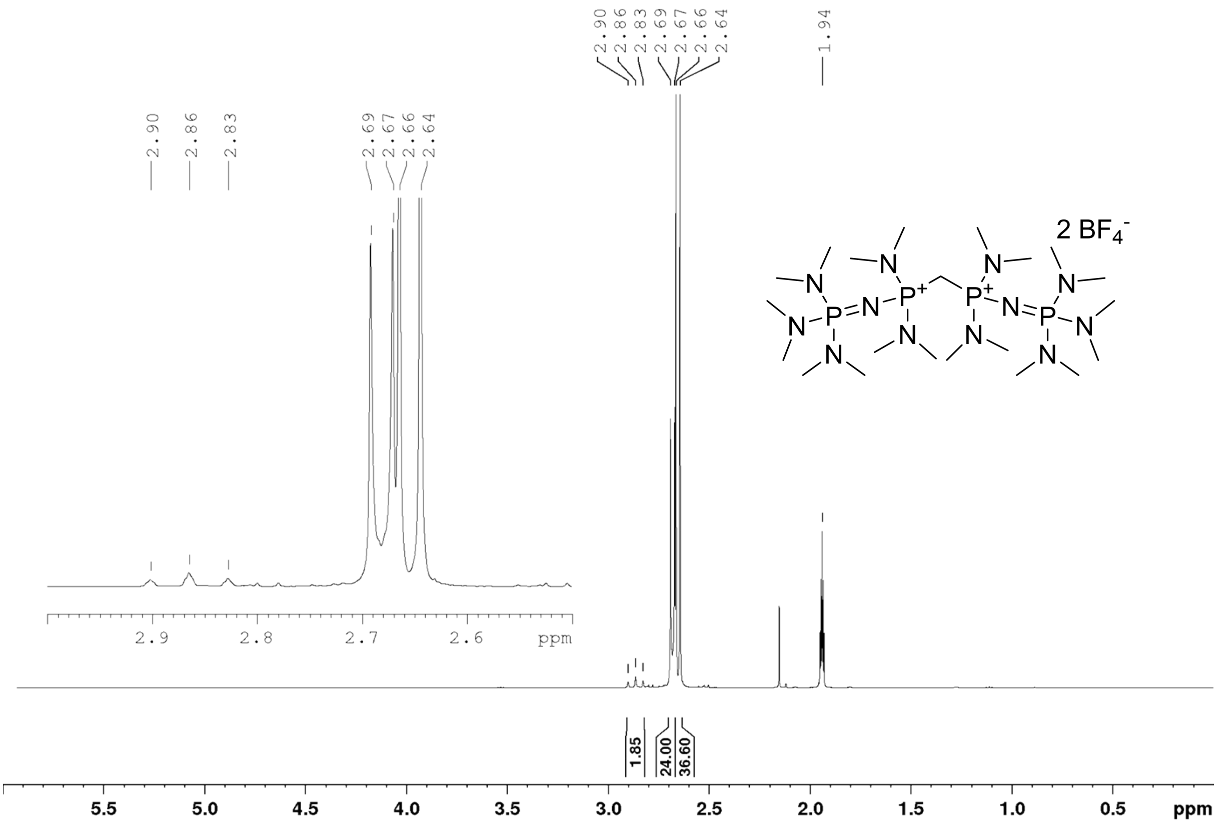

Figure S20: ^{1}H NMR spectrum of **2·2HBF$_4$** (CD_3CN, 300 K, 500.2 MHz).

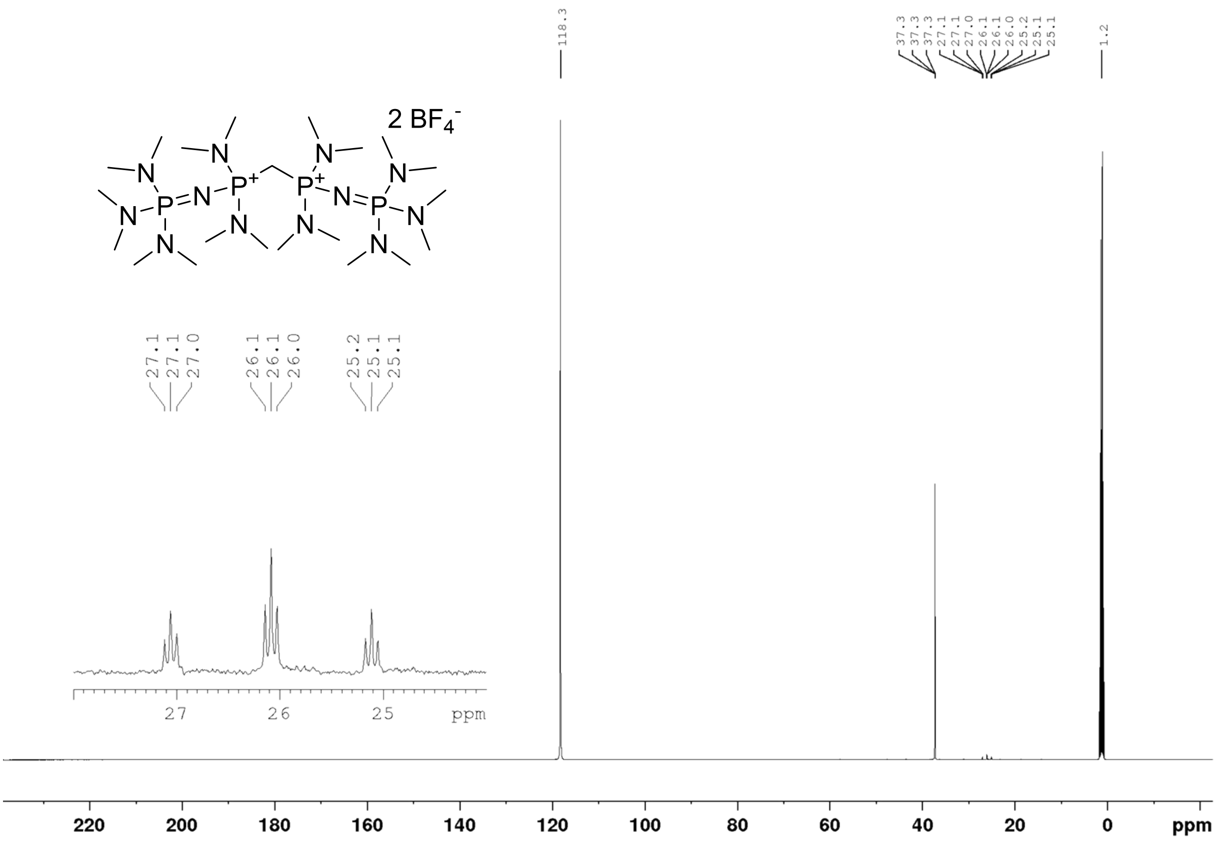

Figure S21: $^{13}C\{^{1}H\}$ NMR spectrum of **2·2HBF$_4$** (CD_3CN, 300 K, 125.8 MHz).

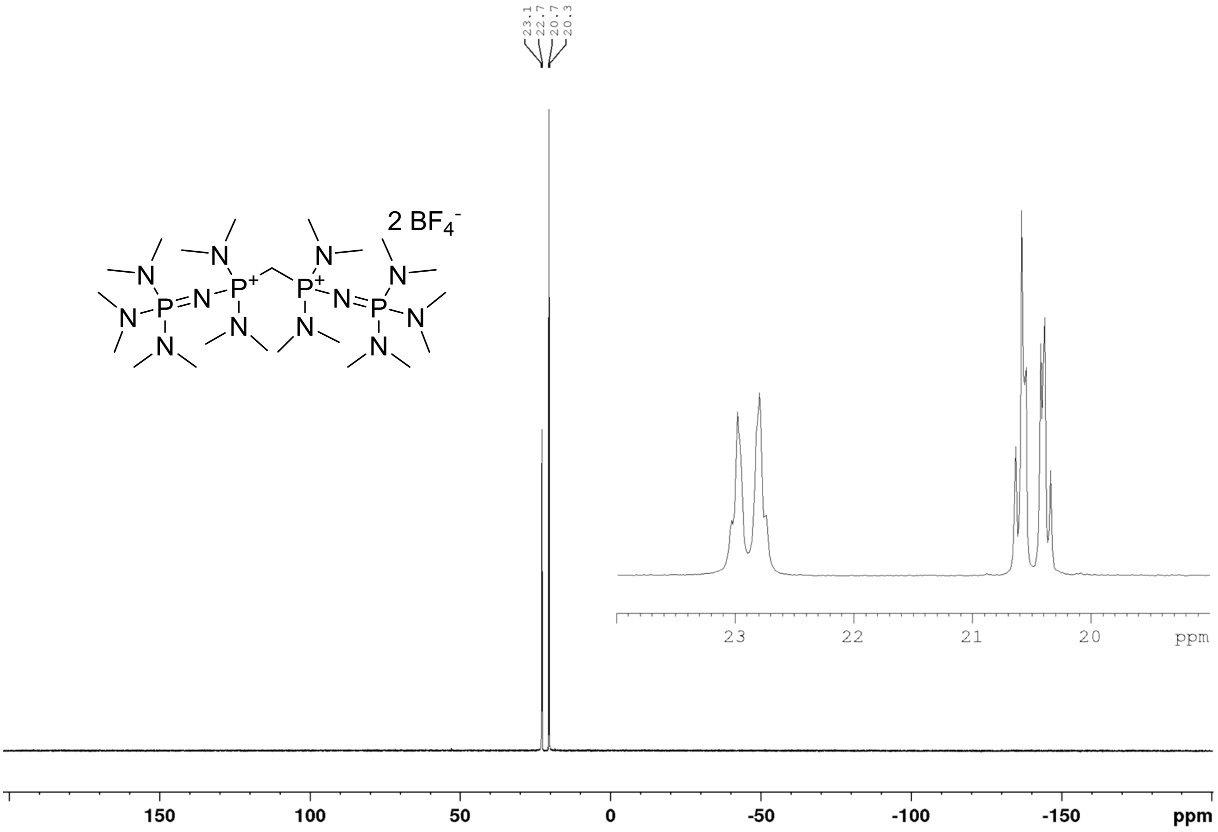

Figure S22: $^{31}P\{^{1}H\}$ NMR spectrum of **2·2HBF$_4$** (CD_3CN, 300 K, 202.5 MHz).

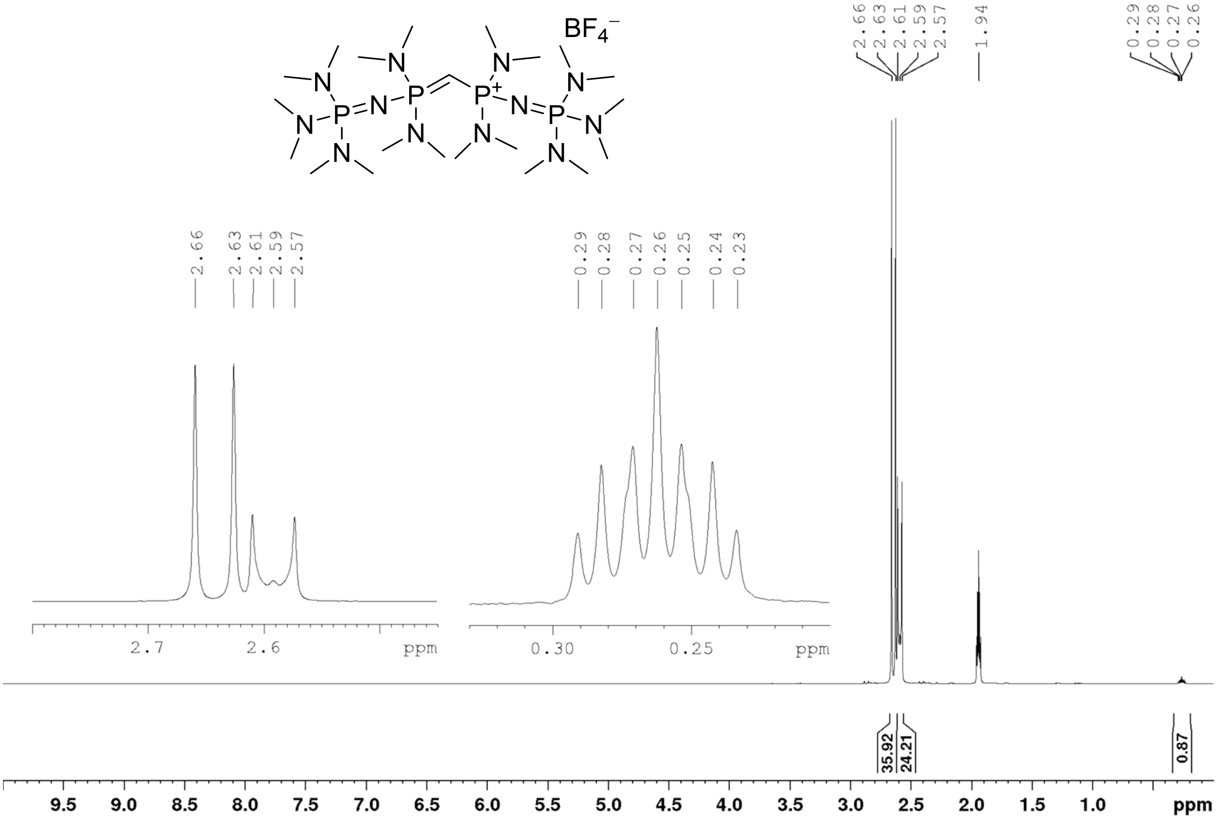

Figure S23: ^{1}H NMR spectrum of **2·HBF₄** (CD_3CN, 300 K, 500.2 MHz).

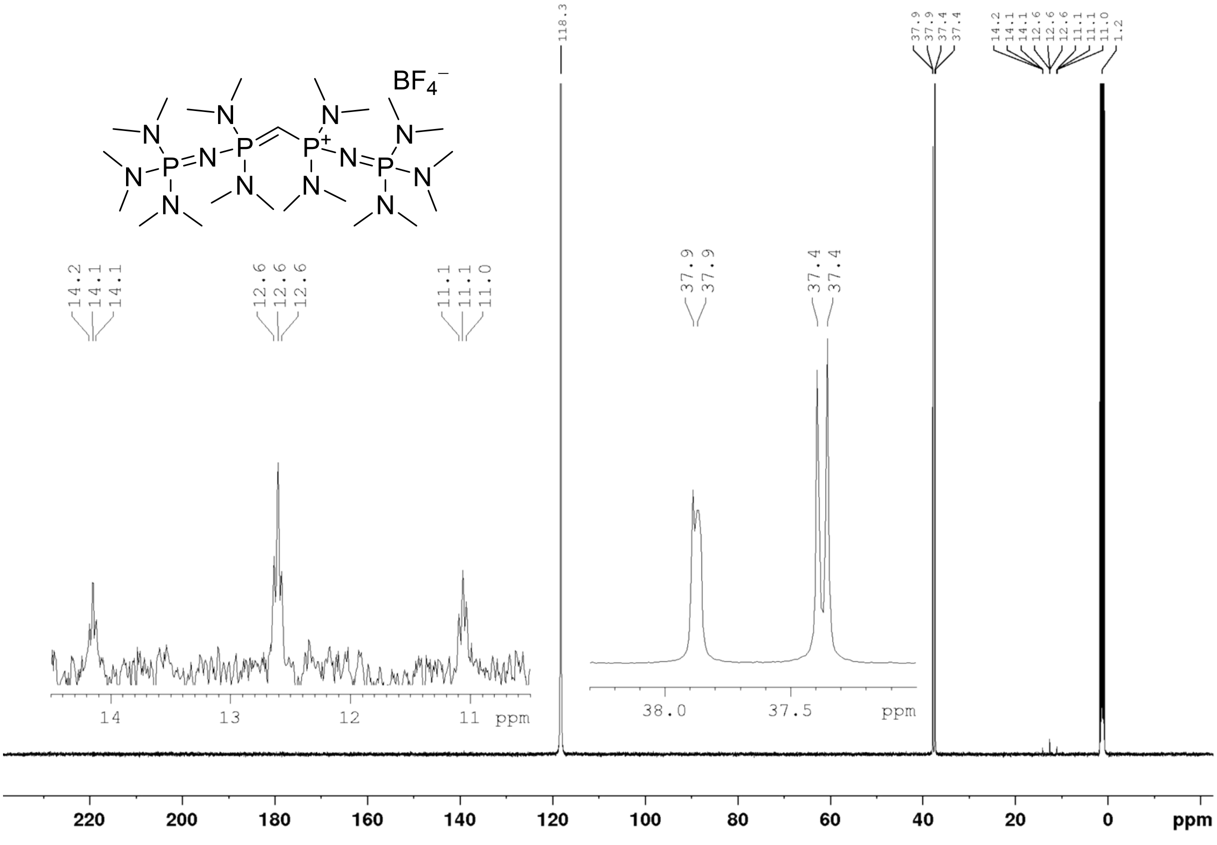

Figure S24: $^{13}C\{^{1}H\}$ NMR spectrum of **2·HBF₄** (CD_3CN, 300 K, 125.8 MHz).

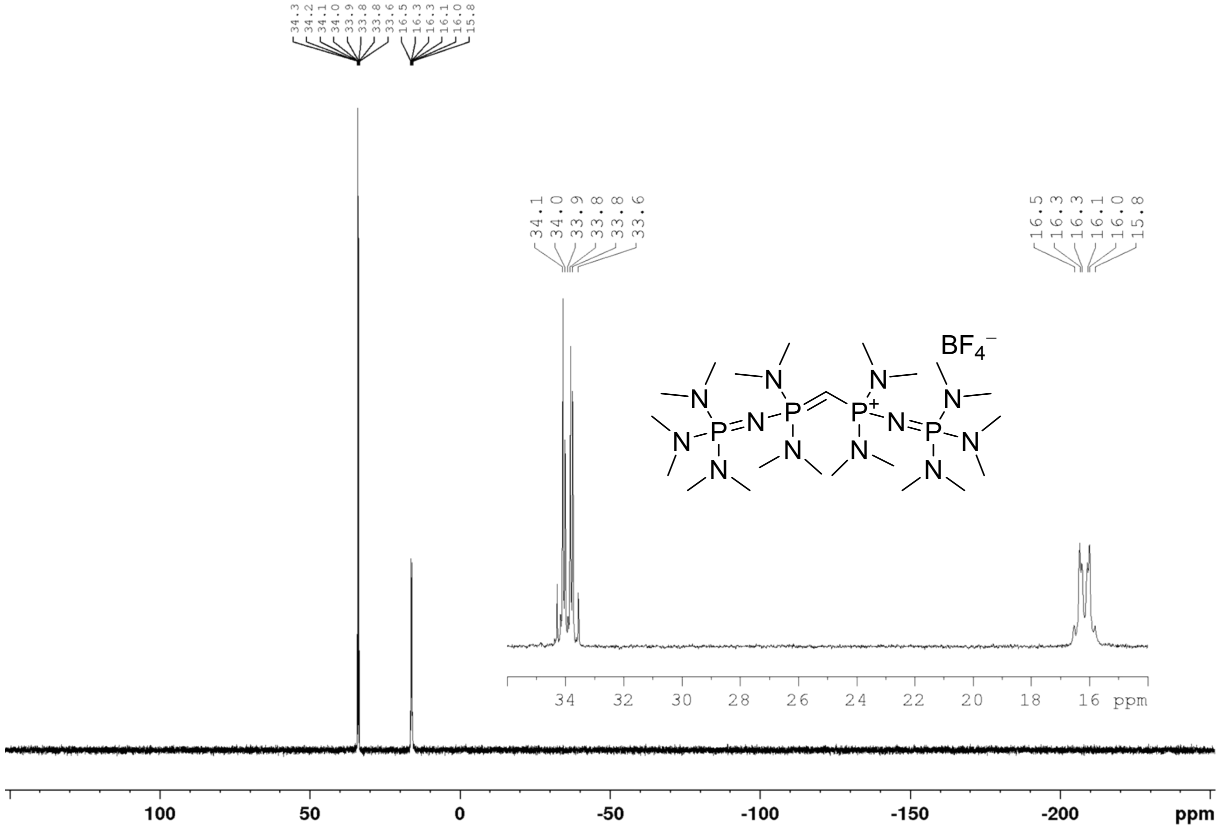

Figure S25: $^{31}P\{^{1}H\}$ NMR spectrum of **2·HBF4** (CD_3CN, 300 K, 121.5 MHz).

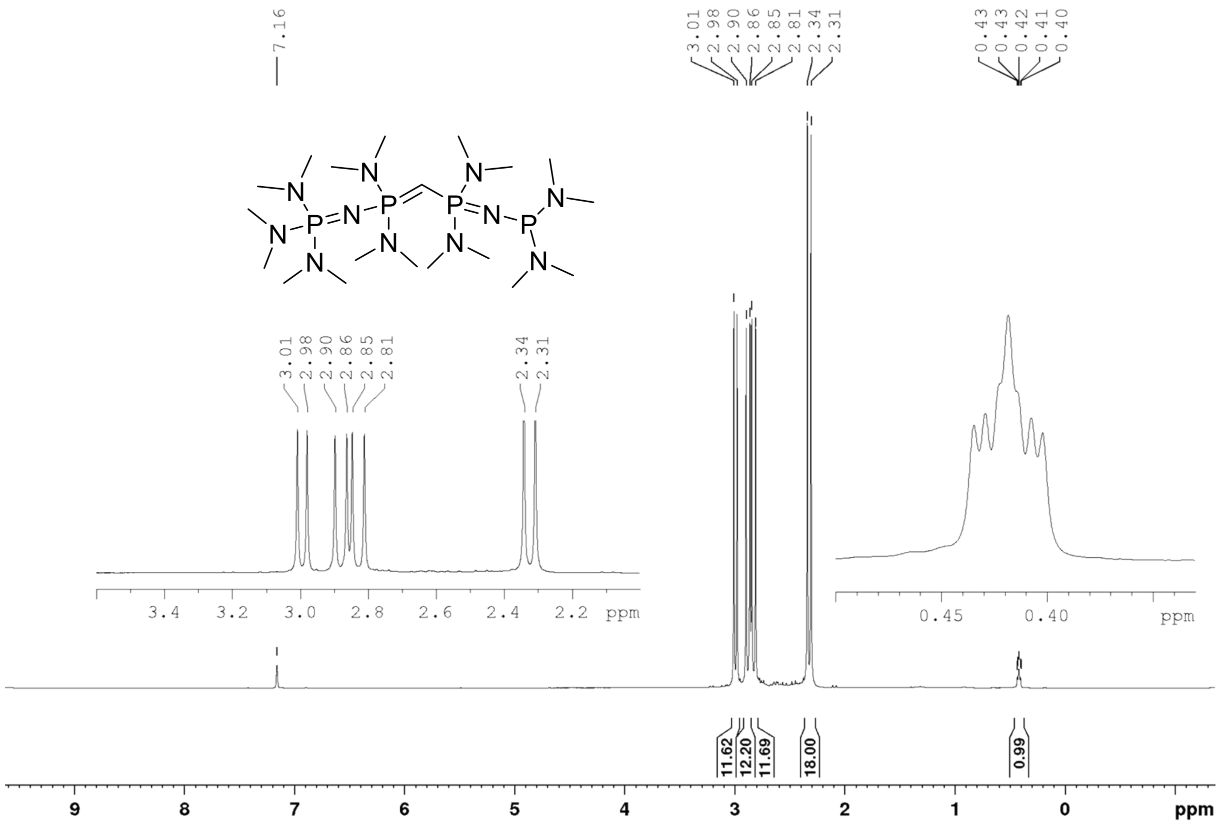

Figure S26: ^{1}H NMR spectrum of **7** (C_6D_6, 300 K, 300.3 MHz).

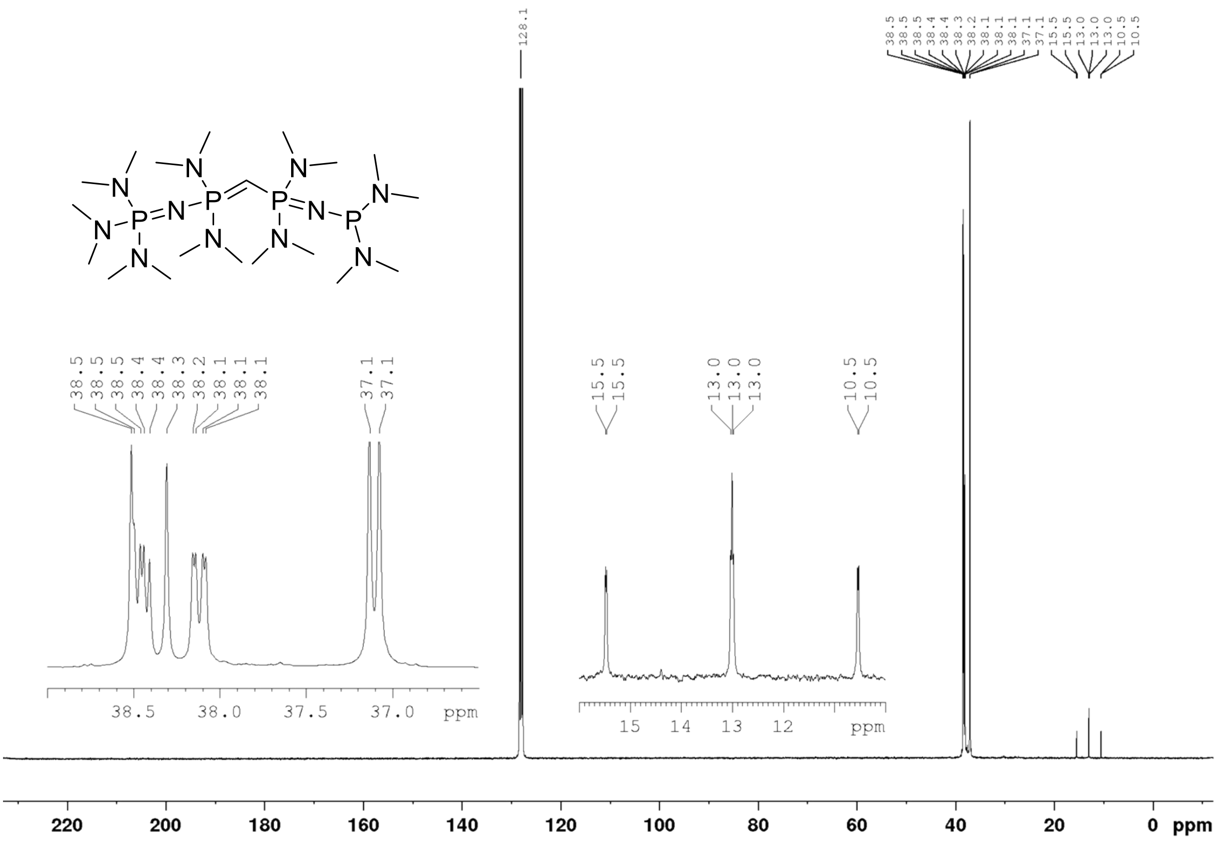

Figure S27: $^{13}C\{^1H\}$ NMR spectrum of **7** (C_6D_6, 300 K, 75.5 MHz).

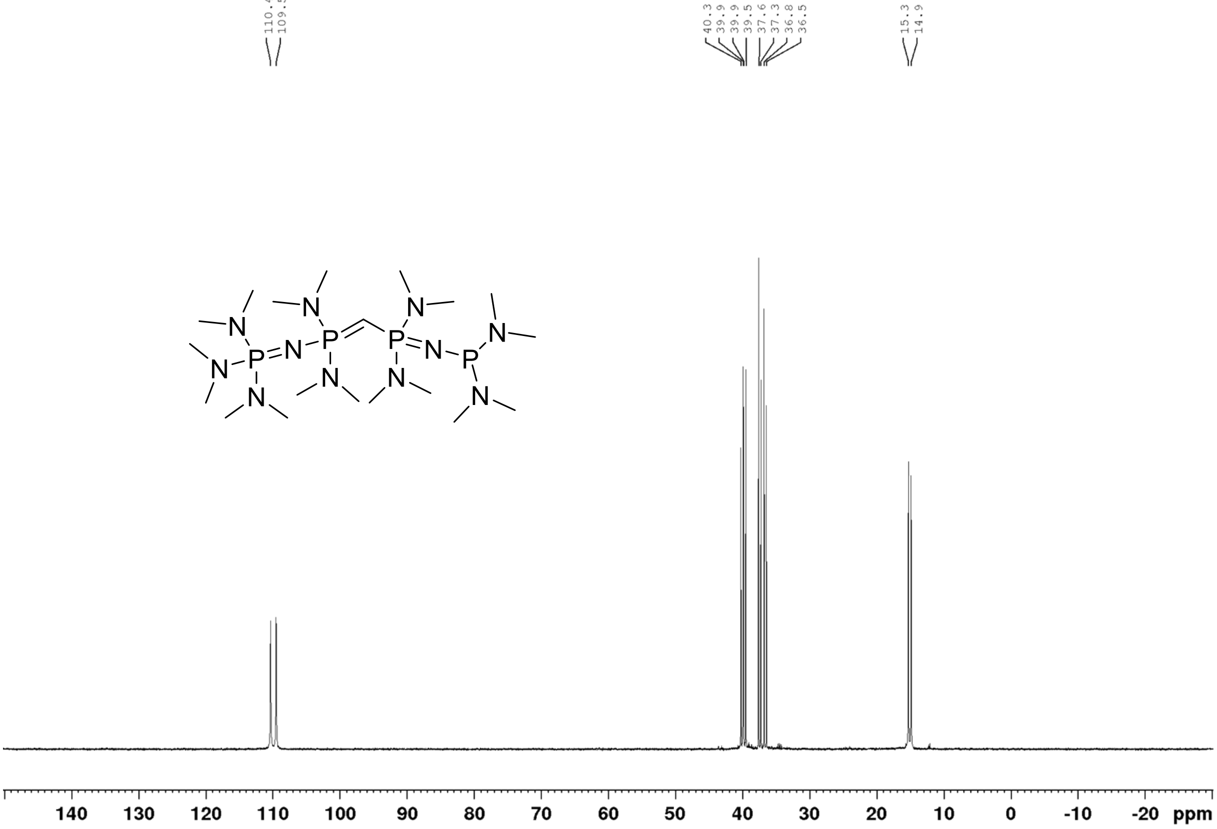

Figure S28: $^{31}P\{^1H\}$ NMR spectrum of **7** (C_6D_6, 300 K, 121.5 MHz).

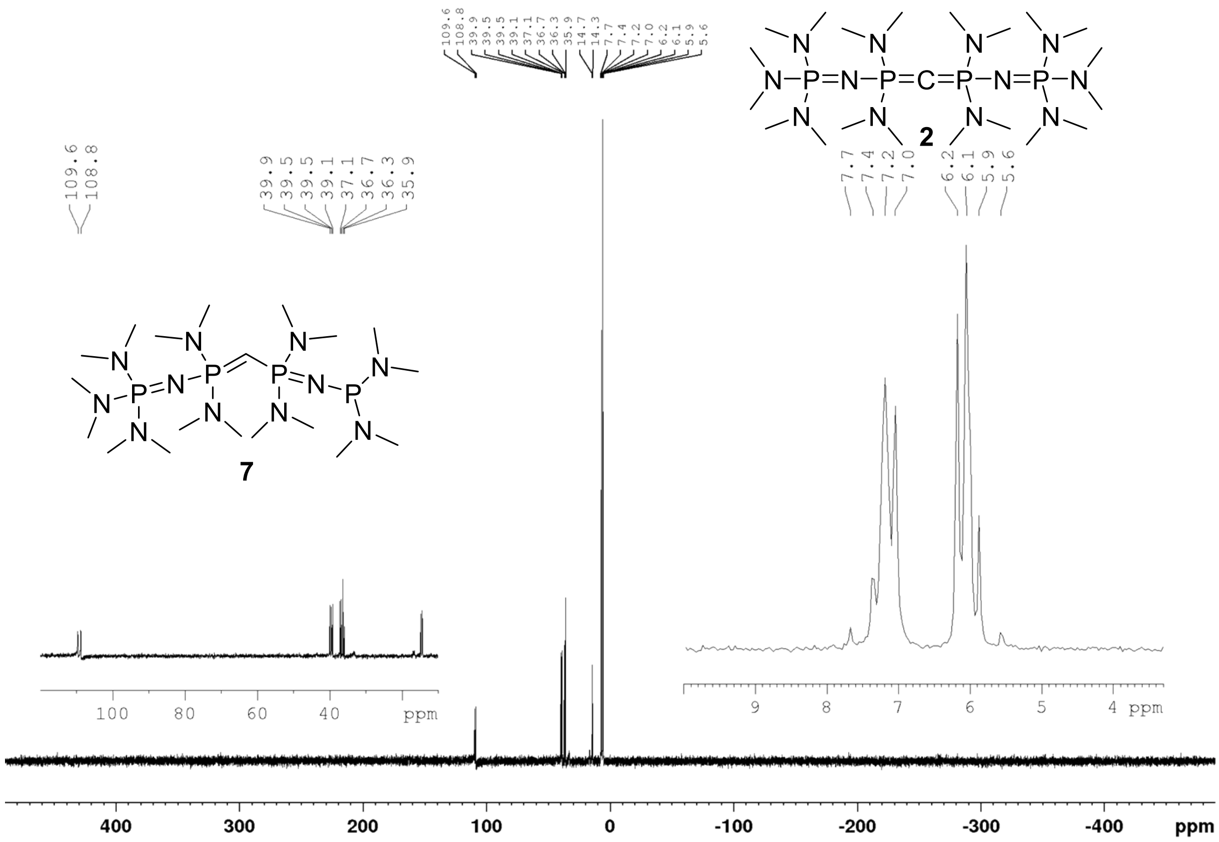

Figure S29: $^{31}P\{^{1}H\}$ NMR spectrum of the isolated product of the deprotonation of **2·2HBF$_4$** with potassium hydride in THF (C_6D_6, 300 K, 121.5 MHz).

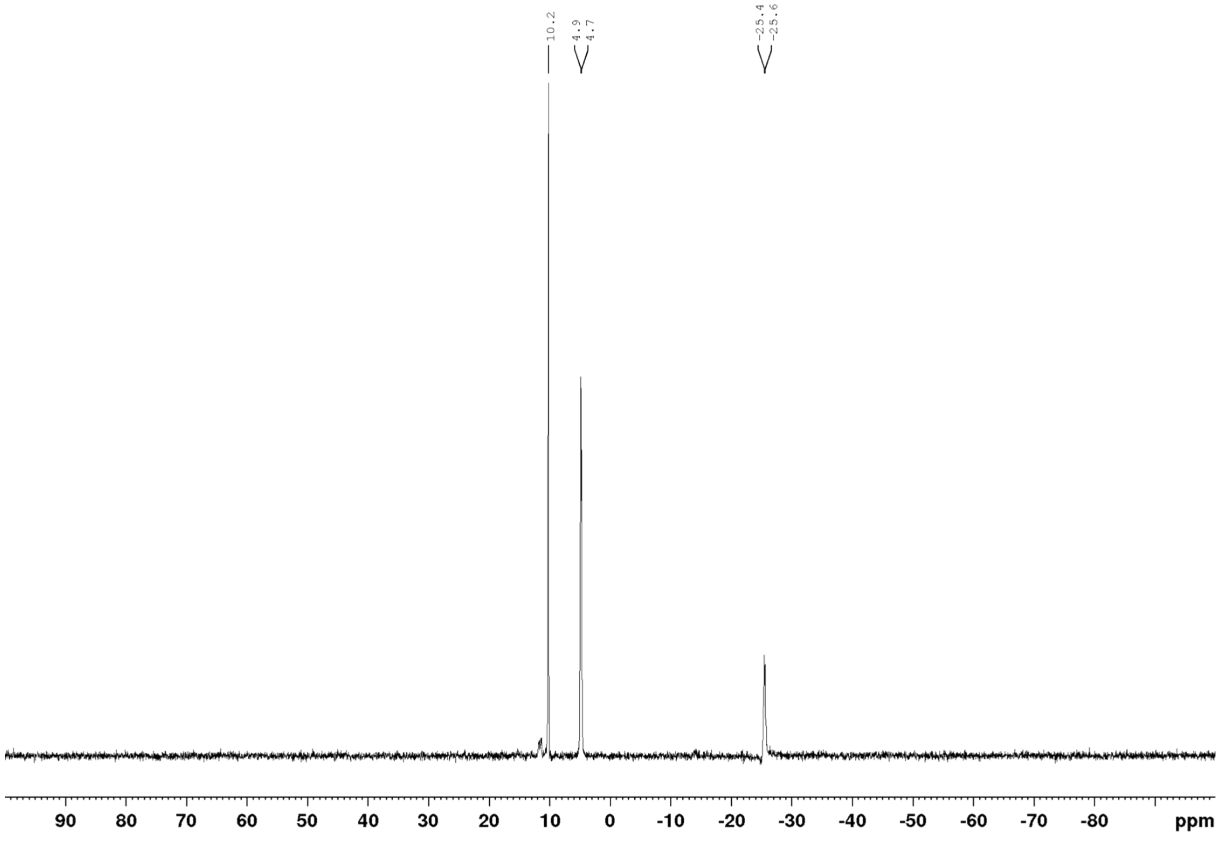

Figure S30: $^{31}P\{^{1}H\}$ NMR spectrum of a 1:1 mixture of **4** and (dma)P_4-*t*Bu (THF-d_8, 300 K, 121.5 MHz).

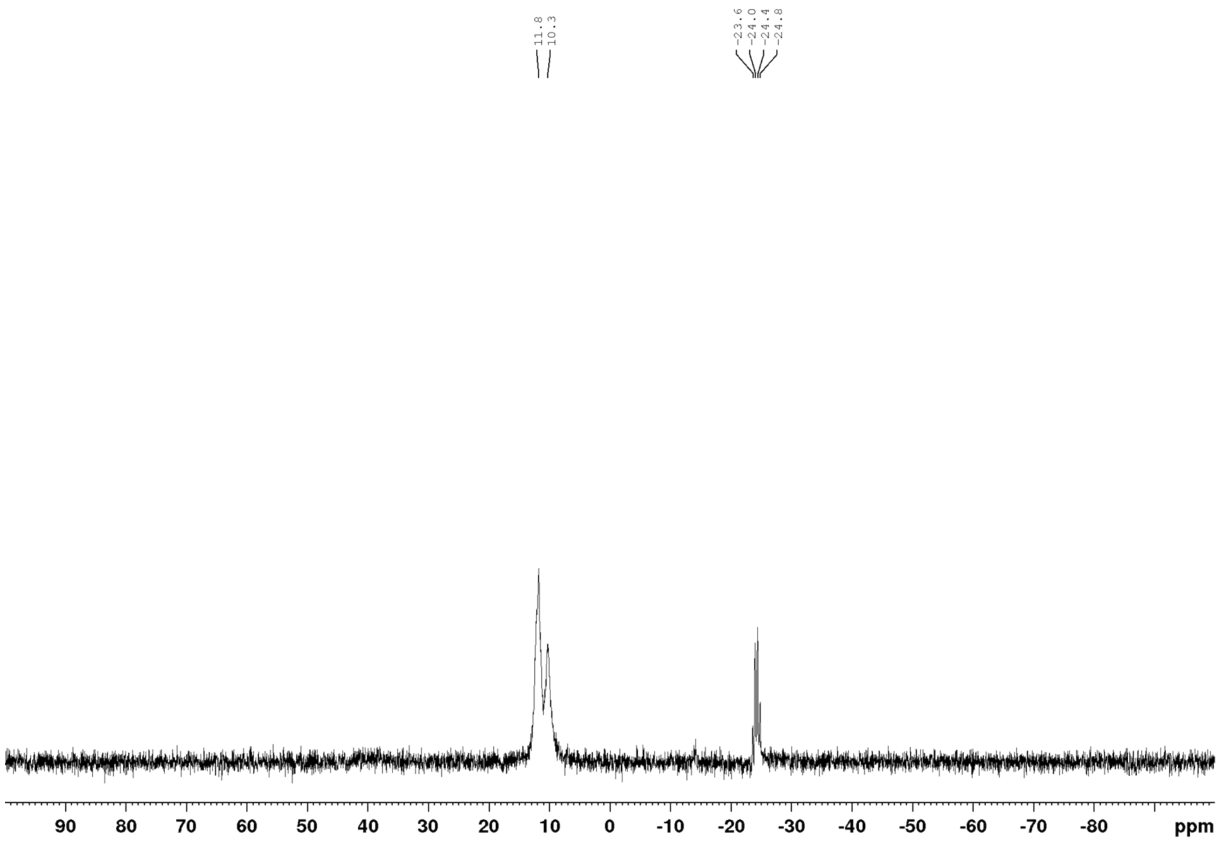

Figure S31: $^{31}P\{^{1}H\}$ NMR spectrum of a 1:1 mixture of **4** and (dma)P_4-*t*Bu after adding HTFSI (THF-d_8, 300 K, 121.5 MHz).

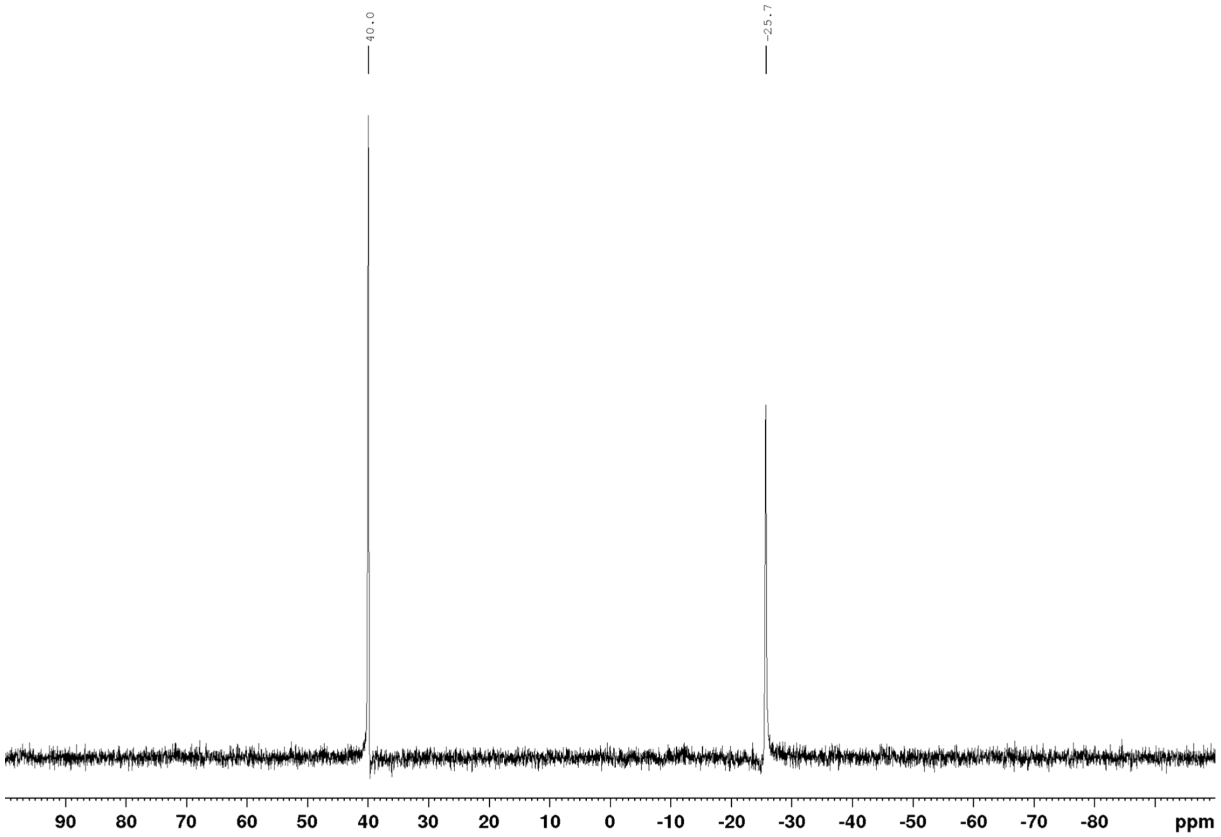

Figure S32: $^{31}P\{^{1}H\}$ NMR spectrum of a 1:1 mixture of **4** and (tmg)P_1-*t*Bu·HBF_4 (THF-d_8, 300 K, 121.5 MHz).

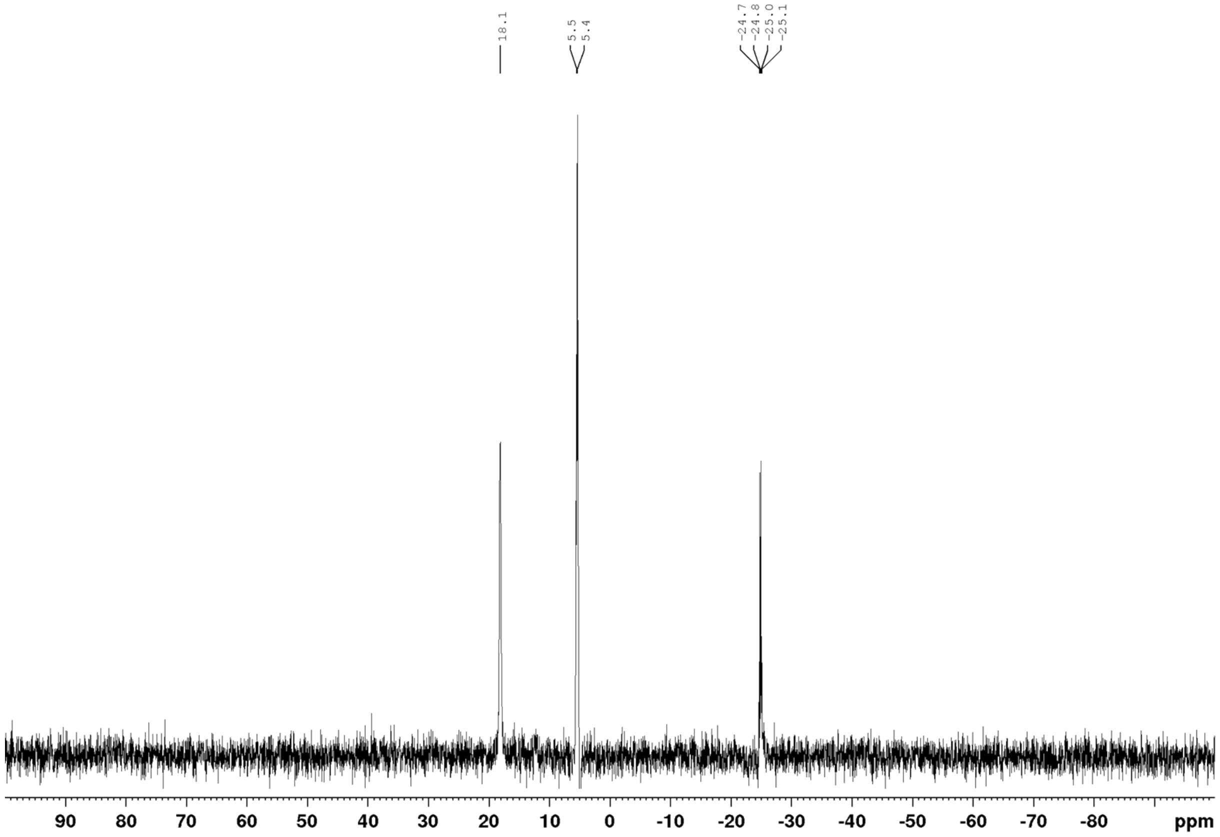

Figure S33: $^{31}P\{^{1}H\}$ NMR spectrum of a 1:1 mixture of **1** and (dma)P_4-*t*Bu (THF-d_8, 300 K, 121.5 MHz).

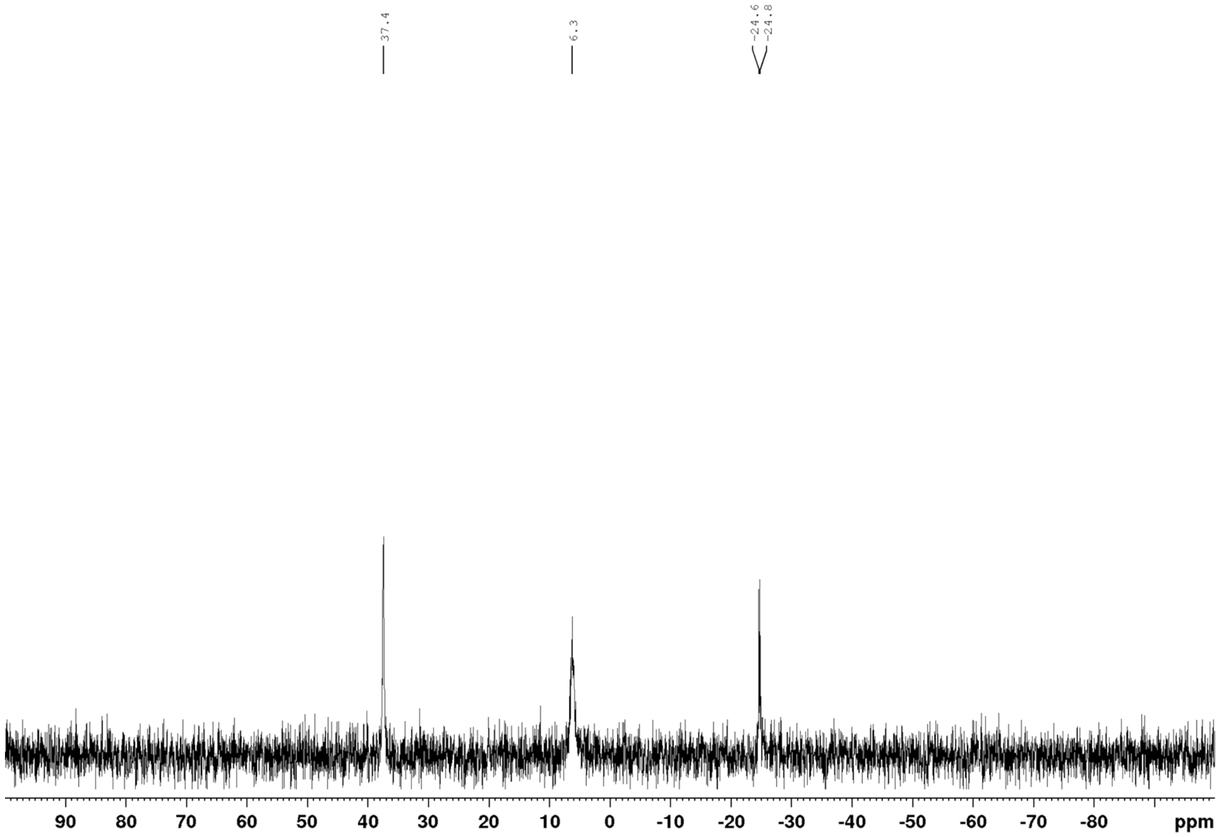

Figure S34: $^{31}P\{^{1}H\}$ NMR spectrum of a 1:1 mixture of **1** and (dma)P_4-*t*Bu after adding HTFSI (THF-d_8, 300 K, 121.5 MHz).

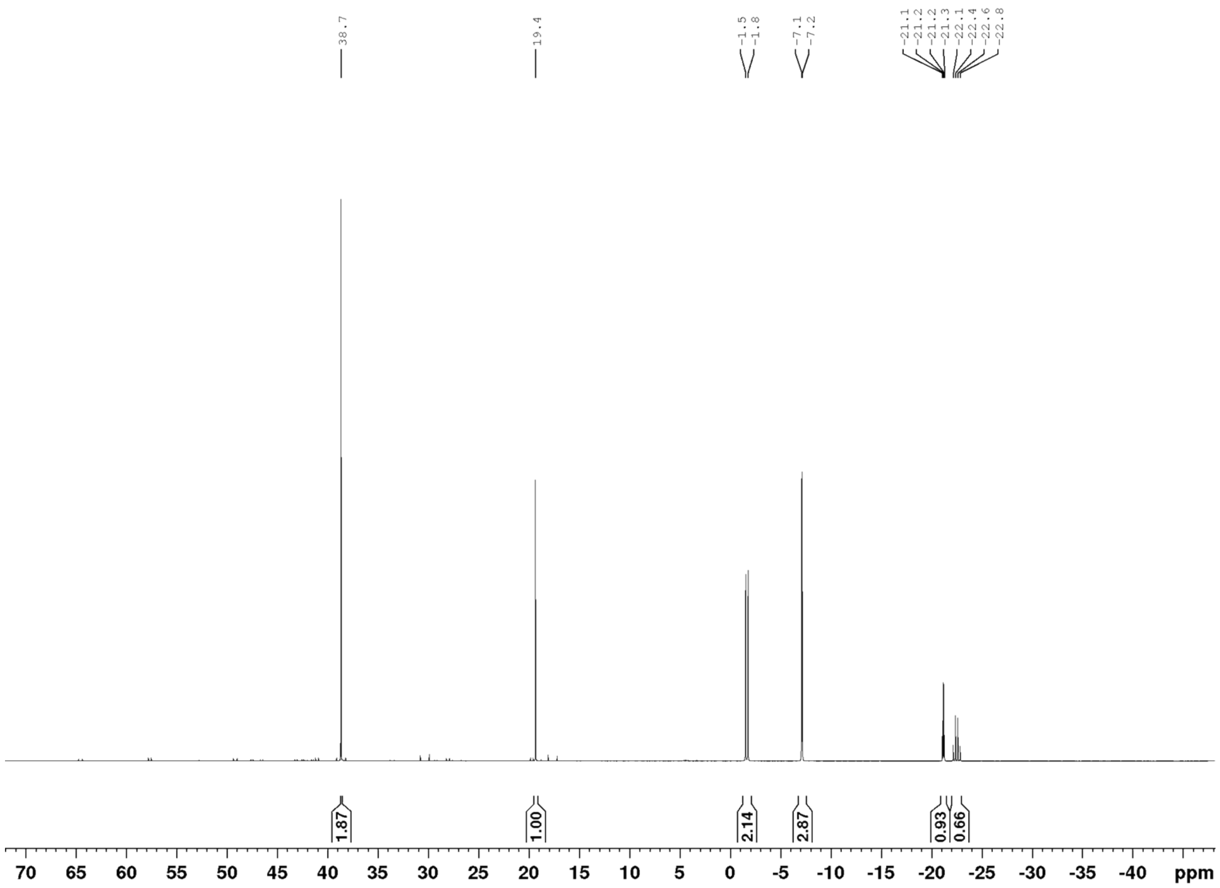

Figure S35: $^{31}P\{^{1}H\}$ NMR spectrum of a 1:1 mixture of **1** and (pyrr)P_4-*t*Bu after adding HTFSI (THF-d_8, 300 K, 202.5 MHz).

Crystallographic Section

Data were collected with a Bruker D8 Quest area detector diffractometer equipped with MoK_α radiation, a graded multilayer mirror monochromator (λ = 0.71073 Å) and a Photon-100 CMOS detector or with a Stoe Stadivari diffractometer equipped with CuK_α radiation, a graded multilayer mirror monochromator (λ = 1.54178 Å) and a Dectris Pilatus 300K detector, both using an oil-coated shock-cooled crystal at 100(2) K. Data collection, reduction, cell refinement and semi-empirical absorption correction (multi-scan) were performed within Bruker Apex3[3] or Stoe X-Area.[4] Structures were solved with dual-space methods using ShelXT[5] and refined against F^2 with ShelXL,[6] all within the user interface of WinGX[7] and ShelXLe.[8] Carbon bonded hydrogen atoms were calculated in their idealized positions and refined with fixed isotropic thermal parameters. Hydrogen atoms connected to heteroatoms were located on the Fourier map and refined isotropically. All molecular structures were illustrated with Diamond 4[9] using thermal ellipsoids at the 50% probability level. Peripheral protons as well as non-coordinating solvent molecules are omitted for clarity. In case of disorder only the major component is displayed. Atom colours are assigned as shown below with reference to Jmol.[10]

H	B	C	N	O	F	P	Cl

(pyrr)$_6$-CDP·2HCl (4·2HCl)·+ HpyrrCl

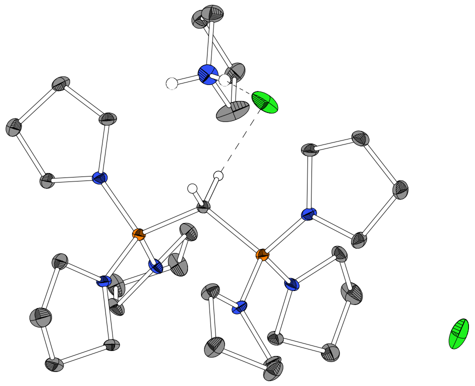

CCDC code	1903830
Crystal growth	Sebastian Ullrich
Solution and refinement	Sebastian Ullrich
Identification code	SU03900
Habitus, colour	block, clear colourless
Crystal size	0.330 x 0.220 x 0.140 mm^3
Crystal system	Orthorhombic
Space group	$P2_12_12$ $Z = 2$
Unit cell dimensions	
a = 11.5410(4) Å	α = 90°
b = 21.5098(8) Å	β = 90°
c = 8.3299(3) Å	γ = 90°
Volume	2067.85(13) Å^3
Cell determination	9440 peaks with Θ 2.6 to 27.1°
Empirical formula	$C_{33}H_{70}Cl_4N_8P_2$
Formula weight	782.71
Density (calculated)	1.257 g·cm^{-3}
Absorption coefficient	0.398 mm^{-1}
F(000)	844
Diffractometer type	Bruker D8 Quest
Wavelength	0.71073 Å
Temperature	100(2) K
Theta range for data collection	2.445 to 27.130°
Index ranges	
$-14 \le h \le 14$, $-27 \le k \le 25$, $-10 \le l \le 10$	
Reflections collected	27117
Independent reflections	4569 [R(int) = 0.0281]
Completeness to theta = 25.242°	99.6%
Observed reflections	4385 [$I > 2\sigma(I)$]
Reflections used for refinement	4569
Extinction coefficient	X = 0.0047(9)
Absorption correction	Semi-empirical from equivalents
Max. and min. transmission	0.746 and 0.672
Flack parameter (absolute struct.)	−0.21(6)
Largest diff. peak and hole	0.255 and -0.288 e·Å^{-3}
Solution	dual/difmap
Refinement	Full-matrix least-squares on F^2
Treatment of hydrogen atoms	mixed/hetero
Data / restraints / parameters	4569 / 0 / 223
Goodness-of-fit on F^2	1.082
R index (all data)	R_1 = 0.0285
	wR_2 = 0.0656
R index conventional [$I > 2\sigma(I)$]	R_1 = 0.0265
	wR_2 = 0.0646

Refinement special details
The asymmetric unit contains a half molecule completed by a twofold axis. Refined as a 2-component inversion twin.

(pyrr)$_6$-CDP·2HBF$_4$ (4·2HBF$_4$)

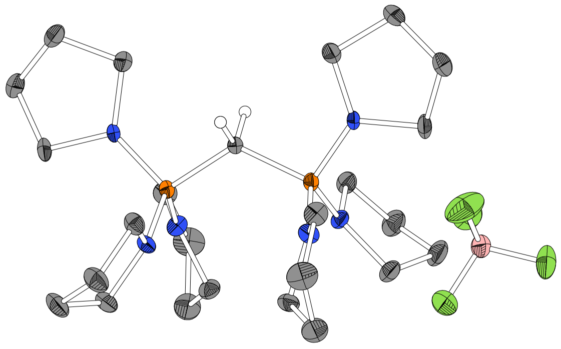

CCDC code	1903841
Crystal growth	Björn Koch
Solution and refinement	Sebastian Ullrich
Identification code	BK0400
Habitus, colour	prism, colourless
Crystal size	0.369 x 0.258 x 0.093 mm^3
Crystal system	Monoclinic
Space group	$C2/c$ $Z = 4$
Unit cell dimensions	
a = 19.6853(6) Å	α = 90°
b = 9.0107(2) Å	β = 108.791(2)°
c = 28.7299(10) Å	γ = 90°
Volume	4824.4(3) Å^3
Cell determination	17841 peaks with Θ 4.8 to 76.0°.
Empirical formula	$C_{29}H_{54}B_2Cl_{12}F_8N_6P_2$
Formula weight	1147.74
Density (calculated)	1.580 g·cm^{-3}
Absorption coefficient	7.494 mm^{-1}
F(000)	2344
Diffractometer type	Stoe Stadivari
Wavelength	1.54178 Å
Temperature	100(2) K
Theta range for data collection	4.746 to 75.728°
Index ranges	
$-24 \le h \le 23$, $-10 \le k \le 4$, $-35 \le l \le 35$	
Reflections collected	21625
Independent reflections	4801 [R(int) = 0.0593]
Completeness to theta = 70.000°	98.3%
Observed reflections	3360 [$I > 2\sigma(I)$]
Reflections used for refinement	4801
Absorption correction	Semi-empirical from equivalents
Max. and min. transmission	1.0000 and 0.2029
Largest diff. peak and hole	0.757 and −0.610 e·Å^{-3}
Solution	dual/difmap
Refinement	Full-matrix least-squares on F^2
Treatment of hydrogen atoms	geom/constr
Data / restraints / parameters	4801 / 0 / 267
Goodness-of-fit on F^2	0.925
R index (all data)	R_1 = 0.0672
	wR_2 = 0.1241
R index conventional [$I > 2\sigma(I)$]	R_1 = 0.0469
	wR_2 = 0.1182

Refinement special details
The asymmetric unit contains a half molecule completed by a twofold axis and two chloroform molecules.

$(pyrr)_6$-CDP (4)

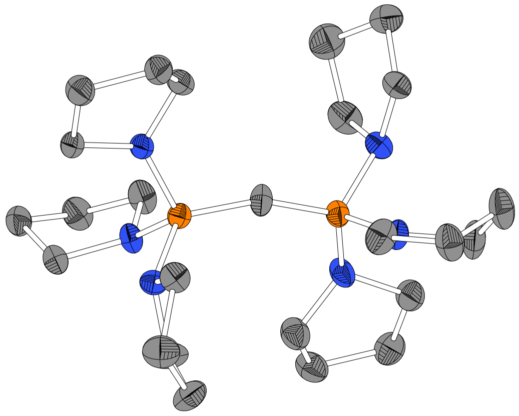

CCDC code	1903843	
Crystal growth	Björn Koch	
Solution and refinement	Sebastian Ullrich	
Identification code	BK1601	
Habitus, colour	block, colourless	
Crystal size	0.174 x 0.153 x 0.131 mm^3	
Crystal system	Monoclinic	
Space group	$P2_1/c$	Z = 4
Unit cell dimensions		
a = 10.8041(3) Å	α = 90°	
b = 14.3736(3) Å	β = 100.201(2)°	
c = 17.1480(5) Å	γ = 90°	
Volume	2620.88(12) $Å^3$	
Cell determination	20435 peaks with Θ 4.0 to 75.6°	
Empirical formula	$C_{25}H_{48}N_6P_2$	
Formula weight	494.63	
Density (calculated)	1.254 $g\cdot cm^{-3}$	
Absorption coefficient	1.691 mm^{-1}	
F(000)	1080	
Diffractometer type	Stoe Stadivari	
Wavelength	1.54178 Å	
Temperature	100(2) K	
Theta range for data collection	4.040 to 75.169°	
Index ranges		
$-6 \le h \le 13, -17 \le k \le 17, -21 \le l \le 21$		
Reflections collected	32319	
Independent reflections	5312 [R(int) = 0.0556]	
Completeness to theta = 70.000°	99.3%	
Observed reflections	3613 [$I > 2\sigma(I)$]	
Reflections used for refinement	5312	
Absorption correction	Semi-empirical from equivalents	
Max. and min. transmission	0.0332 and 0.0101	
Largest diff. peak and hole	0.633 and −0.288 $e\cdot Å^{-3}$	
Solution	dual/difmap	
Refinement	Full-matrix least-squares on F^2	
Treatment of hydrogen atoms	geom/constr	
Data / restraints / parameters	5312 / 30 / 337	
Goodness-of-fit on F^2	0.902	
R index (all data)	R_1 = 0.0658	
	wR_2 = 0.1126	
R index conventional [$I > 2\sigma(I)$]	R_1 = 0.0431	
	wR_2 = 0.1070	

Refinement special details

Three pyrrolidine rings were refined in 2-component disorder using RIGU restraints.

sym-$(tmg)_2(dma)_4$-CDP·2HBF_4 (1·2HBF_4)

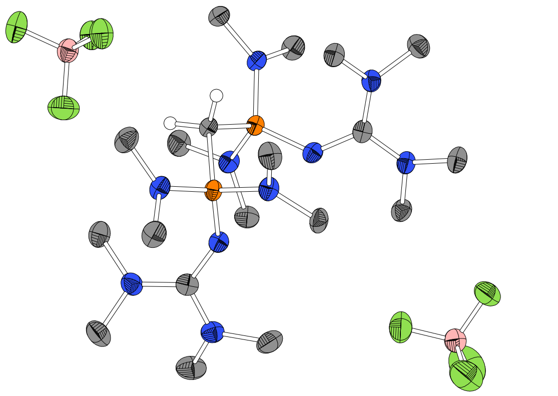

CCDC code	1903833	
Crystal growth	Björn Koch	
Solution and refinement	Sebastian Ullrich	
Identification code	BK14	
Habitus, colour	needle, colourless	
Crystal size	0.208 x 0.109 x 0.057 mm^3	
Crystal system	Monoclinic	
Space group	$P2_1/c$	Z = 4
Unit cell dimensions		
a = 12.0045(4) Å	α = 90°	
b = 25.5119(9) Å	β = 115.832(3)°	
c = 11.6750(4) Å	γ = 90°	
Volume	3218.3(2) $Å^3$	
Cell determination	27616 peaks with Θ 3.5 to 75.7°	
Empirical formula	$C_{19}H_{50}B_2F_8N_{10}P_2$	
Formula weight	654.25	
Density (calculated)	1.350 $g\cdot cm^{-3}$	
Absorption coefficient	1.901 mm^{-1}	
F(000)	1384	
Diffractometer type	Stoe Stadivari	
Wavelength	1.54178 Å	
Temperature	100(2) K	
Theta range for data collection	4.091 to 75.792°	
Index ranges		
$-15 \le h \le 12, -25 \le k \le 32, -14 \le l \le 14$		
Reflections collected	33222	
Independent reflections	6614 [R(int) = 0.0570]	
Completeness to theta = 70.000°	100.0%	
Observed reflections	4171 [$I > 2\sigma(I)$]	
Reflections used for refinement	6614	
Absorption correction	Semi-empirical from equivalents	
Max. and min. transmission	0.9990 and 0.1961	
Largest diff. peak and hole	0.532 and −0.419 $e\cdot Å^{-3}$	
Solution	dual/difmap	
Refinement	Full-matrix least-squares on F^2	
Treatment of hydrogen atoms	geom/constr	
Data / restraints / parameters	6614 / 0 / 386	
Goodness-of-fit on F^2	0.872	
R index (all data)	R_1 = 0.0734	
	wR_2 = 0.1259	
R index conventional [$I > 2\sigma(I)$]	R_1 = 0.0484	
	wR_2 = 0.1204	

sym-$(tmg)_2(dma)_4$-CDP (1)

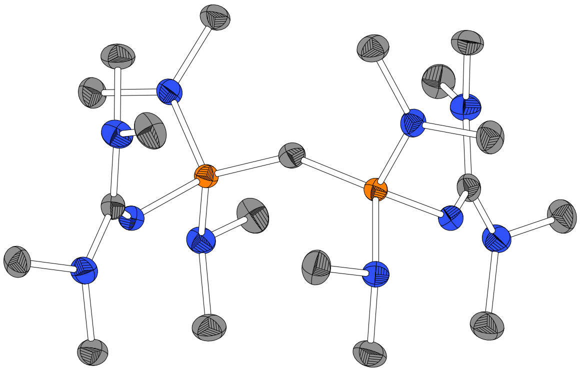

CCDC code	1903840	
Crystal growth	Björn Koch	
Solution and refinement	Sebastian Ullrich	
Identification code	BK23	
Habitus, colour	block, colourless	
Crystal size	0.230 x 0.180 x 0.153 mm^3	
Crystal system	Orthorhombic	
Space group	*Pbca*	$Z = 8$
Unit cell dimensions		
$a = 17.0571(2)$ Å	$\alpha = 90°$	
$b = 16.2524(3)$ Å	$\beta = 90°$	
$c = 19.3230(2)$ Å	$\gamma = 90°$	
Volume	5356.70(13) $Å^3$	
Cell determination	41617 peaks with Θ 3.5 to 75.9°	
Empirical formula	$C_{19}H_{48}N_{10}P_2$	
Formula weight	478.61	
Density (calculated)	1.187 $g·cm^{-3}$	
Absorption coefficient	1.677 mm^{-1}	
F(000)	2096	
Diffractometer type	Stoe Stadivari	
Wavelength	1.54178 Å	
Temperature	100(2) K	
Theta range for data collection	4.399 to 75.699°	
Index ranges		
$-15 \le h \le 21, -20 \le k \le 20, -22 \le l \le 24$		
Reflections collected	53455	
Independent reflections	5520 [*R*(int) = 0.0374]	
Completeness to theta = 70.000°	99.9%	
Observed reflections	4504 [$I > 2\sigma(I)$]	
Reflections used for refinement	5520	
Absorption correction	Semi-empirical from equivalents	
Max. and min. transmission	1.0000 and 0.5130	
Largest diff. peak and hole	0.319 and −0.342 $e·Å^{-3}$	
Solution	dual/difmap	
Refinement	Full-matrix least-squares on F^2	
Treatment of hydrogen atoms	geom/constr	
Data / restraints / parameters	5520 / 0 / 296	
Goodness-of-fit on F^2	1.059	
R index (all data)	$R_1 = 0.0425$	
	$wR_2 = 0.1024$	
R index conventional [$I > 2\sigma(I)$]	$R_1 = 0.0349$	
	$wR_2 = 0.0997$	

sym-$(dmaP_1)_2(dma)_4$-CDP·$2HBF_4$ (2·$2HBF_4$)

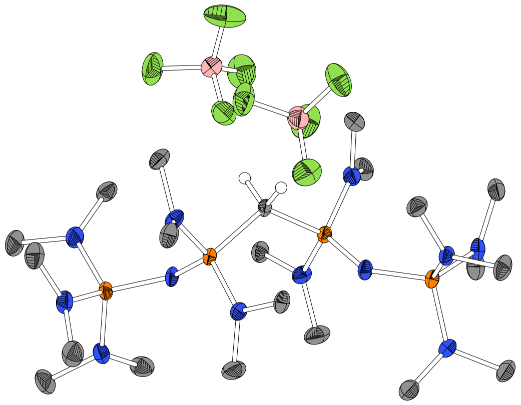

CCDC code	1903838	
Crystal growth	Björn Koch	
Solution and refinement	Klaus Harms	
Identification code	BK17	
Habitus, colour	needle, colourless	
Crystal size	0.56 x 0.07 x 0.07 mm^3	
Crystal system	Triclinic	
Space group	*P*1	$Z = 2$
Unit cell dimensions		
$a = 11.3316(5)$ Å	$\alpha = 94.444(4)°$	
$b = 11.7062(5)$ Å	$\beta = 93.147(4)°$	
$c = 14.3391(7)$ Å	$\gamma = 92.843(3)°$	
Volume	1890.73(15) $Å^3$	
Cell determination	59460 peaks with Θ 3.1 to 76.0°.	
Empirical formula	$C_{21}H_{62}B_2F_8N_{12}P_4$	
Formula weight	780.32	
Density (calculated)	1.371 $g·cm^{-3}$	
Absorption coefficient	2.495 mm^{-1}	
F(000)	828	
Diffractometer type	Stoe Stadivari	
Wavelength	1.54186 Å	
Temperature	100(2) K	
Theta range for data collection	3.097 to 74.933°	
Index ranges		
$-14 \le h \le 14, -14 \le k \le 14, -10 \le l \le 17$		
Reflections collected	33471	
Independent reflections	10558 [*R*(int) = 0.0454]	
Completeness to theta = 70.000°	98.6%	
Observed reflections	9844 [$I > 2\sigma(I)$]	
Reflections used for refinement	10558	
Absorption correction	Semi-empirical from equivalents	
Max. and min. transmission	0.6922 and 0.1438	
Flack parameter (absolute struct.)	0.48(2)	
Largest diff. peak and hole	0.556 and −0.457 $e·Å^{-3}$	
Solution	dual/difmap	
Refinement	Full-matrix least-squares on F^2	
Treatment of hydrogen atoms	geom/constr	
Data / restraints / parameters	10558 / 3 / 888	
Goodness-of-fit on F^2	1.027	
R index (all data)	$R_1 = 0.0485$	
	$wR_2 = 0.1214$	
R index conventional [$I > 2\sigma(I)$]	$R_1 = 0.0442$	
	$wR_2 = 0.1180$	

Refinement special details
The asymmetric unit containes two independent molecules. Refined as a 2-component inversion twin.

Computational Section

PA and GB calculation

Calculations in the gas phase are performed at the M06-2X/6-311+G(2df,p)//M06-2X/6-31+G(d) level of theory. All structures were optimized without any geometry constraints and confirmed to be an energy minimum on potential energy surface by computing their vibrational frequencies analytically.

Gas phase basicities (GB) have been calculated as the Gibbs free energy ΔG of the (gas phase) reaction: $B + H^+ \rightarrow BH^+$

Therefore, the gas basicity is calculated as: $GB = G(BH^+) - [G(B) + G(H^+)]$.

G of the neutral and protonated species contains the electronic energy E_{el} obtained at M06-2X/6-311+G(2df,p)//M06-2X/6-31+G(d)) level of theory and the thermal correction to free energy, G_{therm}, which sums the zero point vibrational energy (ZPVE), enthalpic and entropic contribution at 298 K.

Proton affinities (PA) in the gas phase are calculated as the enthalpy of the aforementioned reaction. $PA = H(BH^+) - [H(B) + H(H^+)]$

All structures were optimized and characterized as energy minima by the absence of imaginary frequencies. All calculations were performed with the Gaussian09 software.[11]

pK_a calculation

To calculate the pK_{BH^+} in THF we have used the isodesmic reaction approach (Scheme S1).

$$AH^{q}_{sol} + B^{q'-1}_{sol} \xrightarrow{\Delta G_{sol}} A^{q-1}_{sol} + BH^{q'}_{sol}$$

Scheme S1: Isodesmic reaction where proton exchange between an acidic species and a reference acid molecule. The charge of the acids and the conjugate bases are represented by q/q' and q−1/q'−1, respectively.

The pK_a values calculated by the following equation:

$$pK_a(AH^q) = \frac{\Delta G_{sol}}{RT \cdot \ln 10} + pK_a(BH^{q'})$$

pK_a ($BH^{q'}$) is experimentally known and the free energies of deprotonation in solution (ΔG_{sol}) are obtained by following equation:

$$\Delta G_{sol} = G_{sol}(A^{q-1}) + G_{sol}(BH^{q'}) - G_{sol}(AH^{q}) - G_{sol}(B^{q'-1})$$

The ΔG_{sol} values in this study were calculated using SMD/M06-2X/6-311+G(2df,p)//SMD/M06-2X/6-31+G(d) computational model in THF solvent.

Deprotonation/decomposition reaction

Reaction profile for deprotonation/decomposition reaction of **2·H⁺** in THF under the action of NH_2 is presented on Figure S36. Reaction profile is calculated utilizing SMD/M06-2X/6-311+G(2df,p)//SMD/M06-2X/6-31+G(d) computational model. Transition states are characterized by the presence of one imaginary frequency. The Intrinsic Reaction Coordinate (IRC) calculation has also been performed to confirm the smooth connection of the TS to the reactant and the product. Transition states TS1 and TS1ʻ correspond to proton transfer between **2·H⁺** and NH_2 . TS1 is the activation barrier for proton transfer between central C atom of **2·H⁺** whereas TS1ˊ is the activation barrier for proton transfer between peripheral NCH_3 group and NH_2 base. TS2 correspond to the activation barrier for P−N bond breaking with elimination of CH_2=N−CH_3 and formation of **7**.

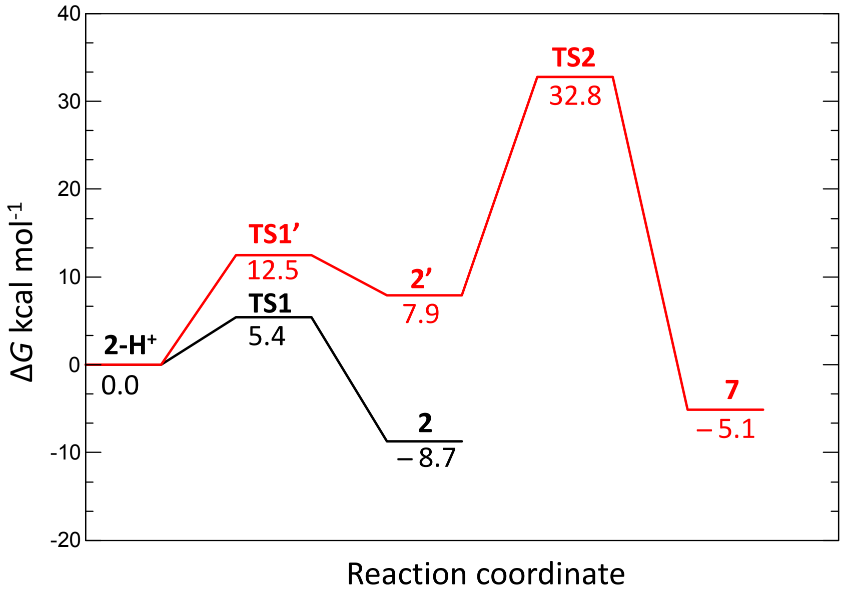

Figure S36. Relative energy profile for deprotonation/decomposition patway of **2·H⁺** in THF under the action of NH_2 calculated at SMD/M06-2X/6-311+G(2df,p)//SMD/M06-2X/6-31G(d) level of theory. Energy profile for deprotonation of central C atom is denoted by black line, whereas deprotonation of peripheral NCH_3 (TS1ʻ) together with elimination of N-methylmethaneimine (TS2) and formation of **7** is denoted by red.

Gas phase geometries of carbodiphosphoranes obtained by M06-2X/6-31+G(d) model

1

E(M062X) = -1982.43384847 a.u.

Atom	X	Y	Z
15	-0.826607	-1.277317	-0.112741
15	0.831414	1.300594	-0.053373
7	-2.362576	-1.075236	0.550183
7	-4.108709	0.298999	1.175898
7	-3.444227	0.362885	-1.039714
7	-0.972001	-2.420014	-1.356986
7	-0.191629	-2.320715	1.107064
7	2.357492	1.044698	0.612703
7	3.451589	-0.309844	-1.040881
7	4.087723	-0.372234	1.183024
7	0.192607	2.278930	1.220882
7	1.001413	2.504477	-1.232741
6	-0.007825	0.031369	-0.643791
6	-3.238781	-0.184545	0.209069
6	-3.813954	-0.018865	2.559350
1	-4.430026	0.621582	3.197989
1	-2.761291	0.180013	2.765951
1	-4.021364	-1.073201	2.791976
6	-5.525628	0.465285	0.891338
1	-5.888978	1.423749	1.281095
1	-6.104349	-0.341762	1.363184
1	-5.702959	0.430446	-0.183565
6	-3.758518	1.768343	-1.225641
1	-4.662030	1.889476	-1.839111
1	-2.921790	2.265169	-1.736204
1	-3.913978	2.254191	-0.262066
6	-3.033312	-0.321815	-2.249947
1	-3.735708	-0.050091	-3.046807
1	-3.070035	-1.400829	-2.095553
1	-2.013658	-0.044981	-2.549060
6	-1.667937	-3.666768	-1.125638
1	-2.389670	-3.858244	-1.935370
1	-2.208718	-3.624806	-0.178405
1	-0.968267	-4.517774	-1.087456
6	-0.310530	-2.313023	-2.631396
1	-1.038256	-2.377613	-3.457660
1	0.423727	-3.125084	-2.770607
1	0.206549	-1.351812	-2.685967
6	1.145949	-2.803578	0.810033
1	1.360185	-3.682345	1.431621
1	1.918700	-2.040292	1.009405
1	1.214610	-3.097914	-0.242329
6	-0.316310	-1.949303	2.504451
1	-0.150549	-2.839216	3.125265
1	-1.322114	-1.572604	2.695077
1	0.423164	-1.186295	2.804358
6	3.232952	0.169609	0.233318
6	3.056780	0.439488	-2.217670
1	3.758637	0.195373	-3.023825
1	3.110324	1.509510	-2.013287
1	2.034984	0.193142	-2.535313
6	3.769282	-1.703263	-1.298330
1	4.697697	-1.795161	-1.879006

1	2.953242	-2.161167	-1.874164
1	3.879868	-2.247490	-0.360029
6	5.508336	-0.524758	0.909381
1	5.866191	-1.502413	1.253750
1	6.080893	0.256552	1.429562
1	5.700644	-0.434395	-0.159612
6	3.774716	-0.128981	2.577632
1	4.376750	-0.808500	3.188589
1	2.717352	-0.330320	2.756759
1	3.985520	0.909371	2.870706
6	0.233390	1.726754	2.563737
1	-0.023166	2.515022	3.282192
1	1.240429	1.367798	2.781984
1	-0.484921	0.898206	2.692709
6	-1.132579	2.798096	0.920951
1	-1.377774	3.592974	1.636027
1	-1.910223	2.016882	0.987780
1	-1.152596	3.221478	-0.088459
6	1.718932	3.724325	-0.933040
1	2.455187	3.939058	-1.723584
1	2.244956	3.624395	0.018232
1	1.036066	4.586715	-0.863044
6	0.346613	2.472831	-2.514988
1	-0.385805	3.292550	-2.612070
1	-0.171172	1.517060	-2.628193
1	1.080371	2.583622	-3.330717

1-H

E(M062X) = -1982.91290677 a. u.

Atom	X	Y	Z
6	0.017248	0.067922	-0.877419
15	-0.864821	-1.213669	-0.169425
7	-0.912253	-2.446381	-1.320478
6	0.265977	-2.806248	-2.101152
7	-2.405800	-1.036349	0.357986
6	-3.305350	-0.138017	0.035747
7	-4.259715	0.175008	0.962534
6	-4.080134	-0.268833	2.336351
7	-0.068171	-1.677857	1.249065
6	1.247009	-2.282242	1.046467
6	-5.646425	0.434788	0.584261
6	-0.777309	-2.317134	2.355742
6	-1.819428	-3.576043	-1.126731
15	0.867523	1.264708	0.016790
7	0.095044	1.749307	1.422180
6	0.395299	1.250642	2.753888
7	2.386738	0.972851	0.568152
6	3.311087	0.164218	0.112956
7	3.413236	-0.325748	-1.166137
6	3.048251	0.448615	-2.340951
7	0.964741	2.612841	-0.984338
6	-0.066146	2.993076	-1.939113
6	4.005872	-1.621813	-1.462517
6	1.723414	3.758962	-0.485739
6	-1.251732	2.283218	1.293395
7	4.273589	-0.270062	0.983921
6	5.675028	-0.368081	0.580654
6	4.069050	-0.065292	2.409568

7	-3.389051	0.532930	-1.153999
6	-3.881910	1.898046	-1.253387
6	-2.987412	-0.062829	-2.416865
1	4.788021	-0.686167	2.949699
1	3.060654	-0.371251	2.687409
1	4.209167	0.985640	2.692097
1	6.057369	-1.387932	0.698985
1	6.272082	0.303683	1.206512
1	5.796503	-0.057162	-0.457010
1	4.991557	-1.530488	-1.936294
1	3.346409	-2.153709	-2.158246
1	4.100803	-2.211768	-0.549968
1	3.926859	0.552755	-2.990249
1	2.696902	1.438788	-2.053290
1	2.258099	-0.055371	-2.911778
1	2.164346	4.294628	-1.333828
1	2.525170	3.423274	0.173989
1	1.075053	4.453115	0.068103
1	0.401016	3.330825	-2.872246
1	-0.682161	3.819230	-1.551746
1	-0.717063	2.144881	-2.156508
1	-1.363137	3.161494	1.940648
1	-2.008274	1.539594	1.581179
1	-1.452823	2.585807	0.262150
1	0.239749	2.058144	3.478787
1	1.436611	0.938179	2.799443
1	-0.252952	0.403005	3.019762
1	-3.807652	0.048748	-3.136092
1	-2.771604	-1.122700	-2.285582
1	-2.096779	0.428960	-2.825173
1	-4.846256	1.951470	-1.774256
1	-3.155624	2.490903	-1.822366
1	-3.985991	2.335049	-0.259380
1	-5.963810	1.439166	0.885314
1	-6.292485	-0.298113	1.079384
1	-5.774656	0.324916	-0.492590
1	-4.743764	0.318904	2.975282
1	-3.048488	-0.105730	2.651444
1	-4.318539	-1.334313	2.448869
1	-0.918798	-3.394862	2.183224
1	-1.751342	-1.855108	2.495603
1	-0.181524	-2.193082	3.267415
1	1.167683	-3.367953	0.876629
1	1.865526	-2.121336	1.936874
1	1.747215	-1.827440	0.188479
1	-2.138073	-3.953550	-2.105364
1	-2.701130	-3.262539	-0.566018
1	-1.327140	-4.398537	-0.587033
1	0.797115	-3.663534	-1.660889
1	0.954606	-1.960952	-2.157550
1	-0.041071	-3.080372	-3.117279
1	0.079238	0.093716	-1.960161

1-2H
E(M062X) = -1983.24552659 a.u.

Atom	X	Y	Z
15	1.151751	-0.687821	1.019690
15	-1.139961	-0.714333	-0.987773

7	1.618204	0.598121	0.197144
7	3.202133	2.189452	-0.282177
7	3.612121	0.009059	-0.956629
7	2.364273	-1.716266	1.483634
7	0.371227	-0.129772	2.369065
7	-1.612628	0.583786	-0.188146
7	-3.181690	2.206237	0.234803
7	-3.649877	0.036114	0.906834
7	-2.343532	-1.758544	-1.434909
7	-0.346219	-0.183028	-2.339979
6	2.798362	0.905102	-0.336130
6	2.559256	3.112495	0.647425
1	3.254575	3.927843	0.857440
1	2.318905	2.597972	1.578011
1	1.638978	3.526625	0.217805
6	0.007168	-1.725614	0.026549
1	-0.559238	-2.362470	0.716600
1	0.579745	-2.370878	-0.649990
6	4.031247	2.812623	-1.314785
1	5.030103	3.055112	-0.939102
1	3.544000	3.738980	-1.632274
1	4.116694	2.153676	-2.178827
6	5.070856	0.092611	-0.899401
1	5.458411	-0.881903	-0.584138
1	5.375449	0.839657	-0.166068
1	5.506294	0.338219	-1.873708
6	3.088099	-1.179372	-1.609588
1	3.610611	-1.313253	-2.562462
1	2.023964	-1.043530	-1.815830
1	3.239609	-2.077698	-0.998483
6	3.422318	-1.139805	2.322750
1	4.389405	-1.544620	2.007614
1	3.263837	-1.385165	3.380563
1	3.451445	-0.051366	2.219151
6	2.310354	-3.176700	1.550188
1	3.262520	-3.586420	1.197463
1	1.515937	-3.579780	0.921897
1	2.152007	-3.521005	2.580274
6	0.155302	-1.001191	3.523285
1	-0.916038	-1.123198	3.724223
1	0.622553	-0.564942	4.413182
1	0.590603	-1.989733	3.357644
6	-0.193953	1.209807	2.511477
1	-1.277686	1.143768	2.682850
1	-0.015656	1.786120	1.604739
1	0.263404	1.711874	3.371963
6	-2.802534	0.915343	0.307277
6	-2.495822	3.111130	-0.681794
1	-3.157417	3.954818	-0.888252
1	-2.270869	2.597755	-1.616848
1	-1.562693	3.484290	-0.242960
6	-4.022653	2.851426	1.244310
1	-3.517191	3.761161	1.581129
1	-4.154438	2.193024	2.102615
1	-5.001110	3.125456	0.838089
6	-5.104744	0.151298	0.820262
1	-5.507151	-0.817191	0.505097
1	-5.377737	0.899401	0.075377

1	-5.554575	0.413750	1.783520
6	-3.165113	-1.153189	1.587757
1	-3.687083	-1.248278	2.545783
1	-2.095854	-1.047448	1.784455
1	-3.346059	-2.059749	0.997428
6	-2.281203	-3.219164	-1.481434
1	-3.215663	-3.632835	-1.087872
1	-1.458954	-3.607616	-0.880119
1	-2.158617	-3.574061	-2.512644
6	-3.426855	-1.200093	-2.252685
1	-4.388064	-1.567702	-1.877958
1	-3.319424	-1.499482	-3.302689
1	-3.429315	-0.107259	-2.201637
6	0.183336	1.166771	-2.519484
1	-0.314593	1.652411	-3.367082
1	1.260697	1.117871	-2.732434
1	0.030114	1.750201	-1.612375
6	-0.174853	-1.060485	-3.497795
1	-0.758465	-0.688545	-4.348067
1	-0.501862	-2.078924	-3.273089
1	0.879547	-1.096682	-3.795332

2

E(M062X) = -2857.96854061 a.u.

Atom	X	Y	Z
6	-0.009920	0.006007	-0.543428
15	1.213230	-0.974662	-0.114895
7	2.604287	-0.323963	0.468850
15	4.052829	0.161238	0.261331
7	4.614041	0.730540	1.747379
6	3.646999	1.252929	2.704987
7	0.854647	-2.149544	1.059895
6	1.845160	-3.128908	1.448721
7	1.755440	-1.946736	-1.458249
6	0.782059	-2.959821	-1.850901
7	5.298412	-0.898206	-0.159902
6	5.282354	-1.458852	-1.511719
7	4.180637	1.304730	-0.965605
6	3.030420	2.145877	-1.280001
6	-0.510325	-2.521969	1.353571
6	2.106392	-1.143306	-2.626213
6	5.685735	-1.907793	0.823490
6	5.446867	1.851445	-1.426697
6	5.891342	1.426641	1.790604
15	-1.222395	0.999038	-0.111753
7	-2.632892	0.360067	0.432595
15	-4.063937	-0.175606	0.246136
7	-4.568680	-0.834953	1.711582
6	-5.811942	-1.587987	1.758674
7	-0.866715	2.148773	1.088432
6	-1.838541	3.161925	1.437589
7	-1.732032	2.007472	-1.438916
6	-2.131747	1.237196	-2.612784
7	-5.348572	0.868436	-0.096636
6	-5.303563	1.541073	-1.397332
7	-4.193425	-1.258137	-1.036057
6	-3.043908	-2.099693	-1.353621
6	0.496613	2.480140	1.434741

6	-0.716433	2.980163	-1.826436
6	-5.757781	1.791670	0.960284
6	-5.459664	-1.804143	-1.499408
6	-3.579687	-1.286543	2.680283
1	0.838418	3.412846	0.948629
1	1.161349	1.665418	1.137762
1	0.579062	2.622909	2.521869
1	-1.775526	3.385567	2.511470
1	-2.843362	2.797381	1.216193
1	-1.677796	4.102483	0.882181
1	-1.140177	3.659958	-2.575441
1	0.174830	2.493151	-2.259799
1	-0.405566	3.574041	-0.962364
1	-2.601892	1.909277	-3.342938
1	-2.857777	0.467117	-2.339103
1	-1.268246	0.739585	-3.084850
1	-6.781770	2.125701	0.757935
1	-5.108640	2.678600	1.000541
1	-5.730701	1.291773	1.929276
1	-6.267865	2.032894	-1.564904
1	-5.141122	0.816222	-2.197417
1	-4.501185	2.293402	-1.435154
1	-5.666703	-2.791420	-1.059379
1	-5.421973	-1.922756	-2.590198
1	-6.279873	-1.127545	-1.252143
1	-3.091086	-3.066888	-0.828045
1	-2.109901	-1.592658	-1.091980
1	-3.034791	-2.296154	-2.433522
1	-4.015624	-1.223939	3.684637
1	-2.698782	-0.645661	2.628987
1	-3.275801	-2.330145	2.500283
1	-6.210335	-1.558937	2.779886
1	-5.668539	-2.643817	1.478783
1	-6.553337	-1.142048	1.091017
1	-0.808522	-3.460712	0.851334
1	-1.183391	-1.722745	1.033879
1	-0.628833	-2.671055	2.436435
1	1.770969	-4.059548	0.859261
1	1.715540	-3.386671	2.508634
1	2.844276	-2.710572	1.314256
1	2.595544	-1.783944	-3.371855
1	2.802348	-0.346120	-2.350971
1	1.215846	-0.676887	-3.078660
1	1.225891	-3.601335	-2.621561
1	-0.139700	-2.509279	-2.259619
1	0.516701	-3.587084	-0.995899
1	6.722420	-2.208413	0.631652
1	5.050386	-2.802786	0.752748
1	5.614506	-1.501215	1.833330
1	6.262448	-1.907183	-1.709826
1	5.103888	-0.674850	-2.249914
1	4.504472	-2.228969	-1.618571
1	5.414930	1.969681	-2.518155
1	6.268357	1.177147	-1.177331
1	5.649597	2.839025	-0.986478
1	3.022599	2.349434	-2.359045
1	3.075876	3.109064	-0.748015
1	2.097144	1.635812	-1.022844

1	6.285816	1.385357	2.812527
1	5.798130	2.486528	1.503785
1	6.611153	0.941898	1.126418
1	4.091123	1.214254	3.706354
1	2.745527	0.640142	2.685841
1	3.375135	2.298909	2.487801

2-H

E(M062X) = -2858.46251891 a.u.

Atom	X	Y	Z
6	-0.009927	0.519377	-1.510700
15	1.280378	-0.465766	-0.954491
7	2.295629	0.210188	0.096467
15	3.798784	0.071339	0.543182
7	3.786356	-0.248406	2.191610
6	2.688716	0.276709	3.001767
7	0.637414	-1.834161	-0.234380
6	1.256959	-2.552006	0.866587
7	2.230831	-0.929967	-2.297584
6	1.712951	-1.985957	-3.162418
7	4.827451	-1.111045	-0.028058
6	5.410273	-1.013593	-1.365690
7	4.608751	1.467619	0.111405
6	3.899383	2.702827	-0.193577
6	-0.476870	-2.564653	-0.824802
6	2.784018	0.178577	-3.073513
6	4.670304	-2.501544	0.391070
6	6.010917	1.672469	0.461741
6	5.043274	-0.333791	2.932135
15	-1.324511	1.029277	-0.534128
7	-2.305854	-0.142483	-0.055033
15	-3.765380	-0.467908	0.419614
7	-3.752249	-0.826540	2.048535
6	-4.940639	-0.900659	2.892041
7	-0.742680	1.801663	0.838750
6	-1.507423	1.943711	2.066812
7	-2.263463	2.146778	-1.426500
6	-2.703223	1.684731	-2.741768
7	-4.980985	0.679192	0.331015
6	-5.615995	0.937375	-0.959241
7	-4.285404	-1.782551	-0.465172
6	-3.821456	-2.051868	-1.818553
6	0.387715	2.717737	0.768079
6	-1.792766	3.529344	-1.472350
6	-4.869274	1.896357	1.134188
6	-5.547120	-2.432670	-0.134962
6	-2.608996	-1.558206	2.586932
1	0.079193	0.953113	-2.502832
1	0.069669	3.748112	0.982504
1	0.832223	2.679597	-0.226367
1	1.154238	2.427542	1.496832
1	-0.817645	1.903030	2.918846
1	-2.225862	1.130203	2.170507
1	-2.041965	2.905376	2.108735
1	-2.571220	4.145889	-1.933241
1	-0.868242	3.646848	-2.060947
1	-1.621828	3.904509	-0.461498
1	-3.515591	2.328679	-3.096208

1	-3.079273	0.659863	-2.678568
1	-1.895280	1.714792	-3.489110
1	-5.864772	2.341923	1.233378
1	-4.199274	2.625756	0.655541
1	-4.495167	1.665487	2.133503
1	-6.655113	1.239597	-0.790734
1	-5.615697	0.038288	-1.578975
1	-5.097682	1.741295	-1.497438
1	-5.460464	-3.505938	-0.334718
1	-6.380949	-2.035272	-0.732317
1	-5.786624	-2.303050	0.921309
1	-3.628187	-3.125381	-1.930384
1	-2.895319	-1.507390	-2.003632
1	-4.572989	-1.758704	-2.567509
1	-2.437260	-1.238820	3.621197
1	-1.716043	-1.335803	1.998086
1	-2.788244	-2.642739	2.581523
1	-4.739810	-0.394888	3.844336
1	-5.207892	-1.943564	3.111277
1	-5.785751	-0.410683	2.406102
1	-0.161479	-3.578907	-1.108376
1	-0.837260	-2.039955	-1.709329
1	-1.306080	-2.636352	-0.109753
1	1.737782	-3.479711	0.524224
1	0.483045	-2.823635	1.595967
1	1.997225	-1.929862	1.369619
1	3.601029	-0.194101	-3.701358
1	3.187271	0.946052	-2.405826
1	2.033728	0.642299	-3.733613
1	2.508095	-2.290817	-3.851205
1	0.846186	-1.660571	-3.760044
1	1.432971	-2.857803	-2.568380
1	5.651265	-2.988994	0.366342
1	3.996470	-3.041681	-0.289284
1	4.277387	-2.557496	1.407047
1	6.417483	-1.444849	-1.344765
1	5.480719	0.028613	-1.680206
1	4.797779	-1.564389	-2.091800
1	6.517403	2.186530	-0.363687
1	6.510604	0.716002	0.625487
1	6.114562	2.289568	1.364693
1	4.443221	3.241382	-0.978380
1	3.824779	3.358039	0.686425
1	2.896550	2.470266	-0.549919
1	4.890650	-0.964730	3.814181
1	5.388149	0.653432	3.272007
1	5.821569	-0.788352	2.316081
1	2.595446	-0.337509	3.903751
1	1.755472	0.230951	2.437411
1	2.872240	1.317161	3.310779

2-H
E(M062X) = -2858.81136932 a.u.

Atom	X	Y	Z
15	1.340603	-0.804502	-0.742872
15	3.758046	0.320477	0.495126
15	-1.375461	0.885171	-0.650329
15	-3.776338	-0.340270	0.426267

7	0.633589	-1.940051	0.228650
7	2.210568	-1.567895	-1.961468
7	2.211656	0.245503	0.055337
7	4.809538	-0.837954	-0.048273
7	4.291260	1.808299	0.001048
7	3.848593	0.154260	2.143244
7	-0.761553	1.893585	0.503325
7	-2.212423	1.835524	-1.741308
7	-2.251985	-0.306529	-0.089498
7	-4.720502	1.016503	0.360471
7	-4.585010	-1.439895	-0.514600
7	-3.709908	-0.671724	2.059921
6	0.001953	0.119285	-1.598408
1	0.503543	0.916153	-2.164010
1	-0.459488	-0.539203	-2.346673
6	-0.391699	-2.853317	-0.273252
1	-0.010375	-3.880806	-0.312445
1	-0.712969	-2.565314	-1.276734
1	-1.268460	-2.818171	0.379776
6	1.230602	-2.354647	1.497555
1	0.432180	-2.503625	2.232203
1	1.908829	-1.583738	1.869533
1	1.779778	-3.299205	1.387363
6	2.060947	-2.994590	-2.252256
1	2.973502	-3.342080	-2.746530
1	1.210854	-3.205719	-2.916374
1	1.946328	-3.560357	-1.326483
6	2.565254	-0.763729	-3.133303
1	3.402405	-1.236314	-3.653916
1	2.885045	0.238225	-2.828280
1	1.732528	-0.676072	-3.846160
6	4.688045	-2.225450	0.396608
1	5.686411	-2.671064	0.459797
1	4.087940	-2.809249	-0.314807
1	4.226255	-2.275433	1.384594
6	5.501506	-0.707736	-1.327614
1	6.544535	-1.019023	-1.209941
1	5.486649	0.326779	-1.676804
1	5.029395	-1.346016	-2.084275
6	5.609227	2.275683	0.436056
1	5.560954	3.349976	0.636954
1	6.372981	2.096576	-0.331966
1	5.915010	1.769988	1.353163
6	3.772886	2.513319	-1.165359
1	3.726194	3.584680	-0.943454
1	2.766183	2.159944	-1.392835
1	4.417948	2.373737	-2.044469
6	2.882929	0.881645	2.965986
1	2.721021	0.328562	3.896763
1	1.934211	0.961686	2.434326
1	3.245928	1.887841	3.214429
6	5.086906	-0.160091	2.864659
1	4.869972	-0.902924	3.639669
1	5.491012	0.735993	3.352003
1	5.839031	-0.565783	2.187446
6	0.300807	2.854192	0.205418
1	0.016438	3.845761	0.575721
1	0.465943	2.933712	-0.872741

1	1.240946	2.540564	0.672369
6	-1.010305	1.705268	1.930590
1	-1.232914	2.676178	2.386901
1	-0.130337	1.275688	2.422878
1	-1.859685	1.042012	2.093752
6	-2.640454	1.196692	-2.988136
1	-3.535076	1.705328	-3.359642
1	-2.903583	0.149215	-2.808761
1	-1.867549	1.249727	-3.767825
6	-2.082490	3.289659	-1.834962
1	-3.017002	3.693243	-2.237081
1	-1.265982	3.594598	-2.504477
1	-1.926144	3.722074	-0.846323
6	-4.472277	2.140458	1.265875
1	-5.407413	2.690981	1.404993
1	-3.723153	2.826727	0.843815
1	-4.129984	1.787503	2.239198
6	-5.394175	1.428684	-0.872756
1	-6.403486	1.776571	-0.629692
1	-5.467279	0.594328	-1.571526
1	-4.844398	2.249850	-1.349101
6	-5.977022	-1.779747	-0.200487
1	-6.520897	-1.949457	-1.135205
1	-6.463741	-0.960790	0.332727
1	-6.037067	-2.690452	0.408363
6	-3.908119	-2.452755	-1.318914
1	-4.495342	-2.632882	-2.225318
1	-3.811351	-3.402760	-0.776048
1	-2.917337	-2.099175	-1.602737
6	-4.947711	-0.765155	2.844226
1	-4.728444	-0.482617	3.878410
1	-5.352296	-1.785476	2.842297
1	-5.702361	-0.080623	2.453019
6	-2.650298	-1.542070	2.571139
1	-2.515939	-1.335817	3.637821
1	-1.714176	-1.332478	2.049323
1	-2.904313	-2.605711	2.456243

4

E(M062X) = -1992.11391788 a.u.

Atom	X	Y	Z
6	4.054924	-0.321342	1.218197
7	2.615239	-0.420106	1.444015
6	2.292560	-0.723916	2.838867
6	3.598415	-0.401756	3.568926
6	4.662281	-0.802700	2.542064
15	1.479369	-0.135220	0.240786
6	-0.006297	-0.019503	0.909850
15	-1.490314	0.137915	0.243683
7	-2.039607	-1.157292	-0.702502
6	-3.145031	-1.131265	-1.672722
6	-3.743100	-2.539742	-1.594061
6	-2.524561	-3.395526	-1.238556
6	-1.803417	-2.520043	-0.213124
7	1.893697	-1.353725	-0.884619
6	1.155837	-1.428756	-2.151328
6	1.661351	-2.739395	-2.747870
6	1.708631	-3.657055	-1.519070

6	2.081843	-2.717450	-0.352647
7	2.028758	1.206911	-0.637904
6	3.137658	1.231764	-1.603514
6	3.744227	2.629398	-1.440952
6	2.531915	3.470990	-1.032197
6	1.802287	2.538607	-0.065346
7	-1.893483	1.410003	-0.825075
6	-2.080918	2.749052	-0.232805
6	-1.675823	3.739050	-1.345261
6	-1.620191	2.881781	-2.616619
6	-1.137380	1.537972	-2.076405
7	-2.628995	0.370827	1.456554
6	-2.310437	0.577337	2.870671
6	-3.599088	0.152948	3.577169
6	-4.681620	0.597755	2.588927
6	-4.066542	0.239346	1.230506
1	-2.086134	1.636614	3.075858
1	-1.433277	-0.010510	3.150474
1	-3.707401	0.600684	4.569151
1	-3.618612	-0.938501	3.686559
1	-5.651964	0.119679	2.750763
1	-4.820169	1.683820	2.656965
1	-4.324957	-0.792701	0.941504
1	-4.412861	0.904653	0.427367
1	-2.758510	-0.944512	-2.686832
1	-3.868051	-0.341788	-1.450601
1	-4.237943	-2.839114	-2.522646
1	-4.478989	-2.590993	-0.781782
1	-2.778295	-4.384946	-0.847764
1	-1.890028	-3.526471	-2.125538
1	-2.229011	-2.663148	0.794410
1	-0.729873	-2.723397	-0.144999
1	-1.466457	2.888807	0.668174
1	-3.129385	2.873225	0.066301
1	-2.370680	4.579771	-1.424729
1	-0.682090	4.152860	-1.137790
1	-2.621866	2.766689	-3.046585
1	-0.958500	3.295855	-3.383568
1	-1.357792	0.699078	-2.744984
1	-0.048316	1.553110	-1.899686
1	2.028662	-1.786417	2.963680
1	1.438658	-0.127225	3.168277
1	3.693491	-0.931975	4.520799
1	3.656605	0.675802	3.765867
1	5.648511	-0.370885	2.735065
1	4.765070	-1.894746	2.524245
1	4.347878	0.719627	1.002409
1	4.373819	-0.936898	0.365572
1	2.753435	1.107702	-2.627992
1	3.855749	0.426877	-1.424763
1	4.239126	2.982385	-2.350455
1	4.481743	2.626881	-0.628515
1	2.794424	4.430660	-0.578066
1	1.899898	3.665862	-1.909202
1	2.226468	2.614555	0.949952
1	0.729790	2.743989	0.013601
1	1.451601	-2.906906	0.528233
1	3.125591	-2.846844	-0.038709

1	2.417261	-4.482753	-1.628209
1	0.717801	-4.094174	-1.348992
1	2.667707	-2.592563	-3.156702
1	1.016418	-3.123103	-3.544231
1	1.377021	-0.557518	-2.776688
1	0.064209	-1.463162	-1.991718

4-H

E(M062X) = -1992.58805742 a.u.

Atom	X	Y	Z
6	-1.326801	1.913101	-1.780643
7	-2.003294	1.468723	-0.546596
6	-2.340791	2.644048	0.292673
6	-1.998532	3.861987	-0.583493
6	-1.933163	3.295609	-2.005121
15	-1.570043	0.047794	0.229241
7	-1.671722	-1.061644	-1.009132
6	-2.700844	-1.009784	-2.077113
6	-3.256450	-2.431457	-2.133968
6	-2.017907	-3.268989	-1.813352
6	-1.355913	-2.475248	-0.685774
6	-0.063261	-0.106851	1.029300
15	1.480570	-0.045220	0.282863
7	1.787240	-1.043900	-1.007144
6	2.319371	-2.407238	-0.789039
6	2.158722	-3.087526	-2.156251
6	2.138742	-1.917935	-3.145270
6	1.318334	-0.872643	-2.392855
7	2.562135	-0.439586	1.475808
6	2.307862	-1.329115	2.625667
6	3.624006	-1.265597	3.401033
6	4.668270	-1.150605	2.288832
6	4.006773	-0.195119	1.290675
7	1.848522	1.458537	-0.334756
6	2.938764	1.777098	-1.282845
6	3.498261	3.103311	-0.769128
6	2.245106	3.787483	-0.217003
6	1.522386	2.641619	0.491518
7	-2.735723	-0.113067	1.393550
6	-2.516618	-0.508500	2.800122
6	-3.905939	-0.957291	3.266701
6	-4.856777	-0.128249	2.399397
6	-4.165595	-0.156963	1.037461
1	-0.067026	0.182103	2.077240
1	2.094262	-2.354980	2.294833
1	1.456357	-0.978445	3.212821
1	3.769001	-2.137681	4.042662
1	3.641863	-0.368421	4.029845
1	5.637061	-0.783607	2.634786
1	4.822088	-2.129543	1.819817
1	4.243447	0.853524	1.516594
1	4.319175	-0.399660	0.259504
1	2.522324	1.915192	-2.290261
1	3.678639	0.974121	-1.333775
1	3.999088	3.676054	-1.553293
1	4.217417	2.925191	0.038954
1	2.459074	4.621106	0.455832
1	1.630557	4.164083	-1.044643

1	1.900724	2.516578	1.516992
1	0.438146	2.779750	0.548133
1	1.776196	-2.938794	0.002053
1	3.373267	-2.351019	-0.488616
1	2.954829	-3.809562	-2.350880
1	1.202927	-3.623016	-2.199503
1	3.154635	-1.543463	-3.316067
1	1.700052	-2.179665	-4.111380
1	1.493035	0.148612	-2.740459
1	0.242772	-1.082168	-2.472864
1	-2.164974	0.353914	3.382350
1	-1.770245	-1.304744	2.876803
1	-4.049609	-0.803367	4.338534
1	-4.043942	-2.023671	3.054593
1	-5.871806	-0.530064	2.361208
1	-4.905712	0.903079	2.767539
1	-4.403606	-1.088222	0.501323
1	-4.435909	0.685808	0.393356
1	-2.219966	-0.761556	-3.033338
1	-3.452524	-0.244643	-1.866952
1	-3.703208	-2.664145	-3.103559
1	-4.020552	-2.574977	-1.360717
1	-2.243378	-4.296126	-1.517211
1	-1.357340	-3.303196	-2.689184
1	-1.783860	-2.751255	0.288912
1	-0.275148	-2.622118	-0.632228
1	-1.775018	2.647526	1.234303
1	-3.405173	2.614221	0.550933
1	-2.733125	4.662851	-0.472803
1	-1.021403	4.270211	-0.301751
1	-2.939872	3.191809	-2.424433
1	-1.338317	3.910081	-2.685706
1	-1.525624	1.220332	-2.601849
1	-0.235658	1.979052	-1.641263

4-2H
E(M062X) = -1992.90426853 a.u.

Atom	X	Y	Z
15	-1.589287	-0.060277	0.241383
7	-2.688566	-0.085334	1.454488
7	-1.576685	-1.420199	-0.677832
7	-1.883789	1.204287	-0.750262
6	-0.000008	-0.000011	1.146479
1	0.034418	-0.880699	1.802239
6	-2.549482	0.457324	2.831338
1	-2.390931	1.543632	2.806533
1	-1.713287	-0.013808	3.355366
6	-3.897347	0.116655	3.470837
1	-4.134981	0.787132	4.298917
1	-3.878080	-0.908760	3.854564
6	-4.877471	0.226711	2.301611
1	-5.830408	-0.271719	2.488851
1	-5.080751	1.279446	2.074855
6	-4.108071	-0.418484	1.148398
1	-4.230600	-1.508217	1.139700
1	-4.406101	-0.026713	0.169100
6	-2.555854	-1.682877	-1.773907
1	-2.033072	-1.632223	-2.736938

1	-3.357306	-0.939226	-1.778105
6	-3.027027	-3.108447	-1.501078
1	-3.424303	-3.588320	-2.397898
1	-3.809606	-3.112655	-0.734040
6	-1.755288	-3.773379	-0.972116
1	-1.937784	-4.713213	-0.447453
1	-1.065874	-3.973834	-1.800990
6	-1.166391	-2.706224	-0.044826
1	-1.594789	-2.788761	0.963945
1	-0.074522	-2.762373	0.026881
6	-2.647470	2.398091	-0.282814
1	-2.218748	2.816010	0.634904
1	-3.685802	2.116050	-0.077657
6	-2.551018	3.366983	-1.467375
1	-3.429217	4.011887	-1.534201
1	-1.669994	4.010276	-1.358036
6	-2.376687	2.444641	-2.676289
1	-3.330803	1.978513	-2.946504
1	-1.984104	2.958653	-3.556270
6	-1.411536	1.389195	-2.143044
1	-1.441571	0.446133	-2.692585
1	-0.379553	1.759960	-2.145366
15	1.589278	0.060275	0.241393
7	2.688546	0.085313	1.454508
7	1.576689	1.420210	-0.677804
7	1.883791	-1.204270	-0.750271
1	-0.034438	0.880659	1.802263
6	2.549447	-0.457372	2.831346
1	2.390889	-1.543678	2.806518
1	1.713251	0.013756	3.355377
6	3.897309	-0.116726	3.470862
1	4.134931	-0.787221	4.298931
1	3.878048	0.908682	3.854608
6	4.877441	-0.226769	2.301640
1	5.830383	0.271646	2.488897
1	5.080710	-1.279501	2.074863
6	4.108056	0.418461	1.148437
1	4.230594	1.508192	1.139765
1	4.406089	0.026710	0.169131
6	2.555866	1.682892	-1.773871
1	2.033090	1.632254	-2.736906
1	3.357312	0.939234	-1.778071
6	3.027051	3.108454	-1.501016
1	3.424341	3.588334	-2.397826
1	3.809622	3.112643	-0.733969
6	1.755312	3.773391	-0.972059
1	1.937810	4.713218	-0.447384
1	1.065907	3.973860	-1.800938
6	1.166398	2.706230	-0.044788
1	1.594786	2.788754	0.963989
1	0.074529	2.762388	0.026908
6	2.647482	-2.398076	-0.282845
1	2.218759	-2.816020	0.634861
1	3.685810	-2.116026	-0.077677
6	2.551048	-3.366941	-1.467429
1	3.429253	-4.011836	-1.534263
1	1.670029	-4.010245	-1.358112
6	2.376718	-2.444573	-2.676324

1	3.330831	-1.978429	-2.946519
1	1.984148	-2.958569	-3.556321
6	1.411550	-1.389150	-2.143062
1	1.441582	-0.446073	-2.692580
1	0.379571	-1.759924	-2.145405

7

E(M062X) = -2724.08405233 a.u.

Atom	X	Y	Z
6	0.497968	-2.029248	0.256729
15	-0.840707	-1.350853	-0.540261
7	-0.589232	-1.030237	-2.175609
6	0.451293	-1.766400	-2.887495
15	1.969282	-1.143297	0.451146
7	2.139379	-0.718698	2.087853
6	1.325553	-1.252591	3.158672
7	3.315833	-2.193303	0.243357
6	3.582094	-2.611124	-1.125974
7	2.054775	0.027179	-0.586646
15	2.666987	1.450756	-1.128573
7	4.198277	1.611000	-0.211295
6	4.757396	2.949563	-0.338880
6	3.388466	-3.316330	1.163977
7	-2.089389	-2.535297	-0.510501
6	-2.014733	-3.606419	-1.497218
7	-1.471961	-0.006812	0.095691
15	-2.870556	0.656682	0.291852
7	-4.275835	0.033199	-0.374164
6	-4.357010	-0.011017	-1.833852
6	-2.386148	-3.054320	0.820991
7	-3.222522	0.663005	1.938544
6	-4.347957	1.468589	2.396363
7	-2.838225	2.187110	-0.386279
6	-1.583062	2.920327	-0.506191
6	-2.094906	0.686816	2.869337
6	-0.737432	0.319104	-2.710925
6	-4.018505	3.027201	-0.518616
6	-4.985902	-1.083132	0.241040
7	1.724984	2.653038	-0.312538
6	1.598220	3.962839	-0.913708
6	3.396046	-0.140287	2.536575
6	1.591420	2.637100	1.130129
6	5.181965	0.615126	-0.620104
1	0.372982	-2.995926	0.736403
1	0.128076	-1.932641	-3.923258
1	0.632921	-2.731917	-2.413035
1	1.391305	-1.198224	-2.882524
1	0.168239	0.917170	-2.529230
1	-1.591082	0.827397	-2.264646
1	-0.904648	0.245793	-3.792250
1	-2.968079	-4.148758	-1.499694
1	-1.209338	-4.327427	-1.277615
1	-1.855727	-3.182701	-2.490890
1	-3.360244	-3.558204	0.804530
1	-2.433450	-2.234226	1.545535
1	-1.636125	-3.783540	1.167259
1	-5.411657	-0.081828	-2.122843
1	-3.816419	-0.881913	-2.233226

1	-3.939862	0.898790	-2.269831
1	-6.064008	-0.943456	0.092330
1	-4.777522	-1.126301	1.311244
1	-4.684547	-2.031556	-0.221310
1	-4.078828	2.531921	2.494919
1	-4.671315	1.105202	3.378000
1	-5.188879	1.379740	1.703618
1	-1.721272	1.710372	3.028037
1	-1.280545	0.077217	2.476006
1	-2.425423	0.283806	3.833184
1	-1.537685	3.401913	-1.492447
1	-0.725267	2.249384	-0.409930
1	-1.517857	3.705967	0.261557
1	-3.990031	3.536060	-1.491030
1	-4.057400	3.798382	0.264339
1	-4.927790	2.424975	-0.470528
1	1.859286	-2.026455	3.737555
1	0.408810	-1.686037	2.754259
1	1.055168	-0.446843	3.856611
1	4.099039	-0.911404	2.894074
1	3.196066	0.550997	3.366633
1	3.861178	0.432678	1.728421
1	4.597534	-3.021842	-1.186255
1	3.511191	-1.751249	-1.796411
1	2.872249	-3.384435	-1.465894
1	4.384482	-3.770073	1.102537
1	2.644143	-4.099518	0.931372
1	3.230872	-2.977447	2.191067
1	4.943587	3.248300	-1.388664
1	5.714223	2.993217	0.195118
1	4.090339	3.686694	0.115272
1	5.446479	0.686559	-1.692564
1	4.805055	-0.389322	-0.411941
1	6.101671	0.755869	-0.037852
1	2.324050	4.695738	-0.521402
1	0.594798	4.373007	-0.726915
1	1.744225	3.886577	-1.996212
1	2.448997	3.108521	1.641223
1	1.499300	1.603018	1.477167
1	0.682457	3.182597	1.423697

7-H (C)

E(RM062X) = -2724.53516711 a.u.

Atom	X	Y	Z
6	0.223805	1.985925	0.743373
15	-1.054674	0.817061	1.298635
7	-0.398874	-0.390512	2.210729
6	0.513038	-0.034004	3.301795
15	1.588775	1.335914	-0.309885
7	0.919846	1.076942	-1.822097
6	-0.214161	1.766562	-2.413285
7	2.634511	2.651094	-0.461231
6	3.509650	2.973850	0.661750
7	2.226700	0.083603	0.323196
15	3.648549	-0.719477	0.740450
7	4.470143	-0.843075	-0.827105
6	5.516701	-1.865999	-0.786885
6	2.236066	3.815928	-1.238299

7	-1.933026	1.715057	2.413370
6	-2.669834	1.053848	3.490913
7	-1.840480	0.317151	0.024726
15	-2.883232	-0.626843	-0.691328
7	-4.122626	-1.256873	0.240825
6	-3.724992	-2.096618	1.374910
6	-2.623115	2.912905	1.940283
7	-3.559499	0.268577	-1.915485
6	-4.419841	-0.389824	-2.893784
7	-2.146279	-1.982532	-1.286715
6	-0.853915	-1.839993	-1.958397
6	-3.749749	1.711322	-1.835001
6	-0.162039	-1.738897	1.692468
6	-2.839935	-3.227998	-1.612022
6	-5.227945	-0.353104	0.559400
7	3.062345	-2.318062	0.869202
6	3.627814	-3.223311	1.851140
6	1.777588	0.408817	-2.801381
6	2.386410	-2.965124	-0.236086
6	5.061163	0.423158	-1.257533
1	-0.232979	2.859820	0.269102
1	0.407824	-0.772041	4.104207
1	0.261746	0.948873	3.709606
1	1.552472	-0.033534	2.946461
1	0.907975	-1.877342	1.509624
1	-0.707682	-1.897498	0.760347
1	-0.513979	-2.467115	2.434414
1	-3.691602	0.789552	3.182899
1	-2.736507	1.742247	4.339283
1	-2.151025	0.149548	3.810450
1	-3.605623	2.668779	1.508188
1	-2.036067	3.442027	1.187150
1	-2.775774	3.593790	2.782981
1	-4.607827	-2.622725	1.749111
1	-3.292152	-1.502450	2.194323
1	-2.987935	-2.837964	1.058305
1	-6.056029	-0.943822	0.960247
1	-5.577359	0.153889	-0.342018
1	-4.947611	0.403856	1.307777
1	-4.271455	0.077577	-3.872535
1	-5.482174	-0.303949	-2.624828
1	-4.167188	-1.448018	-2.978738
1	-3.513516	2.160859	-2.806307
1	-3.083413	2.131610	-1.080958
1	-4.788126	1.967206	-1.579849
1	-0.274274	-2.755354	-1.798816
1	-0.291583	-0.997214	-1.543638
1	-0.987709	-1.695831	-3.040041
1	-2.277200	-4.072404	-1.197149
1	-2.904545	-3.363171	-2.699736
1	-3.846671	-3.230686	-1.192814
1	0.103882	2.424452	-3.234598
1	-0.746643	2.366276	-1.674365
1	-0.926726	1.031881	-2.814507
1	2.299348	1.137403	-3.438642
1	1.156656	-0.227433	-3.444121
1	2.518629	-0.220485	-2.295398
1	4.423423	3.443723	0.281650

1	3.793555	2.064468	1.198206
1	3.032048	3.671771	1.367232
1	3.132648	4.374880	-1.524777
1	1.580466	4.497868	-0.671516
1	1.726580	3.509023	-2.153769
1	6.316212	-1.632321	-0.061600
1	5.974726	-1.942952	-1.778496
1	5.096100	-2.840991	-0.532343
1	5.857505	0.773029	-0.575499
1	4.299935	1.203887	-1.329184
1	5.505542	0.291972	-2.250851
1	4.338218	-3.934847	1.401865
1	2.829900	-3.805694	2.333336
1	4.154793	-2.652973	2.620960
1	3.068058	-3.575563	-0.848385
1	1.933303	-2.201837	-0.875223
1	1.592870	-3.626691	0.144332
1	0.736116	2.348577	1.644497

7-H (P)

E(M062X) = -2952.69517194 a.u.

Atom	X	Y	Z
6	0.486882	-2.004564	0.304530
15	1.981519	-1.207700	0.475952
7	2.113415	-0.031872	-0.624176
15	2.657003	1.411620	-0.865133
7	1.800767	2.660595	-0.183358
6	1.601043	2.670524	1.262854
15	-0.882415	-1.325084	-0.473804
7	-1.445243	0.046815	0.158104
15	-2.890663	0.677369	0.261353
7	-2.834331	2.183511	-0.471999
6	-4.022550	3.018052	-0.621849
7	-0.619224	-1.065856	-2.112874
6	-0.763155	0.240556	-2.734722
7	-2.123774	-2.490240	-0.381565
6	-2.410345	-2.963794	0.971513
6	0.320958	-1.911612	-2.838929
7	2.220760	-0.652380	2.045869
6	3.531173	-0.176235	2.471177
7	3.316074	-2.237218	0.246948
6	3.429022	-3.326270	1.217126
6	1.342841	-1.003426	3.151131
6	3.518019	-2.726788	-1.116038
7	4.267749	1.586184	-0.429987
6	5.208778	0.532193	-0.819109
6	4.846279	2.928330	-0.486238
6	-2.132794	-3.581579	-1.351983
7	-4.225479	0.000977	-0.468295
6	-4.986788	-1.085774	0.143588
7	-3.326894	0.714102	1.877603
6	-2.288148	0.740271	2.903677
6	-4.265929	-0.055240	-1.929786
6	-4.534979	1.434820	2.273030
6	-1.591207	2.940530	-0.513050
6	1.659555	3.953798	-0.839410
1	0.360905	-2.961797	0.804226
1	-0.068614	-2.109576	-3.844871

1	0.468306	-2.861056	-2.323059
1	1.297243	-1.412960	-2.924465
1	0.200635	0.774795	-2.743777
1	-1.493498	0.853081	-2.209055
1	-1.098079	0.112513	-3.771052
1	-3.102321	-4.088690	-1.294967
1	-1.345598	-4.327657	-1.156171
1	-2.013488	-3.186791	-2.362622
1	-3.396764	-3.439750	0.984604
1	-2.424929	-2.122992	1.673063
1	-1.674979	-3.704499	1.322210
1	-5.311137	-0.127250	-2.248070
1	-3.719054	-0.933234	-2.303702
1	-3.834422	0.848675	-2.363361
1	-6.055060	-0.915595	-0.033771
1	-4.806445	-1.123902	1.218556
1	-4.701007	-2.046673	-0.301624
1	-4.331919	2.500756	2.450849
1	-4.922057	0.999096	3.200191
1	-5.306767	1.344590	1.505418
1	-1.987601	1.770432	3.147967
1	-1.413723	0.189174	2.556393
1	-2.677278	0.271007	3.813802
1	-1.527071	3.470277	-1.472872
1	-0.735161	2.267691	-0.421813
1	-1.556681	3.690583	0.292509
1	-3.951542	3.572786	-1.564723
1	-4.114186	3.745400	0.196591
1	-4.922107	2.401218	-0.652997
1	1.800921	-1.767054	3.797973
1	0.395820	-1.385111	2.766554
1	1.147451	-0.113620	3.764129
1	4.098390	-0.961290	2.992522
1	3.403910	0.668488	3.160224
1	4.113831	0.168460	1.615670
1	4.523021	-3.155523	-1.191903
1	3.433020	-1.902850	-1.828092
1	2.784053	-3.501842	-1.384670
1	4.432576	-3.757819	1.151645
1	2.697587	-4.127626	1.024309
1	3.282676	-2.953227	2.233542
1	5.024073	3.270381	-1.517245
1	5.805410	2.914414	0.038614
1	4.198866	3.643853	0.023403
1	5.489746	0.599644	-1.880332
1	4.776238	-0.448750	-0.614097
1	6.115752	0.637324	-0.215731
1	2.359859	4.699148	-0.439290
1	0.641691	4.328039	-0.685906
1	1.830454	3.859075	-1.915619
1	2.407001	3.210075	1.780710
1	1.549684	1.643813	1.639208
1	0.649734	3.166109	1.485264
1	2.522297	1.728097	-2.228768

***t*Bu-P4**
E(M062X) = -2952.69517194 a.u.

Atom	X	Y	Z
15	0.130360	-0.081965	0.695772
15	-2.432081	-1.423556	-0.434487
15	-0.275746	2.697912	-0.113846
15	2.663476	-0.945589	-0.511443
7	0.575350	-0.859474	2.001166
7	-1.355963	-0.447818	0.065177
7	-2.602110	-2.830621	0.485145
7	-2.262482	-1.774720	-2.080857
7	-4.011530	-0.810996	-0.396584
7	0.080623	1.568711	0.902513
7	0.623195	4.052780	0.332547
7	0.049808	2.591257	-1.761210
7	-1.931528	3.039120	-0.145954
7	1.192166	-0.413680	-0.531014
7	3.177571	-2.099181	0.590488
7	3.769891	0.320585	-0.418391
7	2.943655	-1.735855	-1.968228
6	0.002132	-0.669295	3.328534
6	-1.534866	-0.560138	3.335728
1	-1.918469	-0.506404	4.363078
1	-1.990622	-1.422796	2.834237
1	-1.858084	0.340883	2.802265
6	0.391376	-1.896393	4.167557
1	1.481532	-2.004413	4.200144
1	-0.024832	-2.807671	3.721293
1	0.020816	-1.812441	5.197107
6	0.583127	0.587475	4.002912
1	0.229361	0.689801	5.037954
1	0.294928	1.480575	3.438999
1	1.678537	0.527920	4.011343
6	-1.389775	-3.452001	1.007935
1	-0.931381	-4.132229	0.270357
1	-1.650815	-4.041195	1.896453
1	-0.662417	-2.688002	1.302600
6	-3.621828	-3.803508	0.119933
1	-3.265362	-4.507614	-0.649204
1	-4.517609	-3.298711	-0.249667
1	-3.892883	-4.385835	1.009051
6	-0.921958	-2.106574	-2.547260
1	-0.167397	-1.622543	-1.918594
1	-0.799363	-1.754692	-3.580982
1	-0.750833	-3.196004	-2.536966
6	-3.311661	-2.414586	-2.859146
1	-3.190255	-3.508201	-2.895915
1	-3.273484	-2.039059	-3.890604
1	-4.293038	-2.183548	-2.440312
6	-4.195592	0.384499	-1.222888
1	-3.609091	1.232643	-0.836039
1	-5.258930	0.647423	-1.223020
1	-3.884144	0.187459	-2.251780
6	-4.533125	-0.592774	0.955349
1	-4.433930	-1.502029	1.550900
1	-5.593320	-0.329285	0.879533
1	-3.999031	0.219143	1.471737
6	1.159712	4.166896	1.681633

1	0.451706	4.665985	2.361900
1	2.081962	4.760509	1.650008
1	1.380886	3.174089	2.074124
6	0.315505	5.334145	-0.280231
1	-0.489290	5.866731	0.251577
1	0.017966	5.195046	-1.323069
1	1.210788	5.967370	-0.262197
6	1.447464	2.659759	-2.187252
1	1.961335	3.466574	-1.661117
1	1.467249	2.871974	-3.262908
1	1.977734	1.716622	-1.996923
6	-0.712659	1.614408	-2.542978
1	-0.277343	0.611338	-2.455545
1	-0.708482	1.929816	-3.593996
1	-1.743473	1.561705	-2.188231
6	-2.539661	3.960524	-1.092790
1	-2.636236	4.975698	-0.677782
1	-3.548766	3.604448	-1.343962
1	-1.951212	4.007501	-2.010766
6	-2.644505	2.971544	1.121580
1	-2.214756	2.182925	1.741047
1	-3.700318	2.739575	0.931434
1	-2.599732	3.927968	1.667849
6	2.353306	-3.301184	0.715585
1	2.994853	-4.148578	0.990080
1	1.868763	-3.525571	-0.240327
1	1.580296	-3.149632	1.478677
6	3.716786	-1.662863	1.877548
1	2.910212	-1.331182	2.541918
1	4.431353	-0.849400	1.735268
1	4.252190	-2.507897	2.326610
6	3.518937	1.463031	0.449565
1	4.059162	1.375464	1.405183
1	2.449933	1.551591	0.659746
1	3.855290	2.380623	-0.052327
6	5.170126	0.110302	-0.735809
1	5.589712	1.038480	-1.142839
1	5.277162	-0.671247	-1.490644
1	5.765945	-0.173626	0.146235
6	3.945203	-2.763949	-2.184706
1	4.765179	-2.395655	-2.821490
1	3.489072	-3.624066	-2.694001
1	4.359878	-3.096876	-1.232450
6	2.456056	-1.134606	-3.197736
1	1.632476	-0.453126	-2.978598
1	2.089531	-1.920134	-3.871771
1	3.253849	-0.579262	-3.716581

***t*Bu-P4**-H

E(M062X) = -2953.16941385 a.u.

Atom	X	Y	Z
6	-2.016254	1.456320	-2.461820
7	-2.472256	2.103145	-1.231262
6	-3.659469	2.934159	-1.428839
15	-1.280438	2.584481	-0.149832
7	-2.169229	2.886199	1.233430
6	-1.547355	3.621620	2.332693
7	-0.602314	4.068114	-0.505996

6	0.646787	4.168028	-1.255674
7	-0.080628	1.561684	-0.133259
15	0.033433	0.061894	0.477669
7	0.078230	0.320705	2.145668
6	0.581926	-0.575191	3.214848
6	0.589593	-2.038562	2.770119
7	-1.263663	-0.828798	0.153039
15	-1.689544	-2.232519	-0.395985
7	-2.835846	-1.995446	-1.607112
6	-3.373953	-3.152624	-2.324467
7	1.384829	-0.660684	-0.066078
15	2.817132	-0.157733	-0.500223
7	3.864866	-1.444797	-0.726673
6	3.829226	-2.171451	-1.990872
7	-2.276098	-3.152182	0.874669
6	-2.895153	-2.494375	2.022370
7	-0.594631	-3.245547	-1.139779
6	0.403414	-3.937476	-0.324772
7	2.736923	0.718799	-1.914496
6	3.925425	1.375686	-2.440942
7	3.658336	0.776957	0.600532
6	5.091810	0.705133	0.862262
6	-1.429772	5.266853	-0.595037
6	-3.106714	1.866540	1.712735
6	-2.768235	-4.509826	0.657156
6	-0.055430	-2.839244	-2.437989
6	-3.878678	-1.013960	-1.307105
6	1.644108	0.604993	-2.866985
6	3.072925	2.045468	1.019852
6	4.103952	-2.320044	0.419938
6	-0.348082	-0.406440	4.420586
6	2.007195	-0.159922	3.598830
1	-1.384357	0.600118	-2.220746
1	-1.469591	2.147638	-3.121703
1	-2.893361	1.092037	-3.004749
1	-3.951014	3.410457	-0.490719
1	-4.485523	2.295917	-1.763472
1	-3.497295	3.712214	-2.188479
1	-3.569317	1.349166	0.871371
1	-3.887958	2.359727	2.301183
1	-2.591422	1.126519	2.340859
1	-0.807252	4.327786	1.952872
1	-1.061611	2.938643	3.045257
1	-2.321662	4.177225	2.872766
1	-2.295805	-4.954699	-0.221186
1	-3.858597	-4.526722	0.522104
1	-2.522797	-5.124418	1.531108
1	-2.500120	-1.481710	2.126081
1	-2.665364	-3.062230	2.932312
1	-3.988043	-2.446088	1.912913
1	-0.799856	-2.273735	-3.002342
1	0.214006	-3.736347	-3.007039
1	0.840026	-2.217983	-2.304302
1	-0.027465	-4.250863	0.628058
1	1.259717	-3.278989	-0.130156
1	0.740591	-4.826424	-0.869377
1	-3.433123	-0.126226	-0.854441
1	-4.644966	-1.429219	-0.633045

1	-4.371801	-0.729577	-2.243349
1	-2.596264	-3.899377	-2.492652
1	-3.750033	-2.815964	-3.296370
1	-4.207059	-3.622978	-1.781954
1	0.801735	0.093962	-2.402295
1	1.954420	0.053604	-3.767269
1	1.314973	1.607168	-3.169253
1	4.670928	1.507360	-1.653829
1	3.655131	2.364058	-2.831289
1	4.380328	0.795406	-3.256517
1	2.004237	2.080120	0.796585
1	3.557129	2.883104	0.495321
1	3.219993	2.182267	2.098139
1	5.543320	-0.136852	0.338928
1	5.267651	0.597432	1.940587
1	5.585609	1.628629	0.528526
1	4.067915	-1.751758	1.352815
1	5.096854	-2.774045	0.326380
1	3.355050	-3.122032	0.477542
1	3.612559	-1.490547	-2.817358
1	3.073732	-2.971233	-1.981999
1	4.809737	-2.625952	-2.167506
1	2.671127	-0.227688	2.729370
1	2.397803	-0.803005	4.395288
1	2.022787	0.873556	3.968795
1	0.874200	-2.664805	3.622174
1	1.310515	-2.200408	1.963861
1	-0.394573	-2.364801	2.423574
1	0.006042	-0.995037	5.273907
1	-0.392983	0.644942	4.729521
1	-1.363384	-0.730094	4.169077
1	-2.323040	5.165077	0.024815
1	-0.855172	6.126173	-0.230166
1	-1.734572	5.474033	-1.630336
1	1.211039	3.238229	-1.164223
1	0.453647	4.372604	-2.318779
1	1.243873	4.993957	-0.851166
1	0.316754	1.289693	2.341453

References

1 J. F. Kögel, X. Xie, E. Baal, D. Gesevičius, B. Oelkers, B. Kovačević and J. Sundermeyer, *Chem. Eur. J.*, 2014, **20**, 7670.

2 J. Saame, T. Rodima, S. Tshepelevitsh, A. Kütt, I. Kaljurand, T. Haljasorg, I. A. Koppel and I. Leito, *J. Org. Chem.*, 2016, **81**, 7349.

3 a) Apex3, Bruker AXS Inc., Madison, Wisconsin, USA, 2016; b) SAINT, Bruker AXS Inc., Madison, Wisconsin, USA, 2015; c) SADABS. Bruker AXS area detector scaling and absorption correction, Bruker AXS Inc., Madison, Wisconsin, USA, 2016;

4 a) X-Area Pilatus3_SV, STOE & Cie GmbH, Darmstadt, Germany, 2016; b) X-Area Recipe, STOE & Cie GmbH, Darmstadt, Germany, 2015; c) X-Area Integrate, STOE & Cie GmbH,

Darmstadt, Germany, 2016; d) X-Area LANA, STOE & Cie GmbH, Darmstadt, Germany, 2016;

5 G. M. Sheldrick, *Acta Cryst.*, 2015, **A71**, 3.

6 G. M. Sheldrick, *Acta Cryst.*, 2015, **C71**, 3.

7 L. J. Farrugia, *J. Appl. Crystallogr.*, 2012, **45**, 849.

8 C. B. Hübschle, G. M. Sheldrick and B. Dittrich, *J. Appl. Crystallogr.*, 2011, **44**, 1281.

9 K. Brandenburg and H. Putz, Diamond - Crystal and Molecular Structure Visualization v4, Crystal Impact GbR, Bonn, Germany, 2014.

10 Jmol colors, http://jmol.sourceforge.net/jscolors/, (accessed 1 May 2019).

11 Gaussian 09, Revision D.01, M. J. Frisch, G. W. Trucks, H. B. Schlegel, G. E. Scuseria, M. A. Robb, J. R. Cheeseman, G. Scalmani, V. Barone, G. A. Petersson, H. Nakatsuji, X. Li, M. Caricato, A. Marenich, J. Bloino, B. G. Janesko, R. Gomperts, B. Mennucci, H. P. Hratchian, J. V. Ortiz, A. F. Izmaylov, J. L. Sonnenberg, D. Williams-Young, F. Ding, F. Lipparini, F. Egidi, J. Goings, B. Peng, A. Petrone, T. Henderson, D. Ranasinghe, V. G. Zakrzewski, J. Gao, N. Rega, G. Zheng, W. Liang, M. Hada, M. Ehara, K. Toyota, R. Fukuda, J. Hasegawa, M. Ishida, T. Nakajima, Y. Honda, O. Kitao, H. Nakai, T. Vreven, K. Throssell, J. A. Montgomery, Jr., J. E. Peralta, F. Ogliaro, M. Bearpark, J. J. Heyd, E. Brothers, K. N. Kudin, V. N. Staroverov, T. Keith, R. Kobayashi, J. Normand, K. Raghavachari, A. Rendell, J. C. Burant, S. S. Iyengar, J. Tomasi, M. Cossi, J. M. Millam, M. Klene, C. Adamo, R. Cammi, J. W. Ochterski, R. L. Martin, K. Morokuma, O. Farkas, J. B. Foresman, and D. J. Fox, Gaussian, Inc., Wallingford CT, 2016.
